# New MraY_AA_ Inhibitors with an Aminoribosyl Uridine Structure and an Oxadiazole

**DOI:** 10.3390/antibiotics11091189

**Published:** 2022-09-02

**Authors:** Hongwei Wan, Raja Ben Othman, Laurent Le Corre, Mélanie Poinsot, Martin Oliver, Ana Amoroso, Bernard Joris, Thierry Touzé, Rodolphe Auger, Sandrine Calvet-Vitale, Michaël Bosco, Christine Gravier-Pelletier

**Affiliations:** 1 Université Paris Cité, CNRS, Laboratoire de Chimie et de Biochimie Pharmacologiques et Toxicologiques, F-75006 Paris, France; 2 Unité de Physiologie et Génétique Bactériennes, Centre d’Ingénierie des Protéines, Département des Sciences de la Vie, Université de Liège, Sart Tilman, B4000 Liège, Belgium; 3 Institute for Integrative Biology of the Cell (I2BC), CNRS, Université Paris Sud, CEA, F-91405 Orsay, France

**Keywords:** MraY transferase, inhibitors synthesis, muraymycin analogs, molecular modeling, inhibition tests

## Abstract

New inhibitors of the bacterial transferase MraY from *Aquifex aeolicus* (MraY_AA_), based on the aminoribosyl uridine central core of known natural MraY inhibitors, have been designed to generate interaction of their oxadiazole linker with the key amino acids (H324 or H325) of the enzyme active site, as observed for the highly potent inhibitors carbacaprazamycin, muraymycin D2 and tunicamycin. A panel of ten compounds was synthetized notably thanks to a robust microwave-activated one-step sequence for the synthesis of the oxadiazole ring that involved the *O*-acylation of an amidoxime and subsequent cyclization. The synthetized compounds, with various hydrophobic substituents on the oxadiazole ring, were tested against the MraY_AA_ transferase activity. Although with poor antibacterial activity, nine out of the ten compounds revealed the inhibition of the MraY_AA_ activity in the range of 0.8 µM to 27.5 µM.

## 1. Introduction

Infectious diseases are one of the most important causes of human death, and bacterial antimicrobial resistance (AMR) is a major threat for human health [1,2,3]. Based on predictive statistical models, 4.95 million deaths are estimated to be associated with bacterial AMR in 2019, including 1.27 million deaths attributable to bacterial AMR [4]. In addition to its impact on human health [5], antibiotic resistance also has a significant economic cost, notably on the increase in hospital costs due to nosocomial infections [6,7,8,9]. One way to fight bacterial AMR is to focus on biological targets displaying a new mode of action compared to those of the approved antibiotics. Considering their high specificity and their unique occurrence in bacteria, enzymes involved in peptidoglycan biosynthesis [10,11] are promising targets, since each of them is essential for the bacterial growth. In this context, our goal is to focus on the inhibition of the MraY transferase [12], which is an unexploited target. Indeed, even if the discovery of potent MraY inhibitors is the subject of intense research efforts, none of the resulting compounds are being investigated in clinical trials. This represents a major advantage that can delay the emergence of drug resistance. This integral membrane protein, essential to bacterial growth and ubiquitous in the bacterial world [13,14,15], catalyzes the first membrane-associated step of peptidoglycan biosynthesis: the transfer of UDP-Mur*N*Ac-pentapeptide on undecaprenylphosphate (C_55_P) to form the lipid I. 

**Figure 1:**
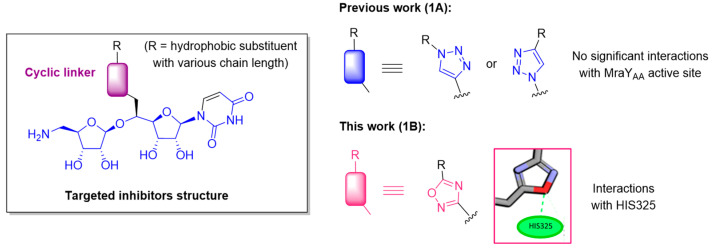
**1A.** Structure of the aminoribosyl-uridine-containing MraY inhibitors previously synthetized. **1B.** Structure of targeted new MraY inhibitors.

Several families of natural MraY inhibitors are known, notably the widely represented one of peptidonucleosidic antibiotics, such as liposidomycins [16,17,18], muraymycins [19] and caprazamycins [20,21]. All of these compounds share a common aminoribosyl uridine scaffold that has been shown to be important for their biological activity [22,23,24]. The synthesis of the simplified analogs of these complex natural compounds [25,26,27,28] retaining antibacterial activity is a challenge, and significant progress towards this goal has been made [29,30,31,32,33,34,35,36]. The crystallographic structure of MraY from *Aquifex aeolicus* (MraY_AA_) in complex with several ligands has been solved (muraymycin D2 (MurD2, PDB: 5CKR) [37] and carbacaprazamycin (PDB: 6OYH) [38]), permitting the S. Y. Lee group to define several hot spots (HS) of the priviledged interaction of the co-crystallized inhibitors with the MraY active site [38]. We also developed the synthesis of several series of inhibitors based on this aminoribosyl uridine core, the chemical diversity being introduced on the scaffold through various linkers, either cyclic such as *C-* and *N-*triazoles [39,40], or acyclic, such as urea [41], amide, sulfonamide, squaramide or diamide [42]. The docking of the triazole-containing inhibitors, in either the 5CKR [30] or 6OYH [31] structural models of MraY_AA_, revealed no significant interactions of the triazole with he aminoacids of the active site (Figure 1, **1A**). In the present work, considering this lack of stabilizing interaction, we decided to enlarge the panel of inhibitors with a cyclic linker. We focused on oxadiazole-containing ones because the docking of a representative of the targeted oxadiazole series, with a C_10_ chain as a hydrophobic substituent, showed the interaction of the oxadiazole ring with the key amino acids (H325) of the enzyme active site (Figure 1, **1B**), as observed for the highly potent inhibitors carbacaprazamycin, muraymycin D2 and tunicamycin. We hypothetized that this interaction could contribute to generate more active compounds. Furthermore, a panel of hydrophobic substituents with various chain length was selected to study their influence in promoting antibacterial activity.

## 2. Results and Discussion

### 2.1. Chemistry

**Figure 2:**
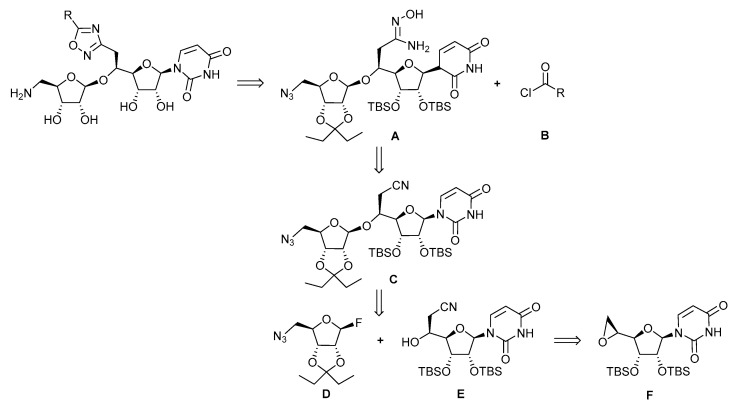
Retrosynthesis of the targeted oxadiazole inhibitors.

The retrosynthetic analysis towards the targeted compounds is outlined in Figure 2. It would rely on the tandem *O*-acylation-cyclization of the amidoxime **A** with various acyl chlorides **B**, either commercially available or synthetized. The amidoxime **A** could be obtained by a reaction of hydroxylamine with the nitrile **C** resulting from a diastereselective glycosylation between the nitrile alcohol **E** and a ribosyl donor **D**, activated as a fluoride in its anomeric position and bearing an azido group as a masked amine at C-5. Alcohol **E** should result from the nucleophilic opening of epoxide **F** resulting from uridine.

**Scheme 1:**
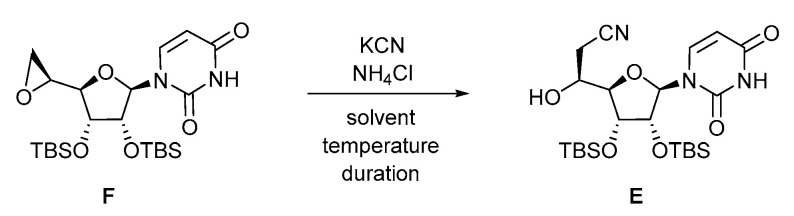
Nucleophilic opening of the epoxide F by cyanide ions.

The conveniently protected 5-azidoribosyl fluoride **D** was readily synthetized from D-ribose [43]. The synthesis of the cyano alcohol **E** [44] by nucleophilic opening by the potassium cyanide of the epoxide **F** [44] derived from uridine (Scheme 1) has been optimized (Table 1) by screening the number of equivalents of potassium cyanide, the solvent of the reaction, its duration and the eventual addition of ammonium chloride [45]. 

The previously described conditions [44,45] involving 8 equiv. of KCN and 1.5 equiv. of NH_4_Cl in DMF at 100 °C for 16 h (entry 1) led to variable percentages of the conversion and isolated yield (up to 68%) of compound **E** accompanied by the partial deprotection of the silyl ethers. Decreasing the amount of KCN to 6 equiv. and the temperature to 55 °C afforded a moderate 28% of the conversion. Replacing the solvent by refluxing CH_3_CN with KCN (6 equiv.) in the presence of NH_4_Cl (1.5 equiv., entry 3) or in its absence (entry 4) only led to poor conversion and degradation. The reaction was also assayed in toluene at 100 °C with KCN (6 equiv.) and NH_4_Cl (1.5 equiv., entry 5) or without NH_4_Cl (entry 6) for 16 h. However, only traces of the expected compound **E** were formed, and the reaction mixture turned out to be brownish, characteristic of silyl ethers deprotection. Finally, the reaction was attempted in a MeOH/H_2_O 8/1 mixture at 60 °C for 2 h in the presence of KCN (6 equiv.) and NH_4_Cl (1.5 equiv., entry 7), leading to an improved 84% isolated yield of compound **E**. Decreasing the amount of KCN (3 equiv.) and increasing the duration of the reaction to 16 h (entry 8) was detrimental to the progress of the reaction, leading to 62% of conversion. The best optimized conditions (entry 9) were revealed to be the use of KCN (5 equiv.) and NH_4_Cl (2.5 equiv.) in MeOH/H_2_O 8/1 at 60 °C for 16 h, giving cyanoalcohol E in a reproducible 94**%** isolated yield. 

**Scheme 2:**
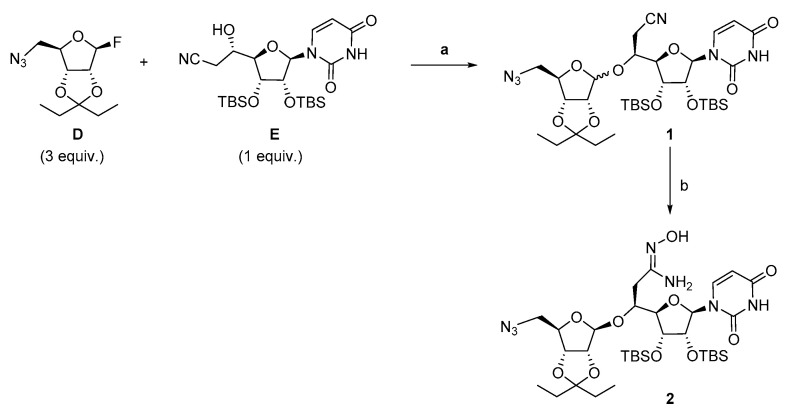
Synthesis of the amidoxime 2. Reagents and conditions: (**a**) 4Å MS, BF_3_.OEt_2_ (4.0 equiv.), −78 °C, 10 min then rt, 16 h, 67% of pure β isomer; (**b**) 50% aqueous NH_2_OH, MeOH reflux, 6 h, 87%.

We next turned to the diastereoselective glycosylation reaction of the phthalimidocyano alcohol **E** by the fluororibose derivative **D** as a ribosyl donor (Scheme 2). This reaction was performed according to the conditions we previously described [40]. However, the number of equivalents of the reaction partners has been optimized. The best conditions involved one equivalent of the phthalimido alcohol E and three equivalents of fluorinated azido-ribose **D** that were dried by the azeotropic removal of water with toluene. Then, an excess of 4 Å molecular sieves was added after activation by vacuum drying. The products were dissolved in freshly distilled DCM before the addition of boron trifluoride etherate (4.0 equiv.) at −78 °C. After ten minutes at −78 °C, followed by sixteen hours at room temperature, the crude product 1 was isolated as a 8/2 mixture of β/α diastereoisomers. After separation by flash chromatography, the pure β isomer of the glycosylated compound **1** was obtained in 67% yield. This derivative 1 was then engaged in the formation of the amidoxime 2 by reaction with 50% aqueous hydroxylamine in refluxing methanol, leading to the expected amidoxime 2 in 87% yield.

**Scheme 3:**
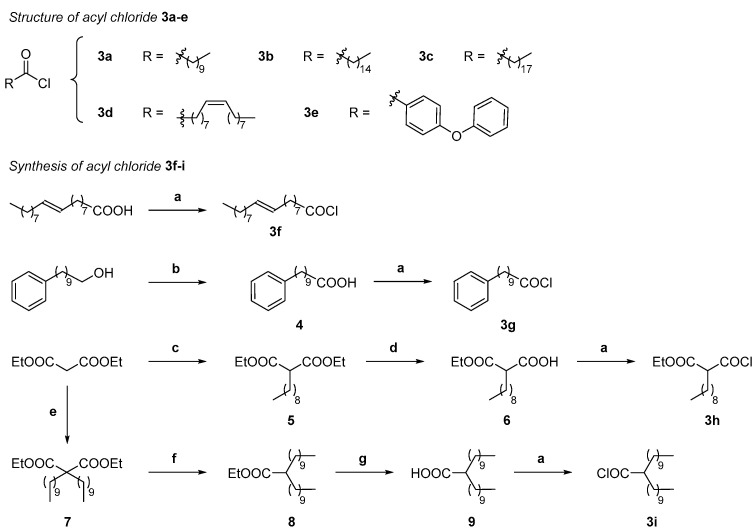
Structure and/or synthesis of compounds **3a**–**i**. Reagents and conditions: (**a**) Oxalyl chloride, dry DMF cat., pentane, r.t., 2 h; (**b**) CrO_3_, H_2_SO_4_, H_2_O, acetone, 0 °C to r.t., 3 h, 80%; (**c**) CH_3_(CH_2_)_8_I (1 equiv.), K_2_CO_3_ (5 equiv.), DMF/THF 1/1, overnight, r.t. to 90 °C to r.t., 91%; (**d**) (1) KOH (1 equiv.), EtOH/H_2_O 10/1, (2) Aqueous HCl, H_2_O, 72%; (**e**) NaH (3 equiv.), 0 °C 30 min, then CH_3_(CH_2_)_9_I (2.5 equiv.), THF, 0 °C to r.t., 48 h, 76%; (**f**) LiCl (2.5 equiv.), H_2_O (1.15 equiv.), DMSO, 12 h, r.t., 60%; (**g**) NaOH, EtOH/H_2_O 10/1, overnight (90%).

Starting from the amidoxime **2** intermediate, the synthesis of a series of MraY inhibitors with an oxadiazole linker was then undertaken by the *O*-acylation of the amidoxime **2** with a panel of acyl chlorides **3** (Scheme 3), either commercially available, with various alkyl chain lengths, an eventual unsaturation or aromatic moieties (**3a–3e**), or synthetized according to classical routes (**3f**–**3i**). 

The choice of the acyl chloride substituents was directed towards long hydrophobic chains (C_10_–C_18,_ **3a**–**3c**, **3f**) in aiming at filling the hydrophobic groove HS4 of the MraY active site [38] and at obtaining antibacterial activities, since we previously showed that inhibitors with small chains are probably not sufficient to lead to good antibacterial activities [41]. A diphenyl ether (**3e**) was chosen in agreement with the reported inhibitory activity of MraY [46], and a C_9_-alkyl chain bearing a terminal phenyl group was also selected (**3g**). To potentially fill two hot spots of the active site (HS2 and HS4), disubstituted compounds bearing a C_9_ alkyl chain and an ethyl ester (**3h**) and two C_10_ alkyl chains (**3i**) were also targeted. The synthesis of the acyl chlorides **3f–3i** (Scheme 3) was realized by the reaction of the corresponding commercial or synthetized carboxylic acids with oxalyl chloride in the presence of catalytic DMF in pentane [47]. Accordingly, the acyl chloride **3f** bearing a long C_18_ alkyl chain with an *E* unsaturation was performed from the corresponding commercial acid. The 10-phenyl decanoyl chloride **3g** was obtained by the Jones oxidation of the corresponding alcohol into the carboxylic acid **4** that was isolated in 80% yield. The synthesis of the derivative **3h** was carried out by reacting the anion of diethyl malonate generated in the presence of an excess of potassium carbonate in a 1/1 DMF/THF mixture with 1-iododecane, leading to the diester, followed by the selective saponification of the one ester group [48] by potassium hydroxide in a 1/10 H_2_O/EtOH mixture, giving the mono acid mono ester **6** in 72% yield. The dialkylated acid **9** resulted from a three-step sequence involving first the dialkylation by 1-iododecane of diethyl malonate in the presence of sodium hydride in THF [49] that was achieved in 78% yield and led to the dialkylated derivative **7**. Then, the decarboethoxylation of this compound **7** in the presence of lithium chloride and H_2_O in DMSO [50] afforded the monoethyl ester **8,** which was followed by the saponification of the ester in the presence of sodium hydroxide in a 10/1 EtOH/H_2_O mixture, giving the dialkylated acid **9**. After treatment of the acids **4**, **6** and **9** with oxalyl chloride as previously mentioned, the resulting crude acyl chlorides **3g**–**3i** were sufficiently pure to be engaged in the subsequent *O*-acylation step of amidoxime **2** without further purification.

**Scheme 4:**
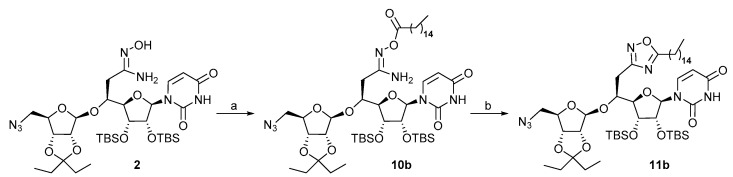
Synthesis of the oxadiazole **11b**. Reagents and conditions: (**a**) DBU (2 equiv.), 3b (2 equiv.), CH_2_Cl_2_, 0 °C, 15 min, then reflux overnight, 45%; (**b**) toluene, µW 300 W, 135 °C, 3 h, 82%.

With the amidoxime **2** and acyl chlorides **3a**–**3i** in hand, we focused on the synthesis of the oxadiazole-containg inhibitors (Scheme 4). The reaction was first attempted in a two-step sequence involving first the *O*-acylation of the amidoxime **2** with palmitoyl chloride **3b** in the presence of DBU in refluxing CH_2_Cl_2_ leading to compound **10b** in 45% yield, followed by cyclization in toluene under microwave irradiation at 300 W for 3 h at 135 °C. After purification, the oxadiazole **11b** was isolated in 82% yield. 

**Scheme 5:**
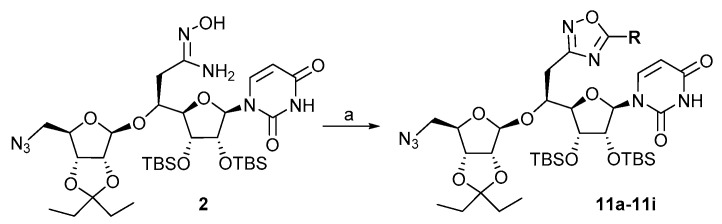
Synthesis of the oxadiazoles **11a**–**11i**. Reagents and conditions: (**a**) **3a**–**3i** (1.1 equiv.), DBU (see Table 2) 30 min, r.t. then, µW 150 °C, toluene.

It has to be mentioned that the cyclization reaction performed in thermic conditions involving 4 days of heating in reluxing toluene only led to traces of the expected compound **11b** and degradation, while part of the starting *O*-acylated amidoxime **10b** was still present. In order to improve the yield and shorten the duration of this two-step sequence, the reaction was then carried out according to a one-pot procedure under microwave irradiation (Scheme 5, Table 2) at 150 °C in toluene in the presence of variable amounts of DBU for different durations.

The reaction was first performed in the presence of DBU (2 equiv.) for 15 min leading to the oxadiazole **11b** in a modest 28% yield (entry 1). Increasing the amount of DBU (2.5 equiv.) slightly improved the yield of the reaction to 30% (entry 2). Finally, the best conditions involved DBU (2.5 equiv.) for 30 min under microwave irradiation at 150 °C in toluene and led to oxadiazole **11b** in 55% yield. Partial degradation was observed with longer reaction time, thus decreasing the yield. These conditions were then applied to the synthesis of the oxadiazoles **11a** and **11c**–**11i** that were isolated in yields ranging from 42 to 83% (Table 3). 

**Scheme 6:**
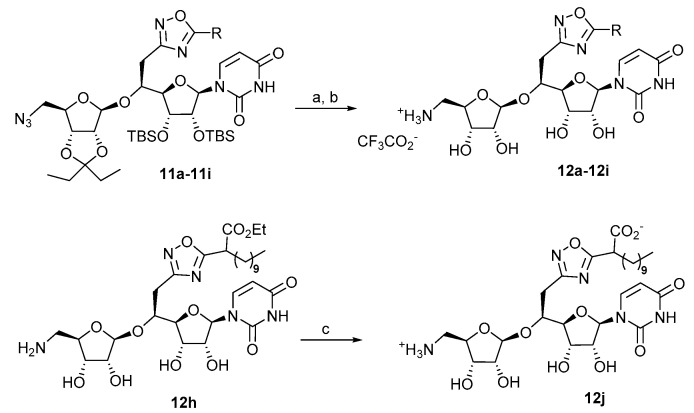
Deprotection steps of compounds **11a**–**11i** and **12h**. Reagents and conditions: (**a**) PS-PPh_3_, THF/H_2_O 85/15, r.t., 48 h; (**b**) TFA/H_2_O 4:1, 0 °C, r.t., 16 h; (**c**) aqueous NH_4_HCO_3_, Et_3_N, MeOH, 17%.

The reduction of the azido group of oxadiazoles **11a**–**11i** was then performed in a 85/15 THF/H_2_O mixture under Staudinger conditions using polymer-supported triphenylphosphine to efficiently remove the generated supported triphenylphosphine oxide by filtration. The final acidic hydrolysis of the alcohol protective groups of the resulting crude amines was carried out in a cold 4/1 mixture of trifluoroacetic acid/water. The targeted oxadiazoles inhibitors **12a**–**12i** were isolated in 22 to 85% overall yield after flash chromatographic purification on silica gel (Scheme 6, Table 3). The saponification of ester **12h** (Scheme 6) was achieved by ammonium hydrogenocarbonate in the presence of triethylamine in methanol leading to the oxadiazole **12j** (Scheme 6).

### 2.2. Biological Studies

**Figure 3:**
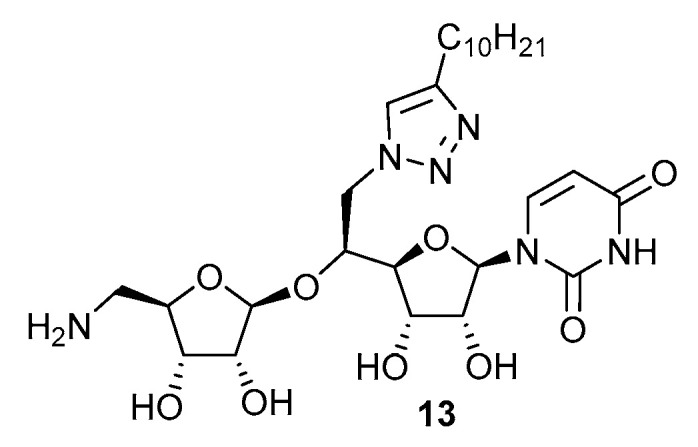
Structure of the reference *N*-triazole inhibitor **13**.

The inhibitory activity of the synthesized compounds **12a**–**12j** was evaluated on MraY transferase purified from *Aquifex aeolicu*s (MraY_AA_) prepared as previously described by Chung et al. [51]. Their activity (Table 4) was compared to the inhibitory activity of the *N-*triazole-containing inhibitor 13 (Figure 3) that we previously synthesized [40] (Table 4). Commercially available tunicamycin from *Streptomyces* sp. was used as a positive control in the test.

As shown in Table 4, nine oxadiazole-containing compounds out of the ten tested are relevant inhibitors of the enzymatic activity of the transferase MraY_AA_ with IC_50_ ranging from 0.8 µM to 27.5 µM, the best MraY_AA_ inhibitory activity being obtained with a saturated C_10_ alkyl substituent on the oxadiazole moiety (**12a**). The inhibitory activity of this compound **12a** (0.8 µM) is a bit improved compared to that of the reference *N*-triazole **13** (1.1 µM). Among the compounds with one alkyl chain (**12a**,**12b**, **12c**), the results show that increasing the alkyl chain length from a C_10_ chain to C_15_ and C_18_ ones is detrimental to inhibitory activity, with IC_50_ decreasing by a factor of 12 and 20, respectively. The introduction of an unsaturation within the alkyl chain (compounds **12d** and **12f**) leads to an inhibitory activity in the same range as that of a saturated compound (**12c**). The configuration of the double bond did not display a significant difference among the obtained IC_50_ with IC_50_ equal to 18.9 µM for the *Z*-unsaturated compound (**12d**), as compared to 17.3 µM for the *E*-unsaturated one (**12f**) and 15.8 µM for the saturated one (**12c**). The potential filling of the MraY_AA_ hot spots (HS) by aromatic substituents was rather satisfying, with IC_50_ in the low µM range (3.2 µM and 5.8 µM) for compounds **12e** and **12g**, respectively. The results concerning the tentative filling of two MraY_AA_ HS by two substituents are contrasted. Indeed, the compound **12i** with two C_10_ alkyl chain revealed no activity, showing that it is probably too hindered for correct positioning within the MraY_AA_ active site, while the oxadiazole, with both a C_9_ alkyl chain and an ethyl ester, was moderately active with an IC_50_ equal to 27.5 µM. More interestingly, the corresponding carboxylate **12j** is almost tenfold more active (IC_50_ equal to 2.9 µM) than the parent ester, probably due to its better positioning in the MraY_AA_ active site.

The antibacterial activity of MraY inhibitors **12a**–**12h** was also evaluated against several bacterial strains. Gram-negative (*E. coli* ATCC 8730, *C. freundii* ATCC8090 and *P. aeruginosa* ATCC 27853) and Gram-positive pathogenic bacterial strains (*S. aureus* ATCC 25923 and *E. faecium* ATCC 19434), including a methicillin-resistant strain (*S. aureus* MRSA ATCC 43300), were selected as a representative of pathogen bacterial diversity. Piperacillin and vancomycin were used as positive control in the tests. The results (See Table S1, Supplementary Material) show that, although our previous data on differently substituted urea-containing inhibitors [41], suggested that a linear chain of at least 12 carbon atoms, or a branched substituent, is required to get antibacterial activity, almost none of the oxadiazole inhibitors displayed antibacterial activity, except a modest 50 µg/mL activity for two long-chain-containing compounds, **12c** (C_18_) and **12f** (C_17_ with a *E* unsaturation), on the three Gram-positive bacterial strains. This disappointing result demonstrates that the further optimization of the lipophilic side chain is still required to increase the antibacterial activity.

### 2.3. Docking Studies

Molecular modeling studies were performed to rationalize the SAR results for this novel series of MraY_AA_ inhibitors. CDOCKER [52] was used to dock the compounds into the X-ray structures of the MraY_AA_ enzyme in open and close conformations (PDB ID: 5CKR and PDB ID: 6OYH, respectively), following the procedure described previously [41,42]. The top 50 best docking poses, based on the CDOCKER interaction energy function, were retained and then further visually examined.

**Figure 4:**
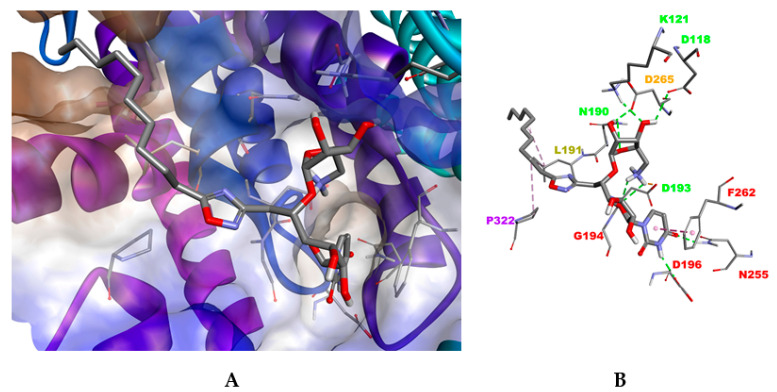
Docking pose for a compound in the closed conformation of MraY_AA_ (PDB ID: 6OYH). (**A**). The hydrophobic surface is rendered as brown and the hydrophilic surface as blue. Ligand and residues are shown in stick mode. (**B**). Interactions between the inhibitor **12a** and MraY_AA_ are shown as colored dashed lines: hydrophobic interactions (magenta), electrostatic interactions (orange) and hydrogen bond (green). For clarity, the apolar hydrogen atoms are omitted.

In the closed conformation of MraY_AA_ (PDB ID: 6OYH), compounds **12a**–**12d** and **12f**–**12h** exhibit a binding mode similar to that of triazole compound **13** (Figure 4). The uracil moiety forms an H-bond with the key residue D196 and π–π stacking interactions with F262 within the small uracil binding pocket. The lipophilic tail anchors deeply in the hydrophobic groove HS4 predicted to be the C_55_P substrate binding pocket [38] establishing hydrophobic contacts with P322 and L191 in HS2 and HS6 pockets, respectively. In addition, an extended network of H-bond and electrostatic interactions was retrieved between the aminoribosyl part and residues N190 and D193 located in the HS1 domain. No stabilizing H-bond interactions were predicted between the oxadiazole moiety and polar residues H324 and H325 within the HS2 area. Nevertheless, docking in the closed conformation of MraY_AA_ failed to discriminate between active **12a** and moderately active compounds (**12b**–**d**, **12f**–**h** and **12j**). Moreover, rigid or branched tail compounds (**12e** and **12i**, respectively) failed to assume this binding mode due to steric hindrance with the residues of the active site.

**Figure 5:**
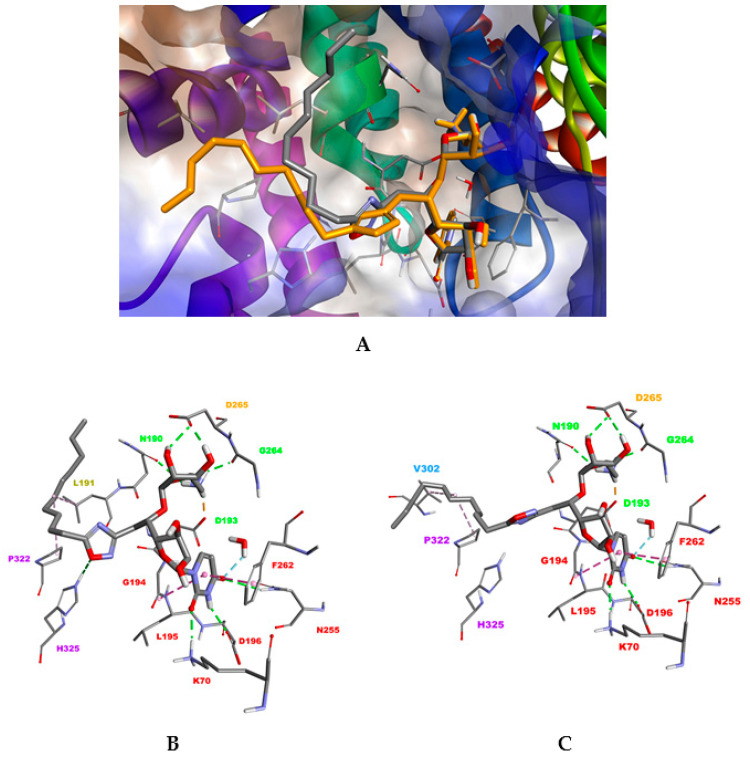
(**A**) Superimposition of the two docking conformations I and II, obtained for compound **12a** in the open conformation of MraY_AA_ (PDB ID: 5CKR). (**B**) Binding mode I. (**C**) Binding mode II. Ligand and residues are shown in stick mode. Interactions between the inhibitor **12a** and MraY_AA_ are shown as colored dashed lines: hydrophobic interactions (magenta), electrostatic interactions (orange) and hydrogen bond (green). For clarity, the apolar hydrogen atoms are omitted.

The best docking results were obtained in the open conformation of MraY_AA_ (PDB ID: 5CKR). Although all compounds target the uridine- and uridine-adjacent pockets, as previously seen in closed conformation, significant differences were observed in the interaction network. Two possible binding conformations (I, II) were obtained for the most active compound, **12a** (Figure 5A). Both are characterized by two new stabilizing interactions between the uracil moiety and residues K70 and N255 but differ in the spatial orientation of the lipophilic tail, located either in the plane of the membrane (binding mode I, Figure 5B) or exposed in the cytoplasm (binding mode II, Figure 5C). Moreover, a supplementary H-bond with the residue H325 was also predicted for the oxadiazole linker, only for binding mode I. The other compounds bind to the MraY_AA_ active site according to the binding mode II only. For compounds **12e** and **12g**, the interactions network with the aminoribosyluridine part remained similar to that of compound **12a**. Nevertheless, the H-bond interaction of H325 with the oxadiazole moiety was lost, reducing the stability of the compound within the binding site and consequently its activity. Interestingly, the loss of this H-bond was fairly compensated by hydrophobic contacts between the lipophilic tail and residues L191 (HS6) and F309 and between A321 (HS2) and V302 (HS4). 

For compound **12b**, interaction with H325 seems to be detrimental to the appropriate orientation of the long hydrophobic tail within the HS2 pocket. Less active compounds **12c**, **12d** and **12f** lose at least two significant H-bonds involving N255 in the uridine binding pocket and H325 (HS2). For the branched compound **12i**, the HS2 area can accomodate only one branch, leaving the other one exposed towards the solvent surface. Concerning the compounds **2h** and **2j** with both a C9 alkyl chain and either an ethyl ester or a carboxylate moiety, no clear conclusions could be drawn from docking results. Taken together, these results suggest that tight interactions of compound **12a** with residues within the uracil binding pocket, uridine like and HS2 domain in the open conformation of MraY_AA_ could reduce its flexibility providing an explanation for its better activity. 

## 3. Materials and Methods

### 3.1. Chemical Synthesis

The reactions were carried out under an argon atmosphere, if required, and were monitored by thin-layer chromatography. All microwave-mediated reactions were carried out in G10 or G20 Anton Paar vials, using an Anton Paar Monowave 300 Extra microwave synthesizer. Flash chromatography was performed with silica gel 60 (40–63 μm). Spectroscopic ^1^H and ^13^C NMR, MS and/or analytical data were obtained using chromatographically homogeneous samples. ^1^H NMR (500 MHz) and ^13^C NMR (125 MHz) spectra were recorded in CDCl_3_ unless otherwise indicated. Chemical shifts (δ) are reported in ppm, and coupling constants are given in Hz. For each compound, detailed peak assignments have been made according to COSY, HSQC and HMBC experiments. The numbering of molecules is indicated in the Supplementary Material. Optical rotations were measured with a sodium (589 nm) lamp at 20 °C. IR spectra were recorded on an FT–IR spectrophotometer, and the wavelengths are reported in cm^−1^. High-resolution mass spectra (HRMS) were recorded with a TOF mass analyzer under electrospray ionization (ESI) in positive ionization mode detection [42].

#### 3.1.1. Protected Nitrile **1**

To a solution of **E** (100 mg 0.19 mmol, 1 equiv.) and **D** (192 mg, 78 mmol, 4 equiv.) in freshly distillated DCM (7 mL) was added 4 Å molecular sieves (1.4 g). After stirring at r.t. for 1 h, boron trifluoride diethyl etherate (96 µL, 0.78 mmol, 4 equiv.) was added at –80 °C. After 15 min at –80 °C, the cooling bath was removed, then the mixture was allowed to warm to r.t. for 16 h. The mixture was filtrated over a pad of celite, then washed with EtOAc. The filtrate was treated with a saturated aqueous solution of sodium bicarbonate. The organic layer was washed with water and brine, then concentrated in vacuo. Flash chromatographic purification (cyclohexane/EtOAc 75:25) afforded the product as a white solid (96 mg, 67%): R*_f_* 0.17 (cyclohexane/EtOAc 7/3); [α]_D_ + 94.1 (*c* 1.0, CH_2_Cl_2_); IR (film) 3675, 2971, 2900, 1696, 1684, 1394, 1229, 1076, 1047, 891, 869; ^1^H NMR (500 MHz, CDCl_3_) *δ* 8.58 (br s, 1H, NH), 7.73 (d, *J*_H6-H5_ = 8.2 Hz, 1H, H_6_), 5.74–5.53 (m, 2H, H_5_ + H_1′_), 5.11 (s, 1H, H_1″_), 4.53 (d, *J*_H2″-H3″_ = 6.2 Hz, H_2″_), 4.43 (d, *J*_H3″-H2″_ = 6.2 Hz, 1H, H_3″_), 4.31 (t, *J*_H4″-H5″b_ = *J*_H4″-H5″a_ = 5.4 Hz, 1H, H_4″_), 4.17–4.03 (m, 2H, H_4′_ + H_2′_), 3.98–3.84 (m, 2H, H_3′_ + H_5′_), 3.45–3.40 (m, 2H, H_5″a_, H_5″b_), 2.93–2.80 (m, 2H, H_6′b_, H_6′a_), 1.60 (q, *J* = 7.4 Hz, 2H, H_7″_), 1.46 (q, *J* = 7.4 Hz, 2H, H_7″_), 0.87 – 0.74 (m, 24H, -C(CH_3_)_3_, H_8″_), 0.16–0.04 (m, 12H, -Si(CH_3_)_2_); ^13^C NMR (125 MHz, CDCl_3_) *δ* 162.9 (C_4_), 149.95 (C_2_), 140.15 (C_6_), 118.12 (C_6″_), 116.63 (C_7′_), 112.34 (C_1″_), 101.56 (C_5_), 90.20 (C_1′_), 86.00 (C_3″_), 85.49 (C_4″_), 84.21 (C_4′_), 81.56 (C_2″_), 75.23 (C_5′_), 75.05 (C_2′_), 71.05 (C_3′_), 53.45 (C_5″_), 29.18 (C_7″_), 28.85 (C_7″_), 25.80 (-C(CH_3_)_3_), 21.72 (C_6′_), 18.0, 17.9 (-C(CH_3_)_3_), 8.36 (C_8″_), −4.0, −4.3, −4.9, −4.9 (-Si(CH_3_)_2_); HRMS ESI+ Calcd. for C_33_H_57_N_6_O_9_Si_2_^+^ (M + H)^+^ 737.3720, found 737.3731.

#### 3.1.2. Protected Amidoxime **2**

The mixture of nitrile **1** (363 mg, 0.49 mmol, 1 equiv.) and 50% aqueous hydroxylamine (0.23 mL, 3.94 mmol, 8 equiv) in MeOH (8 mL) was heated at reflux for 6 h, then concentrated in vacuo. The oily residue was diluted with EtOAc (30 mL), then washed with water and brine (2 × 10mL). The organic layer was dried (MgSO_4_), then concentrated in vacuo. Flash chromatographic purification (cyclohexane/EtOAc 75:25) afforded product **2** as a white solid (329 mg, 87%): R*f* 0.25 (cyclohexane/EtOAc = 1/1); [α]_D_ +13 (c 1.0, CH_2_Cl_2_); IR (film): 1260, 1165, 1091, 911, 834, 735. ^1^H NMR (500 MHz, CDCl_3_) *δ* 7.75 (d, *J* _H6-H5_ = 8.1 Hz, 1H, H_6_), 5.88 (d, J_H1′-H2′_ = 5.5 Hz, 1H, H_1′_), 5.73 (d, J_H5-H6_ = 8.2 Hz, 1H, H_5_), 5.31 (s, 1H, H_1″_), 4.62 (dd, J_H3″-H2″_ = 6.3 Hz, J_H3″-H4″_ = 1.7 Hz, 1H, H_3″_), 4.53 (d, J_H2″-H3″_ = 6.3 Hz, 1H, H_2″_), 4.34 (td, *J*_H4″-H5″_ = 5.6 Hz, *J*_H4″-H3″_ = 1.7 Hz, 1H, H_4″_), 4.25 (t, *J*_H5′-H4′_ = 3.0 Hz, 1H, H_5′_), 4.16-4.09 (m, 2H, H_2′_, H_4′_), 4.05 (t, *J*_H3′-H2′_ = 3.8 Hz, 1H, H_3′_), 3.49 (dd, *J*_H6a′-H5′_ = 12.8 Hz, *J*_H6a′-H6b′_ = 5.6 Hz, 1H, H_6a′_), 3.45 (dd, *J*_H6b′-H5′_ = 12.8 Hz, *J*_H6b′-H6a′_ = 5.6 Hz, 1H, H_6b′_), 2.80 (dd, *J*_H5a′-H6′_ = 14.3 Hz, *J*_H5a′-H5b′_ = 5.5 Hz, 1H, H_5a′_), 2.52 (dd, *J*_H5b′-H6′_ = 14.3 Hz, *J*_H5b′-H5a′_ = 8.8 Hz, 1H, H_5b′_), 1.69 (q, *J*_H7a″-H8″_ = 7.4 Hz, 2H, H_7a″_), 1.55 (q, *J*_H7b″-H8″_ = 7.5 Hz, 2H, H_7b″_), 0.91-0.83 ( m, 24 H, H_8”_, SiCH_3_), 0.10-0.02 (m, 12 H, Si(CH_3_)_2_). ^13^C NMR (125 MHz, CDCl_3_) δ 171.8 (C_7′_), 162.8 (C_4_), 150.1 (C_2_), 140.5 (C_6_), 111.8 (C_1″_), 102.1 (C_5_), 89.0 (C_1′_), 86.2 (C_2″_), 86.1 (C_4″_), 84.8 (C_4′_), 81.5 (C_5′_), 75.3 (C_2′_), 72.3 (C_3′_), 53.6 (C_5″_), 38.7 (C_6′ab_), 29.4 (C_7″_), 29.0 (C_7″_), 25.93, 25.86 (-C(CH_3_)_3_), 18.1 (-C(CH_3_)_3_), 8.5, 7.7 (C_8″_), −4.1, −4.6, −4.8, (-SiC); HRMS ESI+ calcd. for C_33_H_60_N_7_O_10_Si_2_^+^ (M + H)^+^ 770.3935 found 770.3914.

#### 3.1.3. General Procedure for the Synthesis of Protected Oxadiazoles

In a 10 mL microwave reaction vial, to a solution of amidoxime **2** (1 equiv.) in toluene (3 mL) was added acyl chloride **3a**–**3i** (1.1 equiv.) and DBU (2.5 equiv.). The reaction was stirred at r.t. for 30 min. Then the reaction mixture was irradiated under microwave irradiation at 150 °C for 30 min to 1 h. The reaction mixture was then concentrated in vacuo. Flash chromatographic purifications (cyclohexane/EtOAc 9:1) afforded the products **11a–11i** in 42 to 83% yield.

#### 3.1.4. Protected Oxadiazole **11a**

Compound **11a** was obtained as a white solid (40 mg, 67% yield): R*_f_* 0.25 (cyclohexane/EtOAc = 7/3); [α]_D_ +14 (c 1.0, CH_2_Cl_2_); IR (film): 3006, 2873, 1558, 1427, 1368, 1275, 1146, 1016, 988, 935, 887, 820, 764, 750; ^1^H NMR (500 MHz, CDCl_3_) *δ* 7.89 (d, *J*_H6-H5_ = 8.2 Hz, 1H, H_6_), 5.85 (d, J_H1′-H2′_ = 4.7 Hz, 1H, H_1′_), 5.73 (dd, *J*_H5-H6_ = 8.2 Hz, 1H, H_5_), 5.24 (s, 1H, H_1″_), 4.64 (dd, *J*_H3″-H2″_ = 6.2 Hz, *J*_H3″-H4″_ = 1.5 Hz, 1H, H_3″_), 4.52 (d, *J*_H2″-H3″_ = 6.2 Hz, 1H, H_2″_), 4.32 (t, *J*_H5′-H4′_ = 5.8 Hz, 1H, H_5′_), 4.24–4.21 (m, 1H, H_4″_), 4.16 (t, *J*_H2′-H3′_ = 4.7 Hz, 1H, H_2′_), 4.05 (dd, *J*_H4′-H3′_ = 4.4 Hz, *J*_H4′-H5′_ = 1.6 Hz, 1H, H_4′_), 3.99 (t, *J*_H3′-H4′_ = 4.4 Hz, 1H, H_3′_), 3.50 (dd, *J*_H6′a-H5′_ = 12.7 Hz, *J*_H6′a-H6′b_ = 5.2 Hz, 1H, H_6′a_), 3.43 (dd, *J*_H6′b-H5′_ = 12.7 Hz, *J*_H6′b-H5′_ = 5.2 Hz, 1H, H_6′b_), 3.38 (dd, *J*_H5″a-H4″_ = 15.0 Hz, *J*_H5″a-H5″b_ = 9.3 Hz, 1H, H_5″a_), 3.19 (dd, *J*_H5″b-H4″_ = 15.0 Hz, *J*_H5″b-H5″a_ = 9.3 Hz, 1H, H_5″b_), 2.83 (t, *J*_H2′-H3′_ = 7.6 Hz, 1H, H_1*_), 1.80–1.74 (m, 2H, H_2*_), 1.69 (q, *J*_H7″-H8″_ = 7.5 Hz, 2 H, H_7″_), 1.56 (q, *J*_H7″-H8″_ = 7.5 Hz, 2 H, H_7″_), 1.30–1.24 (m, 24H, H_3*_-H_14*_), 0.92–0.82 (m, 27H, -SiC(CH_3_)_3_, H_8″_, H_15*_), 0.08–0.05 (12H, Si(CH_3_)_2_); ^13^C NMR (125 MHz, CDCl_3_) *δ* 180.2 (C_8′_), 166.9 (C_7′_), 163.0 (C_4_), 150.1 (C_2_), 140.2 (C_6_), 118.00 (C_6″_), 112.2 (C_1″_), 101.8 (C_5_), 88.7 (C_1′_), 86.1 (C_2″_), 85.0 (C_4″_), 84.6 (C_4′_), 81.7 (C_3″_), 78.2 (C_5′_), 75.5 (C_2′_), 72.0 (C_3′_), 53.4 (C_5″_), 31.9, 29.6, 29.4, 29.3, 29.2, 29.1, 28.9, 25.8 (C_2*_-C_9*_), 26.6, 26.5 (C_6′_, C_1*_), 25.76 (SiCCH_3_), 22.69 (s), 18.0 (SiCCH_3_), 14.1 (C_10*_), 8.4, 7.5 (C_8″_), –4.2, –4.5, –4.8 (SiCH_3_); HRMS ESI+ calcd. for C_44_H_78_N_7_O_10_Si_2_^+^ (M + H)^+^ 920.5343 found 920.5326.

#### 3.1.5. Protected Oxadiazole **11b**

Compound **11b** was obtained as a white soli (53 mg, 55% yield): R*_f_* 0.25 (cyclohexane/EtOAc = 7/3); [α]_D_ +10 (c 1.0, CH_2_Cl_2_); IR (film): 3006, 2873, 1558, 1427, 1368, 1275, 1146, 1016, 988, 935, 887, 820, 764, 750; ^1^H NMR (500 MHz, CDCl_3_) *δ* 8.61 (s, 1H, N*H*), 7.88 (d, *J*_H5-H6_ = 8.1Hz, 1H, H_6_), 5.84 (d, *J*_H1′-H2′_ = 4.3Hz, 1H, H_1′_), 5.72 (d, *J*_H5-H6_ = 8.1Hz, 1H, H_5_), 5.24 (s, 1H, H_1″_), 4.63 (d, *J*_H2″-H3″_ = 6.1Hz, 1H, H_3″_), 4.52 (d, *J*_H2″-H3″_ = 6.0 Hz, 1H, H_2″_), 4.33–4.29 (m, 1H, H_4″_), 4.26–4.19 (m, 1H, H_5′_), 4.18–4.14 (m, 1H, H_2′_), 4.05 (d, *J*_H3′-H4′_ = 3.0 Hz, 1H, H_4′_), 4.00 (d, *J*_H3′-H4′_ = 3.0 Hz, 1H, H_3′_), 3.50 (dd, *J*_H5″a-H5″b_ = 12.9 Hz, *J*_H4″-H5″a_ = 5.1Hz, 1H, H_5″a_), 3.42 (dd, *J*_H5″a-H5″b_ = 12.9 Hz, *J*_H4″-H5″b_ = 5.8 Hz, 1H, H_5″b_), 3.37 (dd, *J*_H6′a-H6′b_ = 14.9 Hz, *J*_H6′a-H5′_ = 5.0 Hz, 1H, H_6′a_), 3.19 (dd, *J*_H6′a-H6′b_ = 14.9 Hz, *J*_H6′b-H5′_ = 9.2 Hz, 1H, H_6′b_), 2.82 (t, *J*_H1*-H2*_ = 7.6 Hz, 2H, H_1*_), 1.84–1.62 (m, 4H, H_2*_, H_7″_), 1.56 (q, *J*_H7″-H8″_ = 7.2 Hz, 2H, H_7″_), 1.40–1.17 (m, 16H, H_2*_-H_9*_), 0.97–0.75 (m, 21H, H_10*_, H_8″_, SiC(CH_3_)_3_), 0.07, 0.07, 0.06 (s, 12H, SiCH_3_); ^13^C NMR (125 MHz, CDCl_3_) *δ* 180.3 (C_8′_), 166.9 (C_7′_), 162.9 (C_4_), 150.0 (C_2_), 140.3 (C_6_), 118.1 (C_6″_), 112.3 (C_1″_), 101.8 (C_5_), 88.8 (C_1′_), 86.1 (C_2″_), 85.0 (C_4″_), 84.7 (C_4′_), 81.7 (C_3”_), 78.3 (C_5′_), 75.5 (C_2′_), 72.1 (C_3′_), 53.4 (C_6′ab_), 32.0 (C_4*_), 29.8, 29.7, 29.5(C_5”b_), 29.3, 29.3, 29.2 (C_5”a_), 29.1 (C_7”_), 28.9 (C_7”_), 26.7 (C_1*_), 26.6 (C_2*_), 25.9 (SiC(CH_3_)_3_), 25.8 (SiC(CH_3_)_3_), 22.8 (C_3*_), 18.0 (C_14*_), 14.2 (C_15*_), 8.5 (C_8″_), 7.6 (C_8″_), −4.0 (SiCH_3_), −4.4 (SiCH_3_), −4.6 (SiC), −4.7 (SiC). HRMS ESI+ calcd. for C_49_H_88_N_7_O_10_Si_2_^+^ (M + H)^+^ 990.6125 found 990.6105.

#### 3.1.6. Protected Oxadiazole **11c**

Compound **11c** was obtained as a colorless oil (50 mg, 75% yield): R*_f_* 0.25 (cyclohexane/EtOAc = 7/3); [α]_D_ +16 (c 1.0, CH_2_Cl_2_); IR (film): 2926, 2855, 2105, 1695, 1462, 1378, 1275, 1167, 1100, 925, 867, 839, 764, 750; ^1^H NMR (500 MHz, CDCl_3_) *δ* 7.89 (d, *J*_H6-H5_ = 8.0 Hz, 1H, H_6_), 5.85 (d, *J*_H1′-H2′_ = 4.4 Hz, 1H, H_1′_), 5.73 (dd, *J*_H5-H6_ = 8.0 Hz, J_H5-H1′_ = 2.2 Hz, 1H, H_5_), 5.24 (s, 1H, H_1″_), 4.64 (dd, *J*_H3″-H2″_ = 6.6 Hz, *J*_H3″-H4″_ = 1.4 Hz, 1H, H_3″_), 4.51 (d, *J*_H2″-H3″_ = 6.5 Hz, 1H, H_2″_), 4.32 (t, *J*_H5′-H4′_ = 5.7 Hz, 1H, H_5′_), 4.24–4.21 (m, 1H, H_4″_), 4.16 (t, *J*_H2′-H3′_ = 4.4 Hz, 1H, H_2′_), 4.05 (dd, *J*_H4′-H3′_ = 4.2 Hz, *J*_H4′-H5′_ = 1.5 Hz, 1H, H_4′_), 3.98 (t, *J*_H3′-H4′_ = 4.2 Hz, 1H, H_3′_), 3.50 (dd, *J*_H6′a-H5′_ = 12.8 Hz, *J*_H6′a-H6′b_ = 5.8 Hz, 1H, H_6′a_), 3.43 (dd, *J*_H6′b-H5′_ = 12.8 Hz, *J*_H6′b-H5′_ = 5.8 Hz, 1H, H_6′b_), 3.38 (dd, *J*_H5″a-H4″_ = 15.0 Hz, *J*_H5″a-H5″b_ = 9.3 Hz, 1H, H_5″a_), 3.19 (dd, *J*_H5″b-H4″_ = 15.0 Hz, *J*_H5″b-H5″a_ = 9.3 Hz, 1H, H_5″b_), 2.83 (t, *J*_H2′-H3′_ = 7.6 Hz, 1H, H_1*_), 1.79–1.73 (m, 2H, H_2*_), 1.69 (q, *J*_H7″-H8″_ = 7.4 Hz, 2H, H_7″_), 1.56 (q, *J*_H7″-H8″_ = 7.4 Hz, 2 H, H_7″_), 1.38–1.24 ( m, 30H, H_3*_-H_17*_), 0.92–0.84 (m, 27H, SiC(CH_3_)_3_, H_8″_, H_18*_), 0.07–0.05 (12 H, Si(CH_3_)_2_); ^13^C NMR (125 MHz, CDCl_3_) δ 180.1 (C_8′_), 166.8 (C_7′_), 162.8 (C_4_), 149.9 (C_2_), 140.1 (C_6_), 117.9 (C_6″_), 112.1 (C_1″_), 101.6 (C_5_), 88.6 (C_1′_), 85.9 (C_2″_), 84.9 (C_4″_), 84.5 (C_4′_), 81.6 (C_3”_), 78.1 (C_5′_), 75.4 (C_2′_), 71.9 (C_3′_), 53.2 (C_6′ab_), 31.9 (C_4*_), 29.6, 29.3, 29.3(C_5”b_), 29.2, 29.1, 29.1 (C_5”a_), 29.0 (C_7”_), 28.7 (C_7”_), 26.6 (C_1*_), 26.4 (C_2*_), 25.7 (SiC(CH_3_)_3_), 25.6 (SiC(CH_3_)_3_), 22.6 (C_3*_), 17.9 (C_17*_), 14.1 (C_18*_), 8.3 (C_8″_), 7.4 (C_8″_), −4.2 (SiCH_3_), −4.5 (SiCH_3_), −4.8 (SiC), −4.8 (SiC); HRMS ESI+ calcd. for C_52_H_94_N_7_O_10_Si_2_^+^ (M + H)^+^ 1032.6595 found 1032.6576.

#### 3.1.7. Protected Oxadiazole **11d**

Compound **11d** was obtained as a colorless oil (47.5 mg, 72% yield): R*_f_* 0.25 (cyclohexane/EtOAc = 7/3); [α]_D_ +33 (c 1.0, CH_2_Cl_2_); IR (film): 2928, 2856, 2105, 1695, 1462, 1378, 1275, 1167, 1099, 924, 903, 867, 838, 764, 750; ^1^H NMR (500 MHz, CDCl_3_) *δ* 7.89 (d, *J*_H6-H5_ = 8.2 Hz, 1H, H_6_), 5.85 (d, *J*_H1′-H2′_ = 4.7 Hz, 1H, H_1′_), 5.73 (d, *J*_H5-H6_ = 8.2 Hz, 1H, H_5_), 5.35–5.30 (m, 2 H, H_8*_, H_9*_), 5.24 (s, 1H, H_1″_), 4.64 (dd, *J*_H3″-H2″_ = 6.2 Hz, *J*_H3″-H4″_ = 1.5 Hz, 1H, H_3″_), 4.52 (d, *J*_H2″-H3″_ = 6.5 Hz, 1H, H_2″_), 4.31 (t, *J*_H5′-H4′_ = 5.8 Hz, 1H, H_5′_), 4.24–4.21 (m, 1H, H_4″_), 4.15 (t, *J*_H2′-H3′_ = 4.5 Hz, 1H, H_2′_), 4.05 (dd, *J*_H4′-H3′_ = 4.2 Hz, *J*_H4′-H5′_ = 1.6 Hz, 1H, H_4′_), 3.98 (t, *J*_H3′-H4′_ = 4.3 Hz, 1H, H_3′_), 3.50 (dd, *J*_H6′a-H5′_ = 13.0 Hz, *J*_H6′a-H6′b_ = 5.2 Hz, 1H, H_6′a_), 3.43 (dd, *J*_H6′b-H5′_ = 13.0 Hz, *J*_H6′b-H5′_ = 5.8 Hz, 1H, H_6′b_), 3.38 (dd, *J*_H5″a-H4″_ = 15.0 Hz, *J*_H5″a-H5″b_ = 9.2 Hz, 1H, H_5″a_), 3.19 (dd, *J*_H5″b-H4″_ = 15.0 Hz, *J*_H5″b-H5″a_ = 9.2 Hz, 1H, H_5″b_), 2.84 (t, *J*_H2′-H3′_ = 7.8 Hz, 1H, H_1*_), 2.02–1.98 (m, 4 H, H_7*_, H_10*_), 1.80–1.74 (m, 2 H, H_2*_), 1.69 (q, *J*_H7″-H8″_ = 7.4 Hz, 2 H, H_7″_), 1.56 (q, *J*_H7″-H8″_ = 7.4 Hz, 2 H, H_7″_), 1.42 –1.24 (m, 20 H, H_3*_-H_6*_, H_9*_-H_16*_), 0.92–0.84 ( m, 27H, -SiC(CH_3_)_3_, H_8″_, H_17*_), 0.07–0.05 (12 H, Si(CH_3_)_2_); ^13^C NMR (125 MHz, CDCl_3_) *δ* 180.2 (C_8′_), 166.9 (C_7′_), 163.0 (C_4_), 150.0 (C_2_), 140.2 (C_6_), 130.2 (C_8*_), 129.7 (C_9*_), 118.0 (C_6″_), 112.3 (C_1″_), 101.8 (C_5_), 88.8 (C_1′_), 86.1 (C_2″_), 85.0 (C_4″_), 84.7 (C_4′_), 81.7 (C_3”_), 78.3 (C_5′_), 75.5 (C_2′_), 72.0 (C_3′_), 53.4 (C_6′ab_), 32.0 (C_4*_), 29.9, 29.8(C_5”b_), 29.6, 29.4, 29.3 (C_5”a_), 29.1 (C_7”_), 28.9 (C_7”_), 27.3 (C_7*_), 27.2 (C_10*_)_,_ 26.7 (C_1*_), 26.6 (C_2*_), 25.9 (SiC(CH_3_)_3_), 25.8 (SiC(CH_3_)_3_), 22.8 (C_3*_), 18.1 (C_16*_ ), 14.2 (C_17*_), 8.5 (C_8″_), 7.6 (C_8″_), −4.0 (SiCH_3_), −4.4 (SiCH_3_), −4.6 (SiC), −4.7 (SiC); HRMS ESI+ calcd. for C_51_H_90_N_7_O_10_Si_2_^+^ (M + H)^+^ 1016.6282 found 1016.6258.

#### 3.1.8. Protected Oxadiazole **11e**

Compound **11e** was obtained as a colorless oil (23 mg, 42% yield): R*_f_* 0.25 (cyclohexane/EtOAc = 7/3); [α]_D_ +33 (c 1.0, CH_2_Cl_2_); IR (film): 2930, 2106, 1697, 1487, 1368, 1275, 1275, 1168, 1100, 924, 870, 839, 764, 750; ^1^H NMR (500 MHz, CDCl_3_) *δ* 8.06 (d, *J*_Ha-Hb_ = 8.8 Hz, 2 H, H_a_), 7.91 (dd, *J*_H6-H5_ = 8.3 Hz, J_H6-H1′_ = 3.3 Hz, 1H, H_6_), 7.41 (t, *J*_Hd-He_ = 7.9 Hz, 2 H, H_d_), 7.22 (t, *J*_He-Hd_ = 7.4 Hz, 1H, H_e_), 7.09 (d, *J*_Hd-Hc_ = 8.8 Hz, 2 H, H_c_), 7.07 (d, *J*_Hb-Ha_ = 8.8 Hz, 2 H, H_b_), 5.85 (d, *J*_H1′-H2′_ = 4.4 Hz, 1H, H_1′_), 5.74 (dd, *J*_H5-H6_ = 8.3 Hz, *J*_H5-H1′_ = 2.0 Hz, 1H, H_5_), 5.29 (s, 1H, H_1″_), 4.65 (dd, *J*_H3″-H2″_ = 6.2 Hz, *J*_H3″-H4″_ = 1.5 Hz, 1H, H_3″_), 4.54 (d, *J*_H2″-H3″_ = 6.2 Hz, 1H, H_2″_), 4.32–4.21 (m, 2 H, H_5′_, H_4″_), 4.18–4.14 (m, 2 H, H_2′_, H_4′_), 4.01 (t, *J*_H3′-H4′_ = 4.2 Hz, 1H, H_3′_), 3.52 (dd, *J*_H6′a-H5′_ = 12.8 Hz, *J*_H6′a-H6′b_ = 5.5 Hz, 1H, H_6′a_), 3.47–3.42 (m, 2 H, H_6′_b, H_5″a_), 3.27 (dd, *J*_H5″b-H4″_ = 15.0 Hz, *J*_H5″b-H5″a_ = 9.2 Hz, 1H, H_5″b_), 1.70 (q, *J*_H7″-H8″_ = 7.4 Hz, 2 H, H_7″_), 1.56 (q, *J*_H7″-H8″_ = 7.4 Hz, 2 H, H_7″_), 0.92–0.82 (m, 24H, -SiC(CH_3_)_3_, H_8″_), 0.07–0.05 (12 H, Si(CH_3_)_2_); ^13^C NMR (125 MHz, CDCl_3_) *δ* 175.4 (C_8′_), 167.6 (C_7′_), 161.9 (C_4_), 155.4 (C_10′_), 150.0 (C_2_), 130.2 (C_a_, C_d_), 124.9 (C_e_), 120.3 (C_c_), 118.3 (C_b_), 118.1 (C_6″_), 112.2 (C_1″_), 101.8 (C_5_), 88.8 (C_1′_), 86.1 (C_2″_), 85.0 (C_4″_), 84.8 (C_4′_), 81.7 (C_3”_), 78.2 (C_5′_), 75.5 (C_2′_), 72.1 (C_3′_), 53.4 (C_6′ab_), 32.0 (C_4*_), 29.9(C_5”ab_), 29.3 (C_7”_), 28.9 (C_7”_), 25.8 (SiC(CH_3_)_3_), 25.8 (SiC(CH_3_)_3_, 18.1, 18.0 (C_9′_), 8.5 (C_8″_), 7.6 (C_8″_), −4.0 (SiCH_3_), −4.4 (SiCH_3_), −4.6 (SiC), −4.7 (SiC). HRMS ESI+ calcd. for C_46_H_66_N_7_O_11_Si_2_^+^ (M + H)^+^ 948.4353 found 948.4329.

#### 3.1.9. Protected Oxadiazole **11f**

Compound **11f** was obtained as a colorless oil (55 mg, 83% yield): R*_f_* 0.25 (cyclohexane/EtOAc = 7/3); [α]_D_ +15 (c 1.0, CH_2_Cl_2_); IR (film): 2928, 2856, 2105, 1696, 1580, 1462, 1378, 1274, 1167, 1099, 967, 910, 867, 838, 764, 748; ^1^H NMR (500 MHz, CDCl_3_) *δ* 7.89 (d, *J*_H6-H5_ = 8.2 Hz, 1H, H_6_), 5.84 (d, *J*_H1′-H2′_ = 4.4 Hz, 1H, H_1′_), 5.73 (dd, *J*_H5-H6_ = 8.2 Hz, *J*_H5-H1′_ = 2.0 Hz, 1H, H_5_), 5.42–5.33 (m, 2 H, H_8*_, H_9*_), 5.24 (s, 1H, H_1″_), 4.63 (dd, *J*_H3″-H2″_ = 6.2 Hz, *J*_H3″-H4″_ = 1.6 Hz, 1H, H_3″_), 4.51 (d, *J*_H2″-H3″_ = 6.0 Hz, 1H, H_2″_), 4.31 (t, *J*_H5′-H4′_ = 5.7 Hz, 1H, H_5′_), 4.24–4.20 (m, 1H, H_4″_), 4.15 (t, *J*_H2′-H3′_ = 4.5 Hz, 1H, H_2′_), 4.05 (dd, *J*_H4′-H3′_ = 4.1Hz, *J*_H4′-H5′_ = 1.7 Hz, 1H, H_4′_), 3.98 (t, *J*_H3′-H4′_ = 4.4 Hz, 1H, H_3′_), 3.50 (dd, *J*_H6′a-H5′_ = 12.6 Hz, *J*_H6′a-H6′b_ = 5.1Hz, 1H, H_6′a_), 3.43 (dd, *J*_H6′b-H5′_ = 12.6 Hz, *J*_H6′b-H5′_ = 6.1Hz, 1H, H_6′b_), 3.38 (dd, *J*_H5″a-H4″_ = 14.7 Hz, *J*_H5″a-H5″b_ = 9.0 Hz, 1H, H_5″a_), 3.19 (dd, *J*_H5″b-H4″_ = 15.0 Hz, *J*_H5″b-H5″a_ = 9.0 Hz, 1H, H_5″b_), 2.83 (t, *J*_H2′-H3′_ = 7.7 Hz, 1H, H_1*_), 1.97–1.94 (m, 4 H, H_7*_, H_10*_), 1.80–1.73 (m, 2 H, H_2*_), 1.70 (q, *J*_H7″-H8″_ = 7.6 Hz, 2 H, H_7″_), 1.56 (q, *J*_H7″-H8″_ = 7.6 Hz, 2 H, H_7″_), 1.32–1.25 ( m, 20 H, H_3*_-H_6*_, H_9*_-H_16*_), 0.92–0.84 (m, 27H, -SiC(CH_3_)_3_, H_8″_, H_17*_), 0.07–0.05 (12 H, Si(CH_3_)_2_). ^13^C NMR (125 MHz, CDCl_3_) *δ* 180.2 (C_8′_), 166.9 (C_7′_), 162.9 (C_4_), 150.0 (C_2_), 140.2 (C_6_), 130.6 (C_8*_), 130.2 (C_9*_), 118.0 (C_6″_), 112.3 (C_1″_), 101.8 (C_5_), 88.8 (C_1′_), 86.1 (C_2″_), 85.0 (C_4″_), 84.7 (C_4′_), 81.7 (C_3”_), 78.3 (C_5′_), 75.5 (C_2′_), 72.0 (C_3′_), 53.4 (C_6′ab_), 32.7 (C_7*_), 32.6 (C_10*_), 32.0 (C_4*_), 29.7, 29.6 (C_5”b_), 29.5, 29.4, 29.3 (C_5”a_), 29.1 (C_7”_), 28.9 (C_7”_), 26.7 (C_1*_), 26.6 (C_2*_), 25.9 (SiC(CH_3_)_3_), 25.8 (SiC(CH_3_)_3_), 22.8 (C_3*_), 18.1 (C_16*_), 14.2 (C_17*_), 8.5 (C_8″_), 7.6 (C_8″_), −4.0 (SiCH_3_), −4.4 (SiCH_3_), −4.6 (SiC), −4.7 (SiC); HRMS ESI+ calcd. for C_51_H_90_N_7_O_10_Si_2_^+^ (M + H)^+^ 1016.6282 found 1016.6274.

#### 3.1.10. Protected Oxadiazole **11g**

Compound **11g** was obtained as a colorless oil (38 mg, 58% yield): R*_f_* 0.25 (cyclohexane/EtOAc = 7/3); [α]_D_ +6 (c 1.0, CH_2_Cl_2_); IR (film): 2929, 2856, 2105, 1695, 1580, 1462, 1377, 1274, 1167, 1100, 924, 865, 839, 764, 750; ^1^H NMR (500 MHz, CDCl_3_) *δ* 7.89 (d, *J*_H6-H5_ = 8.1Hz, 1H, H_6_), 7.28–7.16 (m, 5 H, H_abc_), 5.85 (d, *J*_H1′-H2′_ = 4.7 Hz, 1H, H_1′_), 5.73 (dd, *J*_H5-H6_ = 8.1Hz, J_H5-H1′_ = 2.0 Hz, 1H, H_5_), 5.24 (s, 1H, H_1″_), 4.64 (dd, *J*_H3″-H2″_ = 6.1Hz, *J*_H3″-H4″_ = 1.4 Hz, 1H, H_3″_), 4.52 (d, *J*_H2″-H3″_ = 6.1Hz, 1H, H_2″_), 4.32 (t, *J*_H5′-H4′_ = 5.7 Hz, 1H, H_5′_), 4.24–4.21 (m, 1H, H_4″_), 4.16 (t, *J*_H2′-H3′_ = 4.4 Hz, 1H, H_2′_), 4.05 (dd, *J*_H4′-H3′_ = 4.3 Hz, *J*_H4′-H5′_ = 1.7 Hz, 1H, H_4′_), 3.98 (t, *J*_H3′-H4′_ = 4.5 Hz, 1H, H_3′_), 3.50 (dd, *J*_H6′a-H5′_ = 12.8 Hz, *J*_H6′a-H6′b_ = 5.8 Hz, 1H, H_6′a_), 3.43 (dd, *J*_H6′b-H5′_ = 12.8 Hz, *J*_H6′b-H5′_ = 5.8 Hz, 1H, H_6′b_), 3.38 (dd, *J*_H5″a-H4″_ = 15.0 Hz, *J*_H5″a-H5″b_ = 9.3 Hz, 1H, H_5″a_), 3.19 (dd, *J*_H5″b-H4″_ = 15.0 Hz, *J*_H5″b-H5″a_ = 9.3 Hz, 1H, H_5″b_), 2.82 (t, *J*_H2′-H3′_ = 7.6 Hz, 1H, H_1*_), 2.59 (t, *J*_H9′-H10′_ = 7.6 Hz, 1H, H_9*_), 1.79–1.73 (m, 2H, H_2*_), 1.70 (q, *J*_H7″-H8″_ = 7.6 Hz, 2 H, H_7″_), 1.63–1.58 (m, 2H, H_8*_), 1.56 (q, *J*_H7″-H8″_ = 7.6 Hz, 2H, H_7″_), 1.36–1.26 ( m, 10 H, H_3*_-H_7*_), 0.92–0.84 ( m, 24H, -SiC(CH_3_)_3_, H_8″_, H_18*_), 0.07–0.05 (12 H, Si(CH_3_)_2_); ^13^C NMR (125 MHz, CDCl_3_) *δ* 180.2 (C_8′_), 166.9 (C_7′_), 162.9 (C_4_), 150.0 (C_2_), 142.9 (C_10*_), 140.2 (C_6_), 128.5 (C_a_), 128.3 (C_b_), 125.7 (C_c_), 118.0 (C_6″_), 112.3 (C_1″_), 101.8 (C_5_), 88.8 (C_1′_), 86.1 (C_2″_), 85.0 (C_4″_), 84.7 (C_4′_), 81.7 (C_3”_), 78.3 (C_5′_), 75.5 (C_2′_), 72.0 (C_3′_), 53.4 (C_6′ab_), 36.1 (C_9*_), 31.6 (C_4*_), 29.5, 29.4, 29.4 (C_5”b_), 29.3, 29.3, 29.2 (C_5”a_), 29.1 (C_7”_), 28.9 (C_7”_), 26.7 (C_1*_), 26.6 (C_2*_), 25.9 (SiC(CH_3_)_3_), 25.8 (SiC(CH_3_)_3_), 17.9 (C_8*_), 8.5 (C_8″_), 7.6 (C_8″_), −4.0 (SiCH_3_), −4.4 (SiCH_3_), −4.6 (SiC), −4.7 (SiC). HRMS ESI− calcd. for C_49_H_78_N_7_O_10_Si_2_^−^ (M + H)^−^ 980.5354 found 980.5297.

#### 3.1.11. Protected Oxadiazole **11h**

Compound **11h** was obtained as a colorless oil and as a 1/1 mixture of stereoisomers (38 mg, 58% yield): R*_f_* 0.25 (cyclohexane/EtOAc = 7/3); [α]_D_ +6 (c 1.0, CH_2_Cl_2_); IR (film): 2929, 2856, 2105, 1695, 1580, 1462, 1377, 1274, 1167, 1100, 924, 865, 839, 764, 750; ^1^H NMR (500 MHz, CDCl_3_) *δ* 7.90 (d, *J*_H5-H6_ = 8.1 Hz, 1H, H_6_), 5.85 (d, *J*_H1′-H2′_ = 3.1 Hz, 0.5H, H_1′_), 5.84 (d, *J*_H1′-H2′_ = 3.4 Hz, 0.5H, H_1′_), 5.74 (d, *J*_H5-H6_ = 8.1 Hz, 1H, H_5_), 5.22 (s, 0.5H, H_1″_), 5.22 (s, 0.5 H, H_1″_), 4.63 (d, *J*_H2″-H3″_ = 6.1 Hz, 1H, H_3″_), 4.51 (d, *J*_H2″-H3″_ = 6.1 Hz, 1H, H_2″_), 4.35–4.23 (m, 2H, H_4″_, H_5′_), 4.23–4.14 (m, 3 H, H_3**_, H_2′_), 4.08 (dd, *J*_H4′-H5′_ = 8.5 Hz, *J*_H3′-H4′_ = 4.4 Hz, 0.5H, H_4′_), 4.07 (dd, *J*_H4′-H5′_ = 8.5 Hz, *J*_H3′-H4′_ = 4.4 Hz, 0.5 H, H_4′_), 4.00 (t, *J*_H3′-H4′_ = *J*_H2′-H3′_ = 4.4 Hz, 1H, H_3′_), 3.95 (t, *J* = 7.6 Hz, 1H, H_1*_), 3.50 (dd, *J*_H5″a-H5″b_ = 12.7, *J*_H4″-H5″a_ = 4.9 Hz, 0.5H, H_5″a_), 3.49 (dd, *J*_H5″a-H5″b_ = 12.7, *J*_H4″-H5″a_ = 4.9 Hz, 0.5H, H_5″a_), 3.46–3.34 (m, 2 H, H6′a, H_5″b_), 3.26–3.16 (m, 1H, H_6′b_), 2.18–1.96 (m, 2 H, H_2*_), 1.75–1.65 (m, 2H, H_7″_), 1.59–1.51 (m, 2H, H_7″_), 1.40–1.5 (m, 17H, H_3*_-H_9*_, H_4**_), 0.94–0.78 (m, 21 H, H_10*_, H_8″_, SiC(CH_3_)_3_), 0.07, 0.07, 0.06 (s, 12H, SiCH_3_); ^13^C NMR (125 MHz, CDCl_3_) *δ* 176.74, 176.67 (C_2**_), 168.66, 168.63 (C_8′_), 167.23, 167.20 (C_7′_), 162.9, 162.8 (C_4_), 150.00, 149.95 (C_2_), 140.2 (C_6_), 117.96, 117.93 (C_6″_), 112.34, 112.29 (C_1″_), 101.76 (C_5_), 88.81 (C_1′_), 86.03 (C_2″_), 84.98, 84.86 (C_4′_, C_4″_), 81.7 (C_3″_), 78.2, 78.1 (C_5′_), 75.5 (C_2′_), 71.9 (C_3′_), 62.02 (C_3**_), 53.27, 53.25 (C_5″_), 44.60, 44.55 (C_1*_), 31.89 (C_2*_), 30.2, 29.7, 29.5, 29.4, 29.3, 29.2, 29.1, 28.8, 27.20, 22.7 (C_3*_-C_9*_, C_7″_), 25.81, 25.76 (SiCCH_3_), 18.0 (SiCCH_3_), 14.15, 14.12 (C_4**_), 8.4, 7.5 (C_8″_), −4.2, −4.5, −4.8 (SiCH_3_); HRMS ESI− calcd. for C_47_H_80_N_7_O_12_Si_2_^−^ (M + H)^−^ 990,5409 found 990.5455.

#### 3.1.12. Protected Oxadiazole **11i**

Compound **11i** was obtained as a colorless oil (57 mg, 74% yield): R*_f_* 0.25 (cyclohexane/EtOAc = 7/3); [α]_D_ +10 (c 1.0, CH_2_Cl_2_); IR (film): 2928, 2856, 1697, 1463, 1260, 1168, 1102, 840, 779, 740; ^1^H NMR (500 MHz, CDCl_3_) *δ* 7.93 (d, *J*_H6-H5_ = 8.5 Hz, 1H, H_6_), 5.83 (d, *J*_H1′-H2′_ = 4.3 Hz, 1H, H_1′_), 5.73 (dd, *J*_H5-H6_ = 8.3 Hz, *J*_H5-H1′_ = 2.2 Hz, 1H, H_5_), 5.23 (s, 1H, H_1″_), 4.63 (dd, *J*_H3″-H2″_ = 6.3 Hz, *J*_H3″-H4″_ = 1.2 Hz, 1H, H_3″_), 4.51 (d, *J*_H2″-H3″_ = 6.4 Hz, 1H, H_2″_), 4.31-4.24 (m, 2 H, H_5′_,H_4″_), 4.16 (d, *J*_H2′-H3′_ = 3.9 Hz, 1H, H_2′_), 4.17 (q, *J*_H3***-H4***_ = 7.1Hz, 1H, H_3***_), 4.09 (dd, *J*_H4′-H3′_ = 4.9 Hz, J_H4′-H5′_ = 1.4 Hz, 1H, H_4′_), 4.01 (t, *J*_H3′-H4′_ = 4.6 Hz, 1H, H_3′_), 3.52 (dd, *J*_H5″a-H5″b_ = 13 Hz, *J*_H5″a-H6′_ = 4.9 Hz, 1H, H_5″a_), 3.43 (dd, *J*_H5″b-H5″a_ = 13 Hz, *J*_H5″b-H4″_ = 5.8 Hz, 1H, H_5″b_), 3.40 (dd, *J*_H6″a-H6″b_ = 15.4 Hz, *J*_H6″a-H5′_ = 5.5 Hz, 1H, H_6″a_), 3.21 (dd, *J*_H6″b-H6″a_ = 15.4 Hz, *J*_H6″b-H5″_ = 8.4 Hz, 1H, H_6″b_), 2.12-2.00 (m, 4 H, H_2*_,H_2**_), 1.68 (q, *J*_H7″a-H8″_ = 7.4 Hz, 2 H, H_7″a_), 1.55 (q, *J*_H7″b-H8″_ = 7.4 Hz, 2 H, H_7″b_), 1.28–1.19 ( m, 33 H, H_3**_-H_10**_, H_4*_-H_10*_, H_4***_), 1.14–1.03 ( m, 3H, H_3*_), 0.91–0.83 ( m, 30H, -SiC(CH_3_)_3_, H_8″_, H_11*_, H_11**_), 0.09–0.07 (12 H, Si(CH_3_)_2_). ^13^C NMR (125 MHz, CDCl_3_) *δ* 183.4 (C_8′_), 166.8 (C_7′_), 163.0 (C_4_), 150.0 (C_2_), 140.3 (C_6_), 118.0 (C_6″_), 112.3 (C_1″_), 101.7 (C_5_), 89.0 (C_1′_), 86.1 (C_2″_), 85.1 (C_4″_), 84.7 (C_4′_), 81.7 (C_3”_), 78.3 (C_5′_), 75.6 (C_2′_), 71.8 (C_3′_), 53.4 (C_5”_), 38.5 (C_1*_), 33.5 (C_2*_), 33.4 (C_2**_), 32.0, 29.7, 29.7, 29.5, 29.4, 29.3, 28.9 (C_7”_), 27.3 (C_7”_), 26.0 (SiC(CH_3_)_3_), 25.9 (SiC(CH_3_)_3_), 22.8 (C_3*_), 18.0 (C_3**_ ), 14.2 (C_4***_,C_11**_,C_11*_), 8.5 (C_8″_), 7.6 (C_8″_), −4.0 (SiCH_3_), −4.3(SiCH_3_), −4.6(SiC), −4.7(SiC). HRMS ESI+ calcd. for C_55_H_100_N_7_O_10_Si_2_^+^ (M + H)^+^ 1074.7064 found 1074.7060.

#### 3.1.13. General Procedure for Compounds Deprotection

The deprotection of compounds **11a**–**11i** was performed according to reference [42] and afforded the fully deprotected compounds **12a**–**12i** in 22 to 85% yield over two steps.

#### 3.1.14. Oxadiazole **12a**

Compound **12a** was obtained from the protected oxadiazole **11a** (30 mg, 32 μmol, 1 equiv.) as a white solid (16.7 mg, 85% yield): R*_f_* 0.15 (DCM/MeOH/NH_4_OH 14% 80/18/2); [α]_D_ + 15 (c 1.0, CH_2_Cl_2_); IR (film): 2912, 2888, 2240, 1750, 1700, 1650, 1400, 1275, 1260, 1000, 764, 750.; ^1^H NMR (500 MHz, MeOD) *δ* 7.80 (d, *J*_H6-H5_ = 8.1Hz, 1H, H_6_), 5.77 (d, *J*_H1′-H2′_ = 3.1Hz, 1H, H_1′_), 5.73 (d, *J*_H5-H6_ = 8.1Hz, 1H, H_5_), 5.14 (s, 1H, H_1″_), 4.33–4.27 (m, 1H, H_2′_), 4.18–4.12 (m, 2 H, H_3′_, H_4′_), 4.09 (t, *J*_H4″-H5″_ = 7.1Hz, 1H, H_4″_), 4.04 (m, 2 H, H_2″_, H_3″_), 3.98 (m, 1H, H_5′_), 3.37–3.32 (m, 1H, H_6′a_) 3.29–3.26 (m,1H, H_5″a_), 3.20 (dd, *J*_H6′b-H6′a_ = 15.0, *J*_H6′b-H5′_ = 5.7 Hz, 1H, H_6′b_), 3.11 (dd, *J*_H5″b-H5″a_ = 13.0, *J*_H5″b-H4″_ = 7.1Hz,1H, H_5″b_), 2.91 (t, *J*_H2*-H3*_ = 7.5 Hz, 2 H, H_2*_), 1.79 (dt, *J*_H3*-H2*_ = *J*_H3*-H4*_ = 7.4 Hz, 2 H, H_3*_), 1.44–1.20 (m,14 H), 0.89 (t, *J*_H11*-H10*_ = 6.8 Hz, 3 H, H_11*_).; ^13^C NMR (125 MHz, MeOD) *δ* 181.9 (C_1*_), 168.7 (C_7′_), 166.1(C_4_), 152.1 (C_2_), 142.2 (C_6_), 111.0 (C_1″_), 102.5 (C_5_), 91.6 (C_1′_), 85.7 (C_3′_), 80.0 (C_4″_), 78.1 (C_5′_), 76.3 (C_3″_), 75.5 (C_2′_), 73.8 (C_2″_), 71.3 (C_4′_), 44.3 (C_5″_), 33.0, 30.6 (C_6′_), 30.39, 30.20, 30.02, 27.6 (C_3*_), 27.1 (C_2*_),23.7, 14.4; HRMS ESI+ calcd. for C_27_H_43_N_5_O_10_ (M + H)^+^ 598.3083, found 598.3082.

#### 3.1.15. Oxadiazole **12b**

Compound **12b** was obtained from the protected oxadiazole **11b** (27 mg, 27 μmol, 1 equiv.) as a white solid (10.7 mg, 59% yield): R*_f_* 0.15 (DCM/MeOH/NH_4_OH 14% 80/18/2); [α]_D_ + 1 (c 1.0, CH_3_OH); IR (film): 2924, 2853, 1673, 1466, 1272, 1203, 1136, 800, 724; ^1^H NMR (500 MHz, MeOD) *δ* 7.81 (d, *J*_H6-H5_ = 8.1Hz, 1H, H_6_), 5.77 (d, *J*_H1′-H2′_ = 3.2 Hz, 1H, H_1′_), 5.72 (d, *J*_H5-H6_ = 8.1Hz, 1H, H_5_), 5.14 (s, 1H, H_1″_), 4.30 (td, *J*_H5′-H6′_ = 6.5 Hz, *J*_H5′-H4′_ = 2.5 Hz, 1H, H_5′_), 4.19-4.01 (m, 5 H, H_2′_, H_3′_, H_4′_, H_3″_, H_2″_), 3.98 (d, *J*_H4″-H5″_ = 4.3 Hz, 1H, H_4″_), 3.30–3.27 (m, 2 H, H_6′ab_), 3.21 (dd, *J*_H5″a-H4″_ = 14.9 Hz, *J*_H5″a-H5″b_ = 5.7 Hz, 1H, H_5″a_), 3.12 (dd, *J*_H5″b-H4″_ = 13.0 Hz, *J*_H5″b-H5″a_ = 9.0 Hz, 1H, H_5″b_), 2.91 (t, *J*_H1*-H2*_ = 7.5 Hz, 2H, H_1*_), 1.82–1.76 (m, 2H, H_2*_), 1.38–1.29 ( m, 24 H, H_3*_-H_14*_), 0.90 (t, *J*_H15*-H14*_ = 7.0 Hz, 3H, H_15*_); ^13^C NMR (125 MHz, MeOD) *δ* 181.1 (C_8′_), 168.7 (C_7′_), 166.1 (C_4_), 152.0 (C_2_), 142.2 (C_6_), 110.9 (C_1″_), 102.4 (C_5_), 91.5 (C_1′_), 85.6 (C_2″_), 79.9 (C_4′_), 78.0 (C_5′_), 76.2 (C_4”_), 75.5 (C_2′_), 73.8 (C_3″_), 71.2 (C3′), 44.3 (C_5”_), 33.0 (C_6′_), 30.8, 30.7, 30.7, 30.5, 30.4, 30.3, 30.2, 30.0 (C_4*_-C_14*_), 27.6 (C_2*_), 27.1 (C_1*_), 23.7 (C_3*_), 14.4 (C_15*_). HRMS ESI+ calcd. for C_32_H_54_N_5_O_10_^+^ (M + H)^+^ 668.3865 found 668.3860.

#### 3.1.16. Oxadiazole **12c**

Compound **12c** was obtained from the protected oxadiazole **11c** (28 mg, 27 μmol, 1 equiv.) as a colorless oil (9.2 mg, 40% yield): R*_f_* 0.15 (DCM/MeOH/NH_4_OH 14% 80/18/2); [α]_D_ + 0.8 (c 1.0, CH_3_OH); IR (film): 2921, 2851, 1672, 1432, 1275, 1204, 1138, 961, 800, 764.; ^1^H NMR (500 MHz, MeOD) *δ* 7.81 (d, *J*_H6-H5_ = 8.0 Hz, 1H, H_6_), 5.76 (d, J_H1′-H2′_ = 3.2 Hz, 1H, H_1′_), 5.72 (d, J_H5-H6_ = 8.0 Hz, 1H, H_5_), 5.14 (s, 1H, H_1″_), 4.30 (td, J_H5′-H6′_ = 6.5 Hz, J_H5′-H4′_ = 2.5 Hz, 1H, H_5′_), 4.17–4.01 (m, 5 H, H_2′_, H_3′_, H_4′_, H_3″_, H2″), 3.98 (d, J_H4″-H5″_ = 4.1Hz, 1H, H_4″_), 3.30–3.25 (m, 2 H, H_6′ab_), 3.21 (dd, J_H5″a-H4″_ = 15.0 Hz, J_H5″a-H5″b_ = 5.6 Hz, 1H, H_5″a_), 3.12 (dd, J_H5″b-H4″_ = 13.0 Hz, J_H5″b-H5″a_ = 9.0 Hz, 1H, _H5″b_), 2.91 (t, J_H1*-H2*_ = 7.5 Hz, 2 H, H_1*_), 1.80–1.76 (m, 2 H, H_2*_), 1.36–1.29 ( m, 30 H, H_3*_-H_17*_), 0.90 (t, J_H15*-H14*_ = 6.9 Hz, 3H, H_18*_); ^13^C NMR (125 MHz, MeOD) δ 181.8 (C_8′_), 168.7 (C_7′_), 166.1 (C_4_), 152.0 (C_2_), 142.2 (C_6_), 110.9 (C_1″_), 102.4 (C_5_), 91.6 (C_1′_), 85.6 (C_2″_), 79.9 (C_4′_), 78.1 (C_5′_), 76.2 (C_4”_), 75.5 (C_2′_), 73.8 (C_3″_), 71.2 (C_3′_), 44.3 (C_5”_), 33.0 (C_6′_), 30.8, 30.5, 30.4, 30.3, 30.2, 30.0 (C_4*_-C_17*_), 27.6 (C_2*_), 27.1 (C_1*_), 23.7 (C_3*_), 14.4 (C_18*_); HRMS ESI+ calcd. for C_35_H_60_N_5_O_10_^+^ (M + H)^+^ 710.4335 found 710.4325.

#### 3.1.17. Oxadiazole **12d**

Compound **12d** was obtained from the protected oxadiazole **11d** (22 mg, 21 μmol, 1 equiv.) as a colorless oil (12 mg, 66% yield): R*_f_* 0.15 (DCM/MeOH/NH_4_OH 14% 80/18/2); [α]_D_ + 0.9 (c 1.0, CH_3_OH); IR (film): 3007, 2920, 2850, 1670, 1435, 1275, 1204, 1138, 960, 801, 764, 750; ^1^H NMR (500 MHz, MeOD) *δ* 7.81 (d, *J*_H6-H5_ = 8.1Hz, 1H, H_6_), 5.76 (d, *J*_H1′-H2′_ = 3.3 Hz, 1H, H_1′_), 5.73 (d, *J*_H5-H6_ = 8.1Hz, 1H, H_5_), 5.34 (t, *J*_H8*-H9*_ = 4.6 Hz, 2 H, H_8*_, H_9*_), 5.14 (s, 1H, H_1″_), 4.30 (td, *J*_H5′-H6′_ = 6.5 Hz, *J*_H5′-H4′_ = 2.5 Hz, 1H, H_5′_), 4.15–4.01 (m, 5 H, H_2′_, H_3′_, H_4′_, H_3″_, H_2″_), 3.98 (d, *J*_H4″-H5″_ = 4.3 Hz, 1H, H_4″_), 3.29–3.25 (m, 2 H, H_6′ab_), 3.21 (dd, *J*_H5″a-H4″_ = 14.6 Hz, *J*_H5″a-H5″b_ = 5.8 Hz, 1H, H_5″a_), 3.12 (dd, *J*_H5″b-H4″_ = 12.3 Hz, *J*_H5″b-H5″a_ = 9.2 Hz, 1H, H_5″b_), 2.91 (t, *J*_H1*-H2*_ = 7.3 Hz, 2 H, H_1*_), 2.06–2.01 (m, 4 H, H_7*_, H1_0*_), 1.81–1.76 (m, 2 H, H_2*_), 1.35–1.29 ( m, 20 H, H_3*_-H_6*_, H_11*_-H_16*_), 0.91 (t, *J*_H15*-H14*_ = 6.7 Hz, 3H, H_17*_); ^13^C NMR (125 MHz, MeOD) δ 181.8 (C_8′_), 168.7 (C_7′_), 166.1 (C_4_), 152.0 (C_2_), 142.2 (C_6_), 130.9 (C_8*_), 130.7 (C_9*_), 110.9 (C_1″_), 102.3 (C_5_), 91.6 (C_1′_), 85.5 (C_2″_), 80.0 (C_4′_), 78.1 (C_5′_), 76.2 (C_4”_), 75.5 (C_2′_), 73.8 (C_3″_), 71.2 (C_3′_), 44.3 (C_5”_), 33.0 (C_6′_), 30.8, 30.7, 30.6, 30.4, 30.3, 30.3, 30.1, 30.0 (C_4*_-C_6*_, C_11*_-C_16*_), 28.1 (C_7*_), 28.0 (C_10*_), 27.6 (C_2*_), 27.1 (C_1*_), 23.7 (C_3*_), 14.4 (C_17*_). HRMS ESI− calcd. for C_34_H_54_N_5_O_10_^−^ (M + H)^−^ 692.3876 found 692.3890.

#### 3.1.18. Oxadiazole **12e**

Compound **12e** was obtained from the protected oxadiazole **11e** (20 mg, 21 μmol, 1 equiv.) as a colorless oil (8 mg, 60% yield): R*_f_* 0.15 (DCM/MeOH/NH_4_OH 14% 80/18/2); [α]_D_ + 1 (c 1.0, CH_3_OH); IR (film): 3058, 2912, 2840, 1658, 1435, 1275, 1244, 1198, 993, 799, 751; ^1^H NMR (500 MHz, MeOD) *δ* 8.10 (d, *J*_Ha-Hb_ = 8.8 Hz, 2 H, H_a_), 7.81 (d, *J*_H6-H5_ = 8.4 Hz, 1H, H_6_), 7.45 (t, *J*_Hd-He_ = 8.0 Hz, 2H, H_d_), 7.25 (t, *J*_He-Hd_ = 7.5 Hz, 1H, H_e_), 7.13–7.10 (m, 4 H, H_c_, H_b_), 5.78 (d, *J*_H1′-H2′_ = 3.0 Hz, 1H, H_1′_), 5.70 (d, *J*_H5-H6_ = 8.4 Hz, 1H, H_5_), 5.18 (s, 1H, H_1″_), 4.38 (td, *J*_H5′-H6′_ = 5.7 Hz, *J*_H5′-H4′_ = 3.1Hz, 1H, H_5′_), 4.17–4.14 (m, 2 H, H_2′_, H_3′_), 4.09–4.05 (m, 3 H, H_4′_, H_3″_, H_2″_), 3.99 (d, *J*_H4″-H5″_ = 3.5 Hz, 1H, H_4″_), 3.38–3.32 (m, 2H, H_6′ab_), 3.29–3.27 (m, *J*_H5″a-H5″b_ = 5.8 Hz, 1H, H_5″a_), 3.17–3.11 (m, 1H, H_5″b_); ^13^C NMR (125 MHz, MeOD) δ 176.6 (C_8′_), 169.5 (C_7′_), 166.0 (C_4_), 163.6 (C_9*_), 156.6 (C_10*_), 152.1 (C_2_), 142.2 (C_6_), 131.3 (C_d_), 131.2 (C_a_), 126.0 (C_e_), 121.3 (C_c_), 119.0 (C_b_), 110.8 (C_1″_), 102.4 (C_5_), 91.5 (C_1′_), 85.7 (C_2″_), 79.9 (C_4′_), 78.0 (C_5′_), 76.2 (C_4”_), 75.4 (C_2′_), 73.9 (C_3″_), 71.3 (C_3′_), 44.4 (C_5”_), 30.4 (C_11*_); HRMS ESI+ calcd. for C_29_H_32_N_5_O_11_^+^ (M + H)^+^ 626.2092 found 626.2078.

#### 3.1.19. Oxadiazole **12f**

Compound **12f** was obtained from the protected oxadiazole **11f** (44 mg, 41 μmol, 1 equiv.) as a colorless oil (22.8 mg, 63% yield): R*_f_* 0.15 (DCM/MeOH/NH_4_OH 14% 80/18/2); [α]_D_ + 8 (c 1.0, CH_3_OH); IR (film): 3052, 2926, 2854, 1698, 1410, 1275, 1203, 1132, 965, 799, 764, 750; ^1^H NMR (500 MHz, MeOD) *δ* 7.81 (d, *J*_H6-H5_ = 8.1Hz, 1H, H_6_), 5.77 (d, *J*_H1′-H2′_ = 3.2 Hz, 1H, H_1′_), 5.73 (d, *J*_H5-H6_ = 8.1Hz, 1H, H_5_), 5.41–5.36 (m, 2 H, H_8*_, H_9*_), 5.14 (s, 1H, H_1″_), 4.30 (td, *J*_H5′-H6′_ = 6.5 Hz, *J*_H5′-H4′_ = 2.5 Hz, 1H, H_5′_), 4.16–4.02 (m, 5 H, H_2′_, H_3′_, H_4′_, H_3″_, H_2″_), 3.98 (d, *J*_H4″-H5″_ = 4.8 Hz, 1H, H_4″_), 3.30–3.25 (m, 2 H, H_6′ab_), 3.21 (dd, *J*_H5″a-H4″_ = 14.9 Hz, *J*_H5″a-H5″b_ = 5.7 Hz, 1H, H_5″a_), 3.12 (dd, *J*_H5″b-H4″_ = 13.1Hz, *J*_H5″b-H5″a_ = 9.0 Hz, 1H, H_5″b_), 2.91 (t, *J*_H1*-H2*_ = 7.6 Hz, 2H, H_1*_), 2.00–1.95 (m, 4H, H_7*_, H_10*_), 1.81–1.76 (m, 2H, H_2*_), 1.37–1.29 ( m, 20H, H_3*_-H_6*_, H_11*_-H_16*_), 0.90 (t, *J*_H15*-H14*_ = 6.9 Hz, 3H, H_17*_); ^13^C NMR (125 MHz, MeOD) *δ* 181.8 (C_8′_), 168.7 (C_7′_), 166.1 (C_4_), 152.1 (C_2_), 142.2 (C_6_), 131.6 (C_8*_), 131.4 (C_9*_), 110.9 (C_1″_), 102.4 (C_5_), 91.5 (C_1′_), 85.6 (C_2″_), 80.0 (C_4′_), 78.0 (C_5′_), 76.2 (C_4”_), 75.5 (C_2′_), 73.8 (C_3″_), 71.3 (C_3′_), 44.3 (C_5”_), 33.6 (C_7*_), 33.5 (C_10*_), 33.0 (C_6′_), 30.7, 30.6, 30.5, 30.4, 30.3, 30.2, 30.0, 29.9 (C_4*_-C_6*_, C_11*_-C_16*_), 27.6 (C_2*_), 27.1 (C_1*_), 23.7 (C_3*_), 14.4 (C_17*_); HRMS ESI+ calcd. for C_34_H_56_N_5_O_10_^+^ (M + H)^+^ 694.4022 found 694.4012.

#### 3.1.20. Oxadiazole **12g**

Compound **12g** was obtained from the protected oxadiazole **11g** (20 mg, 20 μmol, 1 equiv.) as a colorless oil (12 mg, 63% yield): R*_f_* 0.15 (DCM/MeOH/NH_4_OH 14% 80/18/2); [α]_D_ + 1 (c 1.0, CH_3_OH); IR (film): 3028, 2913, 2841, 1660, 1435, 1275, 1133, 993, 800, 764, 750; ^1^H NMR (500 MHz, MeOD) *δ* 7.81 (d, J_H6-H5_ = 8.3 Hz, 1H, H_6_), 7.23 (d, J_Ha-Hb_ = 7.1Hz, 2 H, H_a_), 7.16–7.11 (m, 3 H, H_b_, H_c_), 5.76 (d, J_H1′-H2′_ = 3.1Hz, 1H, H_1′_), 5.72 (d, J_H5-H6_ = 8.2 Hz, 1H, H_5_), 5.14 (s, 1H, H_1″_), 4.29 (td, J_H5′-H6′_ = 6.5 Hz, J_H5′-H4′_ = 2.7 Hz, 1H, H_5′_), 4.15–4.01 (m, 5 H, H_2′_, H_3′_, H_4′_, H_3″_, H_2″_), 3.98 (d, J_H4″-H5″_ = 3.9 Hz, 1H, H_4″_), 3.30–3.26 (m, 2H, H_6′ab_), 3.20 (dd, J_H5″a-H4″_ = 15.3 Hz, J_H5″a-H5″b_ = 5.3 Hz, 1H, H_5″a_), 3.11 (dd, J_H5″b-H4″_ = 13.1Hz, J_H5″b-H5″a_ = 9.0 Hz, 1H, H_5″b_), 2.90 (t, _JH1*-H2*_ = 7.4 Hz, 2 H, H_1*_), 2.59 (t, J_H9*-H10*_ = 7.7 Hz, 2 H, H_9*_), 1.80–1.74 (m, 2 H, H_2*_), 1.63–1.57 (m, 2 H, H_8*_), 1.37–1.28 ( m, 10 H, H_3*_-H_7*_); ^13^C NMR (125 MHz, MeOD) *δ* 181.8 (C_8′_), 168.7 (C_7′_), 166.1 (C_4_), 152.1 (C_2_), 143.9 (C_10*_), 142.2 (C_6_), 129.4 (C_a_), 129.2 (C_b_), 126.6 (C_c_), 110.9 (C_1″_), 102.4 (C_5_), 91.5 (C_1′_), 85.6 (C_2″_), 79.9 (C_4′_), 78.1 (C_5′_), 76.2 (C_4”_), 75.5 (C_2′_), 73.8 (C_3″_), 71.2 (C_3′_), 44.3 (C_5”_), 36.9 (C_9*_), 32.7 (C_6′_), 30.5, 30.4, 30.3, 30.2, 30.1, 29.9 (C_3*_-C_8*_), 27.6 (C_2*_), 27.1 (C_1*_); HRMS ESI+ calcd. for C_32_H_46_N_5_O_10_^+^ (M + H)^+^ 660.3239 found 660.3226.

#### 3.1.21. Oxadiazole **12h**

Compound **12h** was obtained from the protected oxadiazole **11h** (42 mg, 42 μmol, 1 equiv.) as a white solid (19 mg, 68% yield) and as a 1/1 mixture of stereoisomers: R*_f_* 0.15 (DCM/MeOH/NH_4_OH 14% 80/18/2); [α]_D_ + 1.4 (c 1.0, CH_3_OH); IR (film): 2925, 2855, 1688, 1579, 1464, 1274, 1126, 748; ^1^H NMR (500 MHz, MeOD) *δ* 7.80 (d, *J*_H5-H6_ = 8.1 Hz, 0.5H, H_6_), 7.79 (d, *J*_H5-H6_ = 8.1 Hz, 0.5H, H_6_), 5.75 (d, *J*_H1′-H2′_ = 3.1 Hz, 1H, H_1′_), 5.72 (d, *J*_H5-H6_ = 8.1 Hz, 1H, H_5_), 5.14 (s, 0.5H, H_1″_), 5.12 (s, 0.5H, H_1″_), 4.31 (td, *J*_H5′-H6′a_ = *J*_H5′-H6′b_ = 6.0, *J*_H4′-H5′_ = 3.0 Hz, 1H, H_5′_), 4.26–4.16 (m, 2H, H_3**_), 4.16–4.09 (m, 2H, H_1*_, H_2′_), 4.09–4.01 (m, 3H, H_4″_, H_4′_, H_3″_), 3.97 (d, *J*_H2″-H3″_ = 3.5 Hz, 1H, H_2″_), 3.38–3.32 (m, 1H, H_6′a_), 3.30–3.26 (m, 1H, H_5″a_), 3.23 (dd, *J*_H6′a-H6′b_ = 15.0, *J*_H5′-H6′b_ = 6.0 Hz, 1H, H_6′b_), 3.14 (dd, *J*_H5″a-H5″b_ = 13.0, *J*_H4″-H5″b_ = 8.2 Hz, 1H, H_5″b_), 2.17–1.95 (m, 2H, H_2*_), 1.43–1.27 (m, 14H, H_3*_-H_9*_), 1.25 (t, *J*_H3**-H4**_ = 7.1 Hz, 3H, H_4**_), 0.90 (t, *J*_H9*-H10*_ = 6.9 Hz, 3H, H_10*_); ^13^C NMR (125 MHz, MeOD) *δ* 178.28, 178.22 (C_2**_), 170.5, 170.4 (C_8′_), 169.22, 169.18 (C_7′_), 166.16 (C_4_), 152.1 (C_2_), 142.2 (C_6_), 111.1, 111.0 (C_1″_), 102.4 (C_5_), 91.60, 91.57 (C_1′_), 85.9 (C_4′_), 80.0 (C_4″_), 78.3, 78.1 (C_5′_), 76.3 (C_2″_), 75.5 (C_1*_), 73.9 (C_3′_), 71.34, 71.30 (C_2′_), 63.2 (C_3**_), 44.3 (C_5″_), 33.1, 31.2, 31.0, 30.6, 30.5, 30.2, 28.1, 23.7 (C_2*_-C_9*_), 30.4 (C_6′_), 14.5, 14.4 (C_4**_, C_10*_); HRMS ESI+ calcd. for C_30_H_48_N_5_O_12_^+^ (M + H)^+^ 670.3294 found 670.3320.

#### 3.1.22. Oxadiazole **12i**

Compound **12i** was obtained from the protected oxadiazole **11i** (34 mg, 32 μmol, 1 equiv.) as a colorless oil (5.3 mg, 22% yield): R*_f_* 0.15 (DCM/MeOH/NH_4_OH 14% 80/18/2); [α]_D_ + 1 (c 1.0, CH_3_OH); IR (film): 2923, 1690, 1275, 1260, 764, 750; ^1^H NMR (500 MHz, MeOD) *δ* 7.89 (d, *J*_H6-H5_ = 8.1Hz, 1H, H_6_), 5.81 (d, *J*_H1′-H2′_ = 2.7 Hz, 1H, H_1′_), 5.74 (d, *J*_H5-H6_ = 8.1Hz, 1H, H_5_), 5.03 (s, 1H, H_1″_), 4.30 (td, *J*_H5′-H6′_ = 6.3 Hz, *J*_H5′-H4′_ = 2.2 Hz, 1H, H_5′_), 4.14–4.11 (m, 2 H, H_2′_, H_3′_), 4.07–4.04 (m, 1H, H_4′_), 3.98 (dd, *J*_H3″-H2″_ = 6.6 Hz, *J*_H3″-H4″_ = 4.8 Hz, 1H, H_3″_), 3.93 (dd, *J*_H2″-H3″_ = 4.6 Hz, *J*_H2″-H1″_ = 1.2 Hz, 1H, H_2″_), 3.88 (td, *J*_H4″-H5″_ = 7.1 Hz, *J*_H4″-H3″_ = 3.5 Hz, 1H, H_4″_), 3.24 (dd, *J*_H6′a-H5′_ = 8.7 Hz, *J*_H6′a-H6′b_ = 6.3 Hz, 1H, H_6′a_), 3.19 (dd, *J*_H6′b-H5′_ = 8.7 Hz, *J*_H6′b-H5′_ = 6.3 Hz, 1H, H_6′b_), 3.06–3.00 (m, 1H, H_1*_), 2.91 (dd, *J*_H5″a-H4″_ = 13.3 Hz, *J*_H5″a-H5″b_ = 3.7 Hz, 1H, H5″a), 2.79 (dd, *J*_H5″b-H4″_ = 13.3 Hz, *J*_H5″b-H5″a_ = 7.5 Hz, 1H, H_5″b_), 1.79–1.69 (m, 4 H, H_2*_,H_2**_), 1.35–1.17 ( m, 34 H, H_3**_-H_10**_, H_3*_-H_10*_), 0.91–0.88 ( 6H, H_11*_, H_11**_); ^13^C NMR (125 MHz, MeOD) *δ* 184.5 (C_8′_), 168.6 (C_7′_), 166.2 (C_4_), 152.2 (C_2_), 142.0 (C_6_), 111.1 (C_1″_), 102.4 (C_5_), 91.0 (C_1′_), 86.3 (C_4′_), 84.5 (C_4″_), 78.5 (C_5′_), 76.8 (C_2”_), 75.8 (C_2′_), 73.3 (C_3″_), 71.3 (C_3′_), 45.3 (C_5”_), 39.6 (C_1*_), 34.6 (C_2*_), 34.5 (C_2**_), 33.0 (C_9*_, C_9**_), 30.8, 30.7, 30.4, 30.4 (C_4*_-C_8*_, C_4**_-C_8**_), 28.2 (C_3*_, C_3**_), 23.7 (C_10*_, C_10**_), 14.4 (C_11*_, C_11**_); HRMS ESI+ calcd. for C_38_H_66_N_5_O_10_^+^ (M + H)^+^ 752,4804 found 752.4804.

#### 3.1.23. Oxadiazole **12j**

To a solution of oxadiazole **12h** (2.5 mg, 3.7 µmol, 1 equiv.) in methanol (0.4 mL) at 0 °C was added a solution of NH_4_HCO_3_ in water (0.1 M, 0.5 mL) and trimethylamine (14 µL, 99 µmol, 27 equiv.). After 48 h at 0 °C, the reaction mixture was diluted in 5 mL water, and the solution was freeze-dried. The residue was purified on a reverse phase column (Sep-Pak Cartridges C18) using a mixture of an aqueous solution NH_4_HCO_3_ (0.1 M) and acetonitrile (1/0 to 1/1, v/v). The fractions containing the product were freeze-dried to afford product **12j** as a white powder (0.4 mg) and as a 1/1 mixture of stereoisomers in 17% yield: R*_f_* 0.07 (DCM/MeOH/NH_4_OH 14% 80/18/2); ^1^H NMR (500 MHz, MeOD) *δ* 7.87 (d, *J*_H5-H6_ = 8.1 Hz, 0.5H, H_6_), 7.85 (d, *J*_H5-H6_ = 8.1 Hz, 0.5H, H_6_), 5.80 (d, *J*_H1′-H2′_ = 3.4 Hz, 0.5H, H_1′_), 5.79 (d, *J*_H1′-H2′_ = 3.4 Hz, 0.5H, H_1′_), 5.74 (d, *J*_H5-H6_ = 8.1 Hz, 0.5H, H_5_), 5.74 (d, *J*_H5-H6_ = 8.1 Hz, 0.5H, H_5_), 5.12 (s, 0.5H, H1″), 5.09 (s, 0.5H, H1″), 4.37–4.31 (m, 1H, H_5′_), 4.18–3.85 (m, 6H, H_1*_, H_2′_, H_4″_, H_4′_, H_3″_, H_2″_), 3.14–2.72 (m, 4H, H_6′a_, H_5″a_, H_6′b_, H_5″b_), 2.18–1.98 (m, 2H, H_2*_), 1.40–1.22 (m, 14H, H_3*_-H_9*_), 0.90 (t, *J*_H9*-H10*_ = 6.9 Hz, 3H, H_10*_); ^13^C NMR (125 MHz, MeOD) *δ* 181.4 (C_2**_), 170.3 (C_8′_), 169.0 (C_7′_), 166.3 (C_4_), 152.3, 152.2(C_2_), 142.4, 142.2 (C_6_), 110.4, 110.0 (C_1″_), 102.57, 102.51 (C_5_), 91.7 (C_1′_), 86.4 (C_4′_), 80.0 (C_4″_), 78.1 (C_5′_), 76.5 (C_2″_), 75.55, 75.49 (C_1*_), 73.7, 73.6 (C_3′_), 71.4, 71.3 (C_2′_), 44.94, 44.87 (C_5″_), 33.1, 32.1, 31.9, 30.8, 30.7, 30.6, 30.5, 26.9, 23.7 (C_2*_-C_9*_, C_6′_), 14.5 (C_10*_); HRMS ESI+ calcd. for C_28_H_44_N_5_O_12_^+^ (M + H)^+^ 642.2942 found 642.2979.

### 3.2. Enzyme Assays

The inhibitory activity of the synthesized compounds **1–5** was determined as described in reference [41,42] except that compounds were tested at a final concentration of 10% DMSO. The compounds were evaluated on His-tagged MraY transferase purified from *Aquifex aeolicus* (MraY_AA_) prepared as previously described by Chung et al. [37]. The assays were performed as previously described by Stachyra et al. [53]. 

### 3.3. Antibacterial Activity

Antibacterial activity was determined as previously described [41,42], but molecules were solubilized in 100% DMSO (cell culture grade) at 1 mg/mL concentration and 10-fold diluted in MHB just before utilization. The MHB-diluted solutions were then serially diluted in 5% DMSO-MHB, at final concentrations ranging from 50 mg/mL to 0.005 mg/mL for compounds and 6.25 to 5% for DMSO. Growth controls for strains indicated that DMSO does not inhibit growth at the highest concentration used in the test. 

### 3.4. Docking 

Docking experiments were performed according to the protocol previously described [41,42].

## 4. Conclusions

We report the synthesis of new inhibitors of the bacterial MraY_AA_ transferase displaying an aminoribosyl uridine scaffold substituted in 5′ position by an oxadiazole linker bearing various hydrophobic substituents. Their straightforward synthesis relies on the microwave-assisted *O*-acylation of an amidoxime by various acyl chlorides followed by cyclization into the oxadiazole. Their biological activity was evaluated in vitro on purified MraY_AA_ and compared to that of a reference compound with a *N*-triazole as another heterocyclic linker. Nine out of the ten synthetized compounds revealed MraY inhibition with IC_50_ ranging from 0.77 to 27.48 µM, the most active compound with a C_10_ alkyl chain being slightly more active than the *N*-triazole reference compound. The binding mode of the inhibitors has been studied by docking experiments. The in cellulo evaluation of the synthetized inhibitors on different Gram-positive and Gram-negative bacterial strains only led to the poor activity of the two compounds on Gram-positive bacteria, showing that the further optimization of the lipophilic side chain is still required to improve the antibacterial activity.

## Figures and Tables

**Table 1:** Optimization of the nucleophilic opening of the epoxide F by potassium cyanide.

Entry	KCN (equiv.)	Solvent	NH_4_Cl (equiv.)	*T* (°C)	Duration	Yield of E (%)
**1**	8	DMF	1.5	100	16 h	23-68 *^a,b or c^*
**2**	6	DMF	1.5	55	16 h	28 *^b^*
**3**	6	CH_3_CN	1.5	82	16 h	30 *^b^*
**4**	6	CH_3_CN	-	82	16 h	10 *^b^*
**5**	6	Toluene	1.5	100	16 h	traces
**6**	3	Toluene	-	100	16 h	traces
**7**	6	MeOH/H_2_O 8/1	1.5	60	2 h	84 ^c^
**8**	3	MeOH/H_2_O 8/1	1.5	60	16 h	62 *^b^*
**9**	5	MeOH/H_2_O 8/1	2.5	60	16 h	94 ^c^

*^a^* Partial deprotection of silyl ethers*. ^b^* Percentage of conversion by NMR. *^c^* Isolated yield.

**Table 2:** Conditions for the one-pot synthesis of oxadiazole **11b**.

Entry	DBU (equiv.)	t (min)	Yield of 11b *^a^* (%)
1	2.0	15	28
2	2.5	15	30
3	2.5	30	55

*^a^* Isolated yield.

**Table 3:** Isolated yields for the synthesis of compounds **11a**–**i** and **12a**–**i**.

Acyl Chlorides	R	11 Yield *^a^* (%)	12 Yield *^a^* (%)
**3a**	-(CH_2_)_9_-CH_3_	67	85
**3b**	-(CH2)14-CH3	55	59
**3c**	-(CH2)17-CH3	75	40
**3d**	-(CH2)7-CH=CH-(CH2)7-CH3 (Z)	72	66
**3e**	-C_6_H_4_-O-C_6_H_5_	42	60
**3f**	-(CH_2_)_7_-CH=CH-(CH_2_)_7_-CH_3_ (*E*)	83	63
**3g**	-(CH_2_)_9_-Ph	58	63
**3h**	-CH((CH_2_)_8_-CH_3_))(CO_2_Et)	58	68
**3i**	-CH-((CH_2_)_10_)_2_	74	22 *^b^*

*^a^* Isolated yields. *^b^* Part of the starting compound **11i** was recovered.

**Table 4:** Inhibitory activity of compounds **12a–12j** against MraY_AA_.

Compound	R	IC_50_ (µM) *^a^*^, *b*^
Tunicamycin		0.026 ± 0.00
**13**		1.1 ± 0.0
**12a**	-(CH_2_)_9_-CH_3_	0.8 ± 0.1
**12b**	-(CH_2_)_14_-CH_3_	9.3 ± 0.2
**12c**	-(CH_2_)_17_-CH_3_	15.8 ± 1.0
**12d**	-(CH_2_)_7_-CH=CH-(CH_2_)_7_-CH_3_ (*Z*)	18.9 ± 0.9
**12e**	-C_6_H_4_-O-C_6_H_5_	3.2 ± 0.2
**12f**	-(CH_2_)_7_-CH=CH-(CH_2_)_7_-CH_3_ (*E*)	17.3 ± 0.3
**12g**	(CH_2_)_9_-Ph	5.8 ± 0.1
**12h**	-CH-((CH_2_)_8_-CH_3_)-(CO_2_Et)	27.5 ± 0.7
**12i**	-CH-((CH_2_)_10_)_2_	N.I. *^e^*
**12j**	-CH-((CH_2_)_8_-CH_3_)-(CO_2_^−^)	2.9 ± 0.6

*^a^* Experiments were performed in triplicate, and each experiment was repeated at least twice, except for tunicamycin as a control, which was tested twice. *^b^* All compounds were tested at a final concentration of 10% DMSO to ensure complete solubility. *^e^* N.I.: No inhibition.

## Data Availability

The data presented in this study are available in Supplementary Material.

## Supplementary Materials

The following supporting information can be downloaded at: https://www.mdpi.com/article/10.3390/antibiotics11091189/s1, ^1^H and ^13^C spectra of all compounds. Table S1: Antibacterial activity of compounds **12a–12i** and reference compounds.
