# Supplementary material for: New MraYAA Inhibitors with an Aminoribosyl Uridine Structure and an Oxadiazole
Source: Antibiotics (Basel). 2022 Sep 2;11(9):1189. doi: 10.3390/antibiotics11091189 (PMC9495235; doi:10.3390/antibiotics11091189)
Supplement: Supplementary file 1 [file antibiotics-11-01189-s001.zip › antibiotics-1835940-supplementary.pdf]

New MraY$_{AA}$ Inhibitors with an Aminoribosyl Uridine Structure and an Oxadiazole

Hongwei Wan [1], Raja Ben Othman [1], Laurent Le Corre [1], Mélanie Poinsot [1], Martin Oliver [1], Ana Amoroso [2], Bernard Joris [2], Thierry Touzé [3], Rodolphe Auger [3], Sandrine Calvet-Vitale [1], Michaël Bosco [1,*] and Christine Gravier-Pelletier [1,*]

1 Université Paris Cité, CNRS, Laboratoire de Chimie et de Biochimie Pharmacologiques et Toxicologiques, F-75006 Paris, France
2 Unité de Physiologie et Génétique Bactériennes, Centre d'Ingénierie des Protéines, Département des Sciences de la Vie, Université de Liège, Sart Tilman, B4000 Liège, Belgium
3 Institute for Integrative Biology of the Cell (I2BC), CNRS, Université Paris Sud, CEA, F-91405 Orsay, France
* Correspondence: michael.bosco@u-paris.fr (M.B.); christine.gravier-pelletier@u-paris.fr (C.G.-P.); Tel.: +33-176-534 246 (M.B.); +33-176-534-228 (C.G.-P.)

ELECTRONIC SUPPLEMENTARY MATERIAL

Table of contents

^{1}H and ^{13}C Spectra 1
Protected Nitrile **1**........ 2
Protected Amidoxime **2**........ 3
10-phenyldecanoic acid **4**........ 4
2-(Ethoxycarbonyl)undecanoic acid **6**........ 5
2-Decyldodecanoic acid **9**........ 6
Protected oxadiazole **11a**........ 7
Protected oxadiazole **11b**........ 8
Protected oxadiazole **11c**........ 9
Protected oxadiazole **11d**........ 10
Protected oxadiazole **11e**........ 11
Protected oxadiazole **11f**........ 12
Protected oxadiazole **11g**........ 13
Protected oxadiazole **11h**........ 14
Protected oxadiazole **11i**........ 15
Oxadiazole **12a**........ 16
Oxadiazole **12b**........ 17
Oxadiazole **12c**........ 18
Oxadiazole **12d**........ 19
Oxadiazole **12e**........ 20
Oxadiazole **12f**........ 21
Oxadiazole **12g**........ 22
Oxadiazole **12h**........ 23
Oxadiazole **12i**........ 24
Oxadiazole **12j**........ 25
Table S1. Antibacterial activity of compounds **12a**-**12i**, and reference compounds. 26

Protected Nitrile 1

^{1}H NMR (500 MHz, $CDCl_3$)

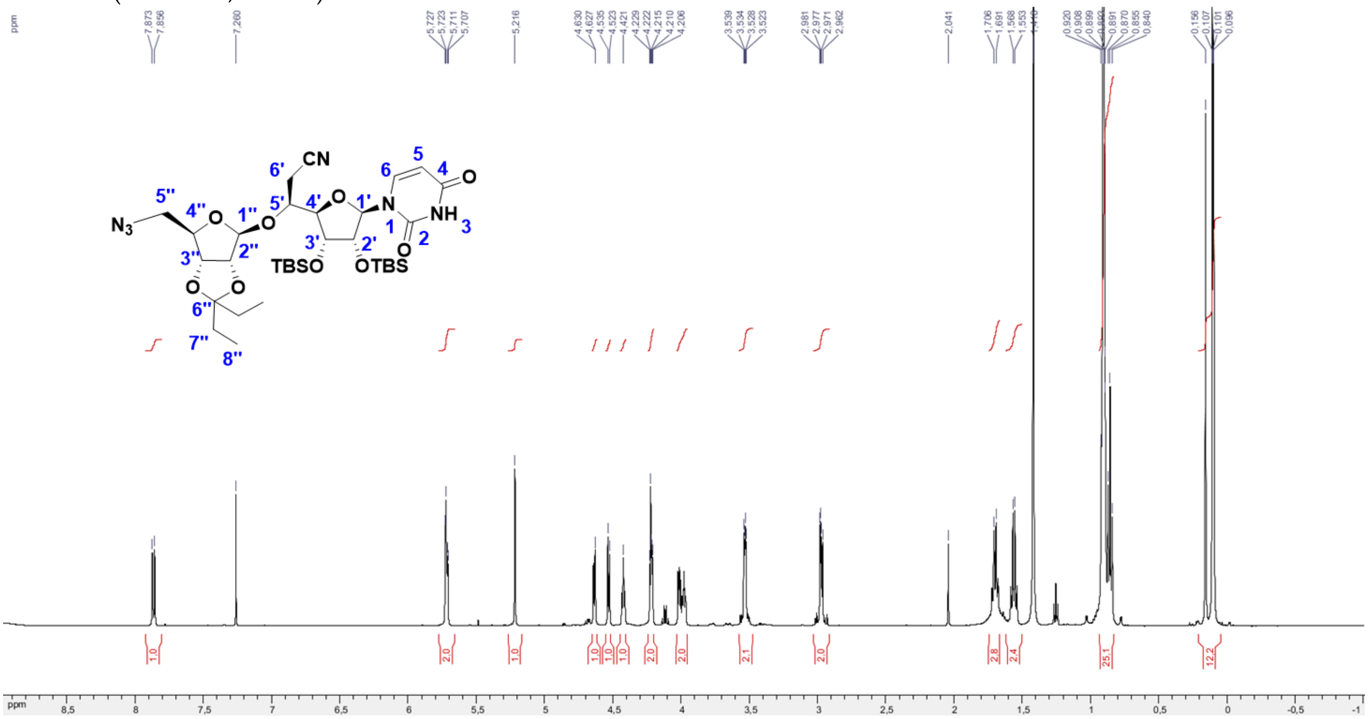

^{13}C NMR (125 MHz, $CDCl_3$)

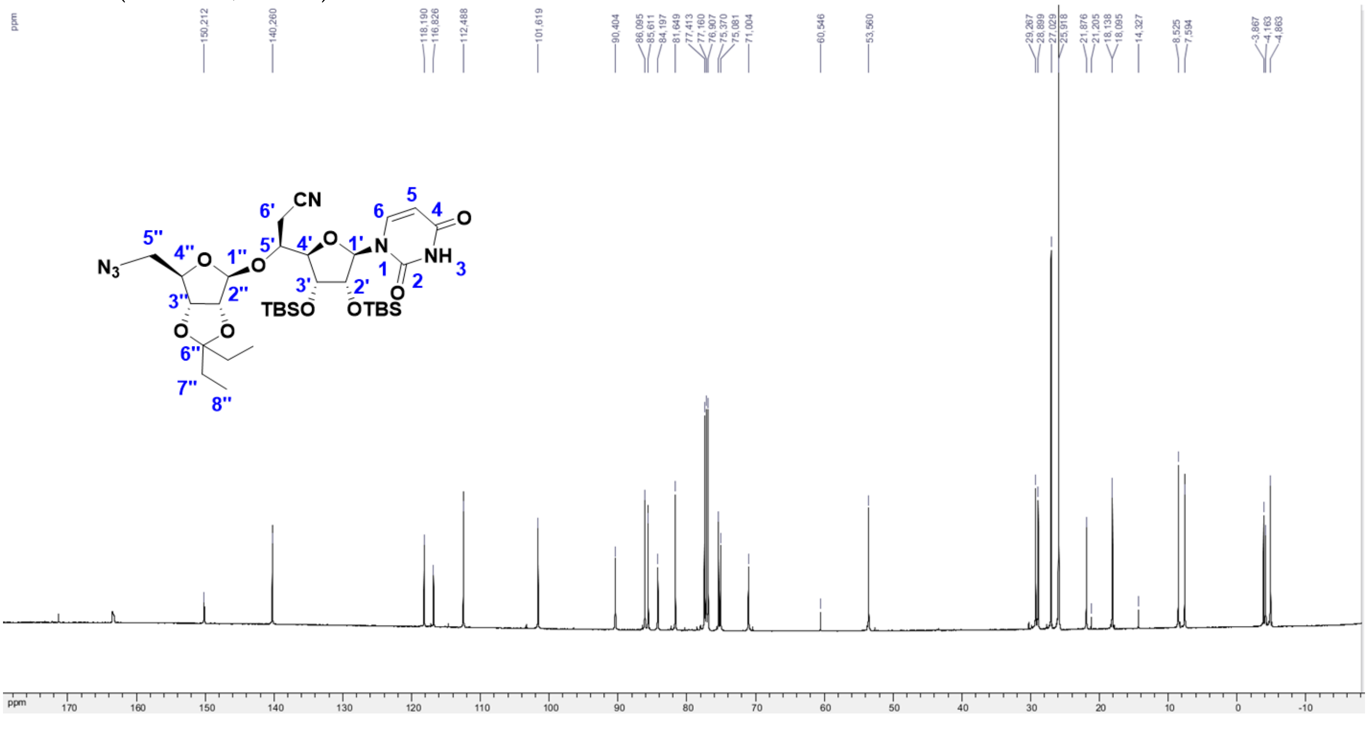

Protected Amidoxime 2

^{1}H NMR (500 MHz, $CDCl_3$)

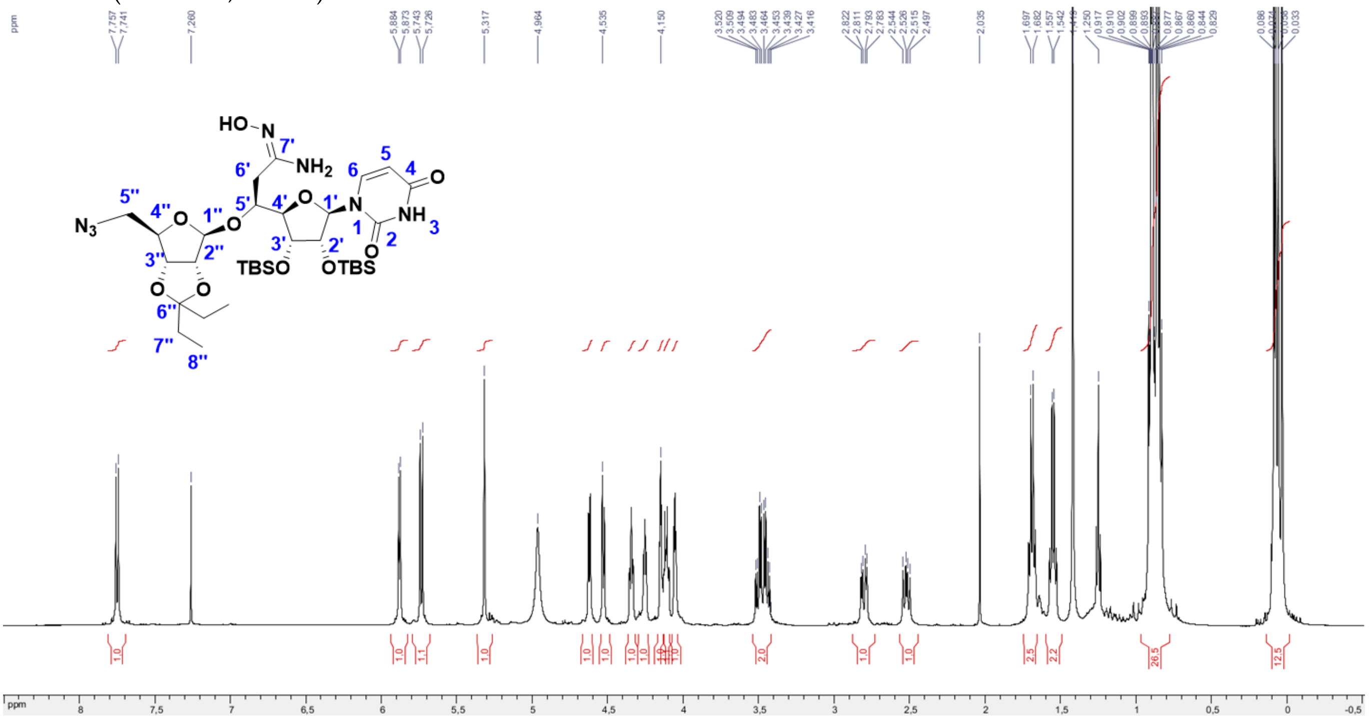

^{13}C NMR (125 MHz, $CDCl_3$)

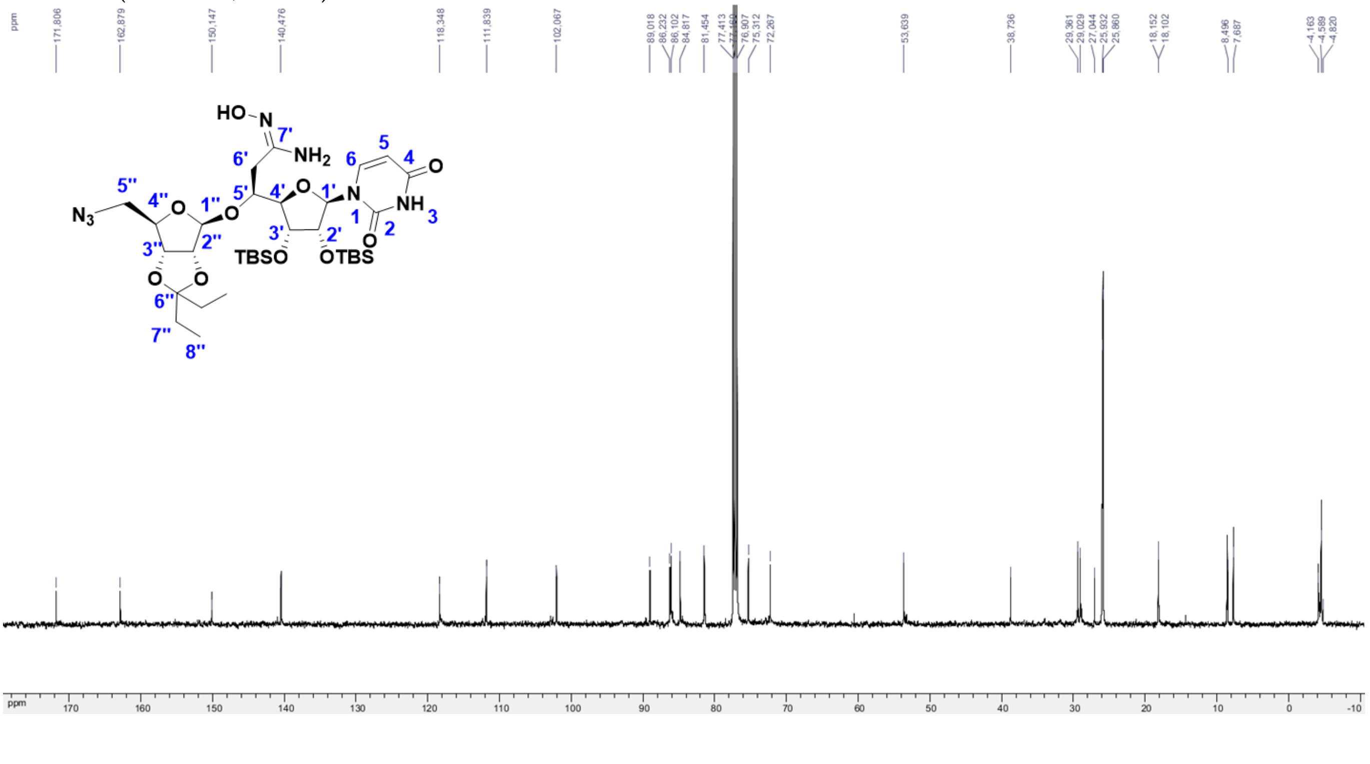

10-phenyldecanoic acid 4

^1H NMR (500 MHz, $CDCl_3$)

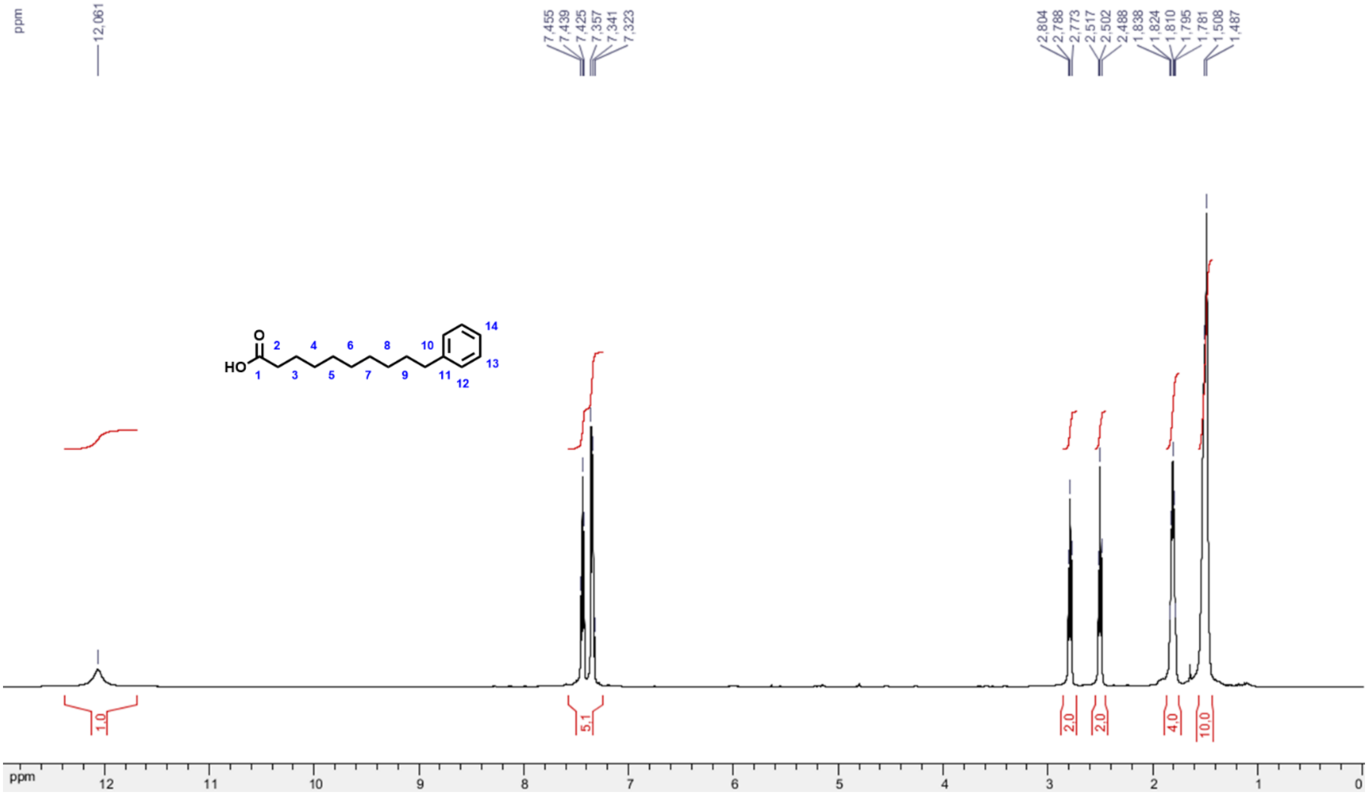

2-(Ethoxycarbonyl)undecanoic acid 6

^{1}H NMR (500 MHz, $CDCl_3$)

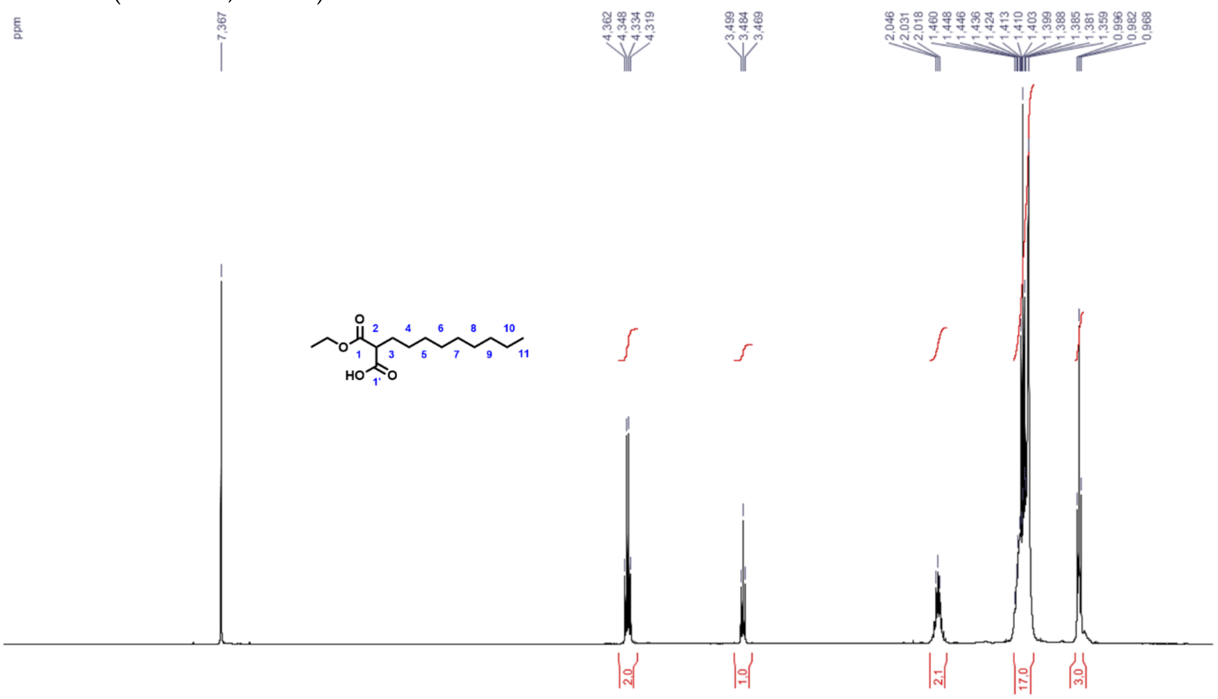

^{13}C NMR (125 MHz, $CDCl_3$)

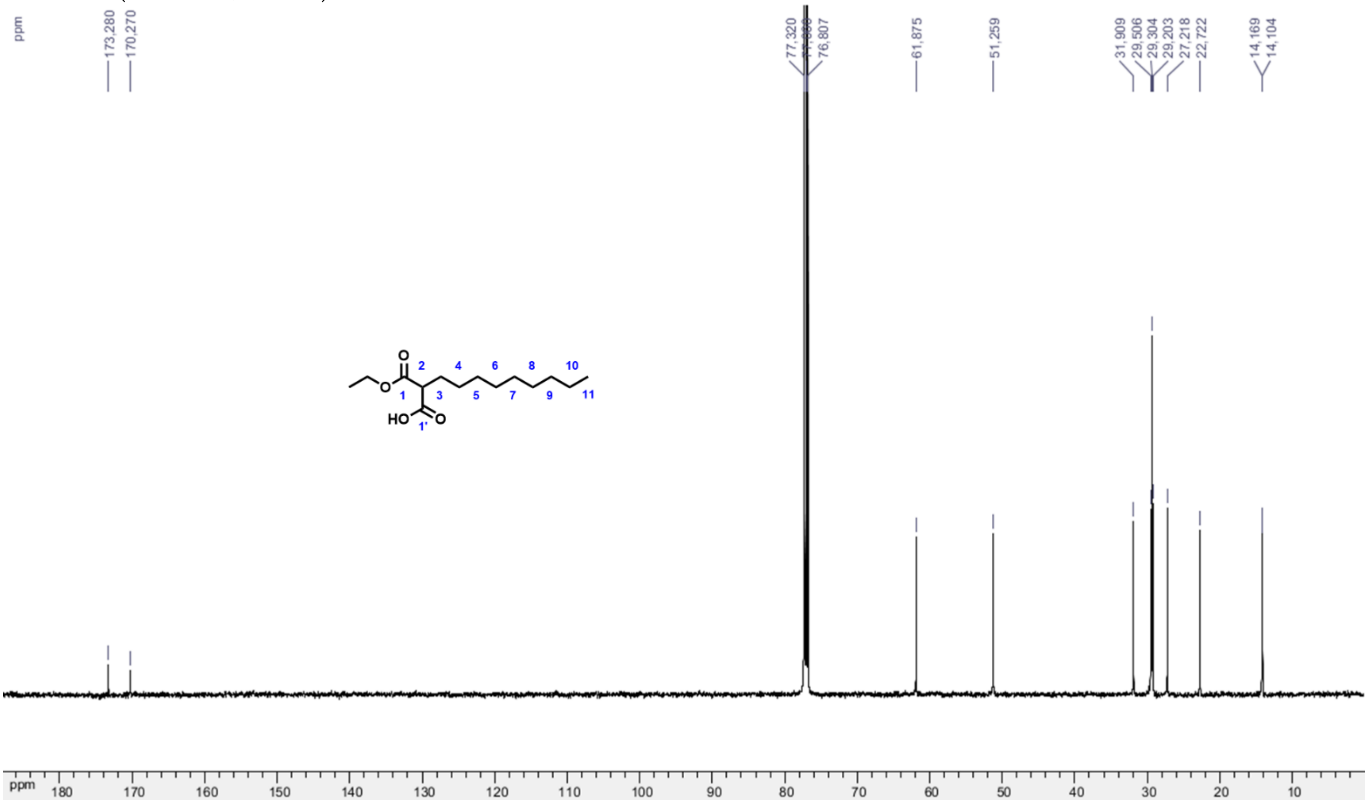

2-Decyldodecanoic acid 9

^{1}H NMR (500 MHz, $CDCl_3$)

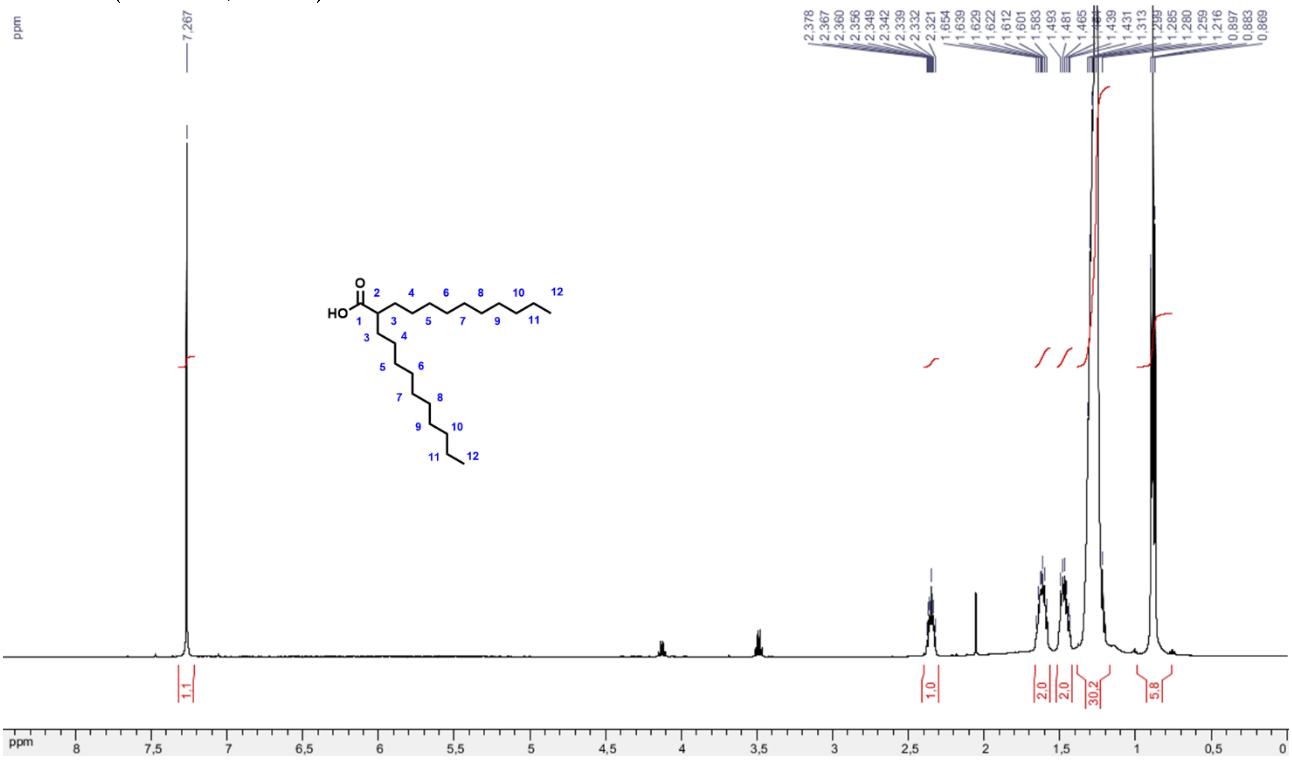

^{13}C NMR (125 MHz, $CDCl_3$)

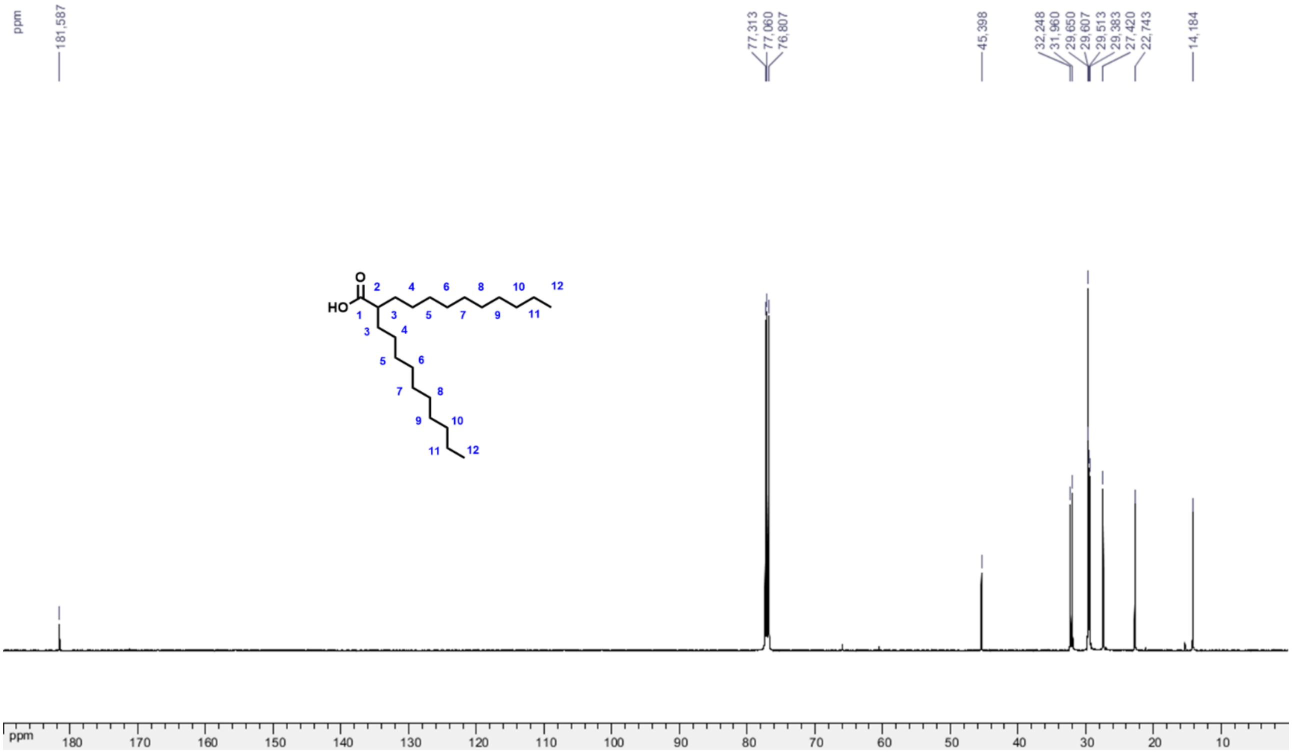

Protected oxadiazole 11a

^1H NMR (500 MHz, $CDCl_3$)

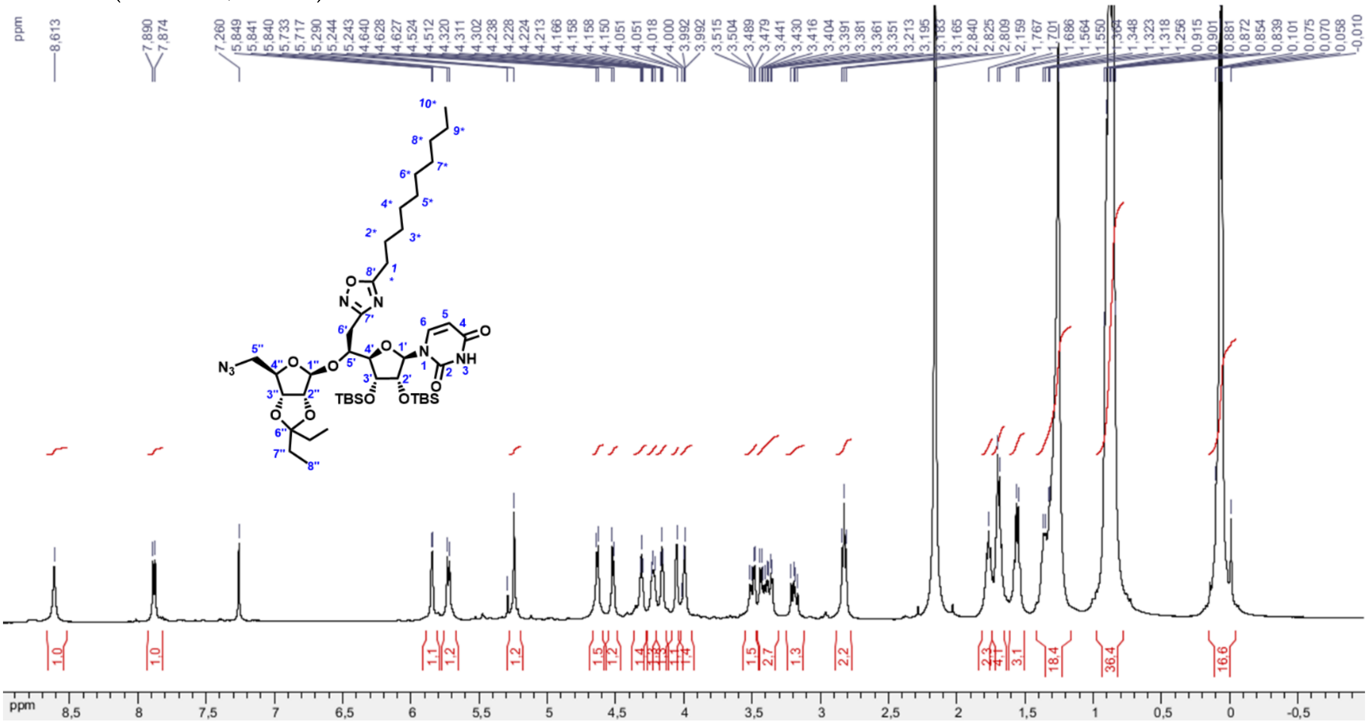

^{13}C NMR (125 MHz, $CDCl_3$)

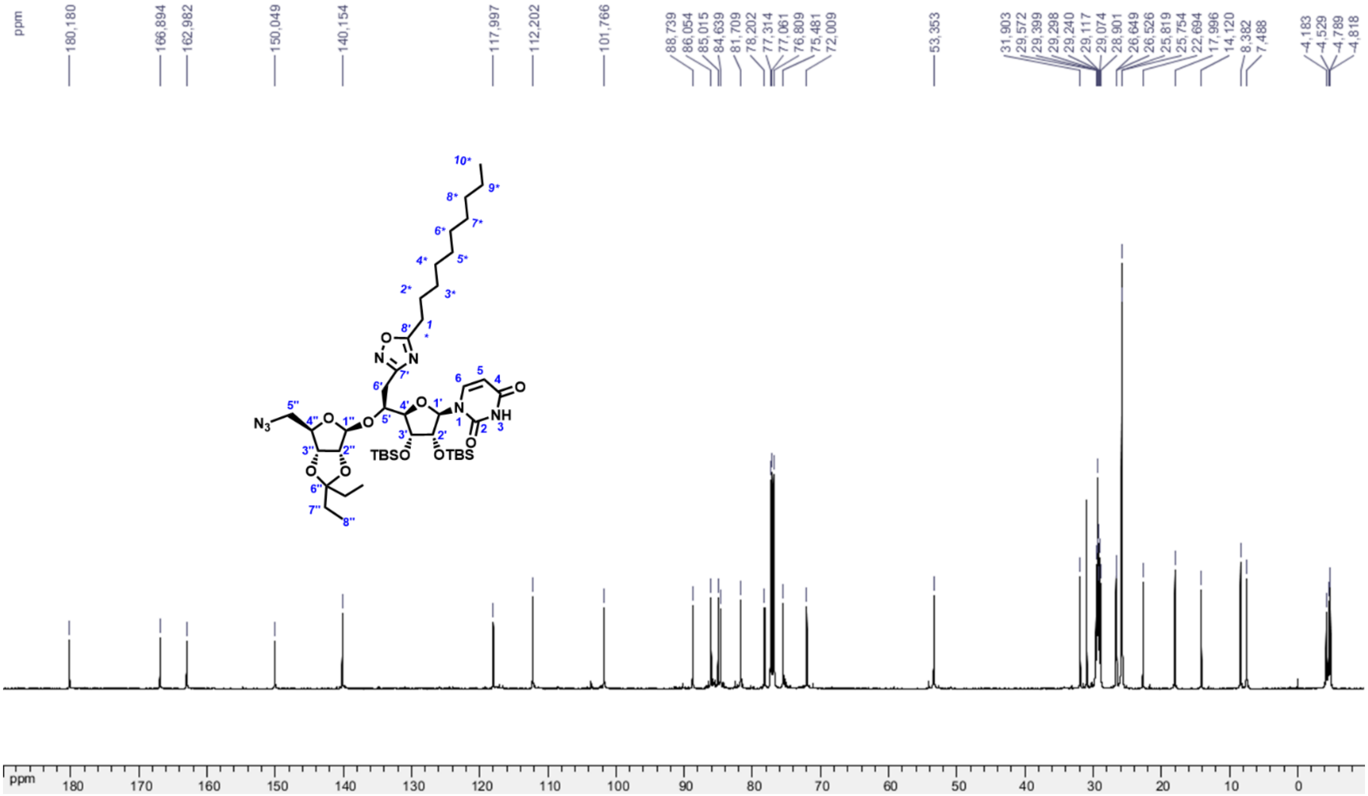

Protected Oxadiazole 11b

^{1}H NMR (500 MHz, $CDCl_3$)

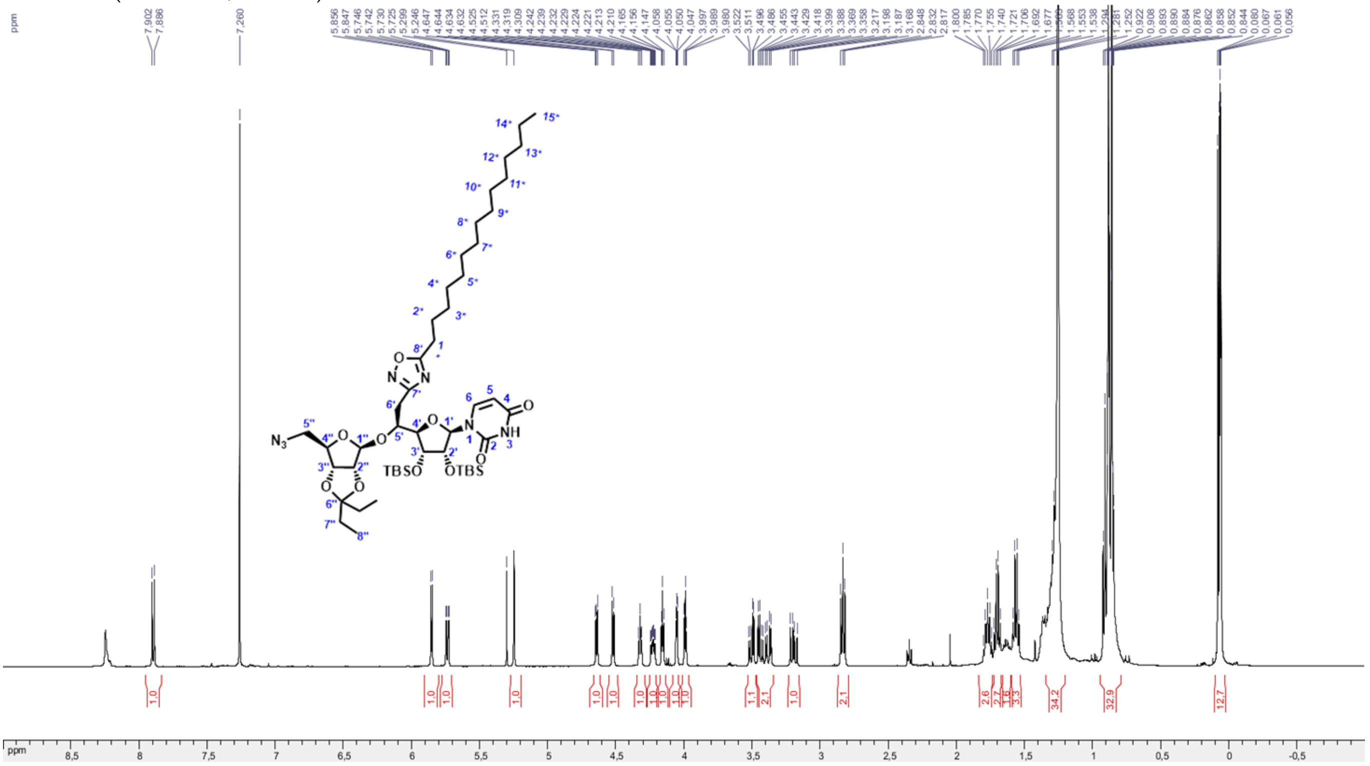

^{13}C NMR (125 MHz, $CDCl_3$)

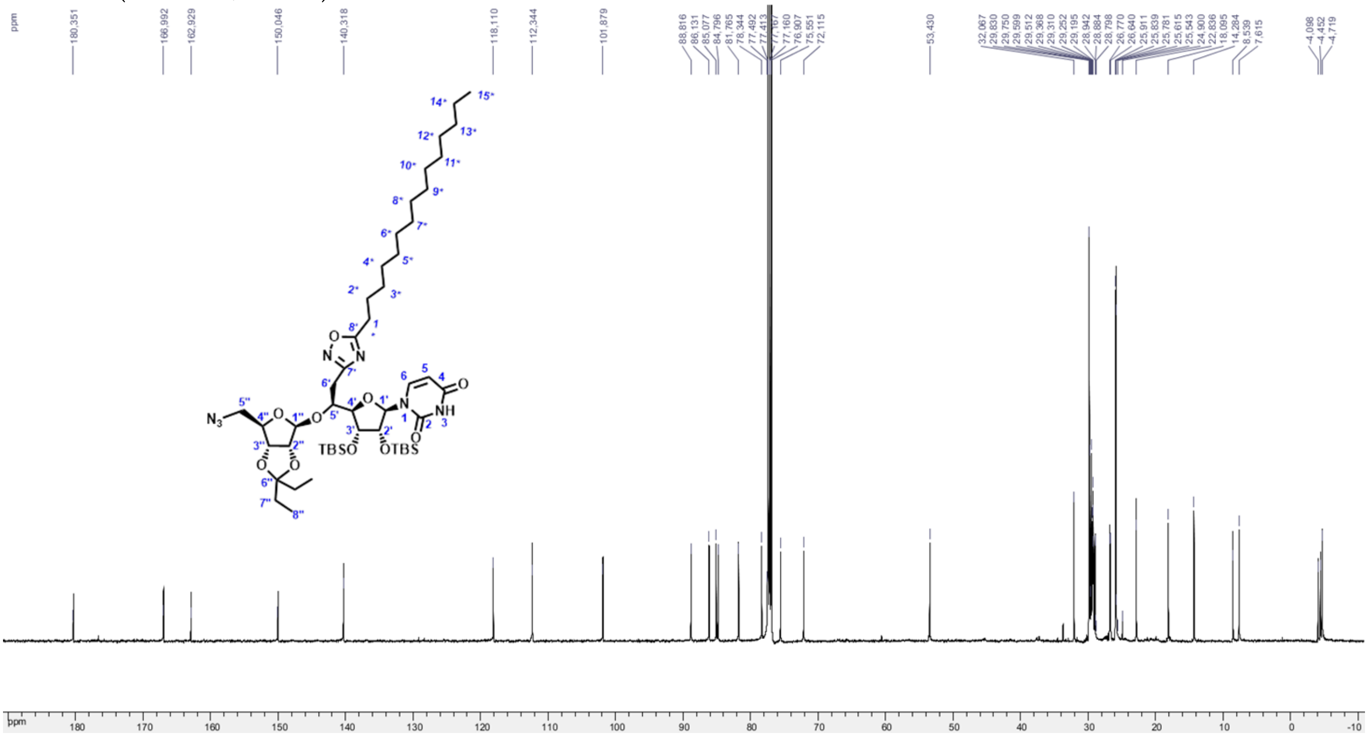

Protected Oxadiazole 11c

^{1}H NMR (500 MHz, $CDCl_3$)

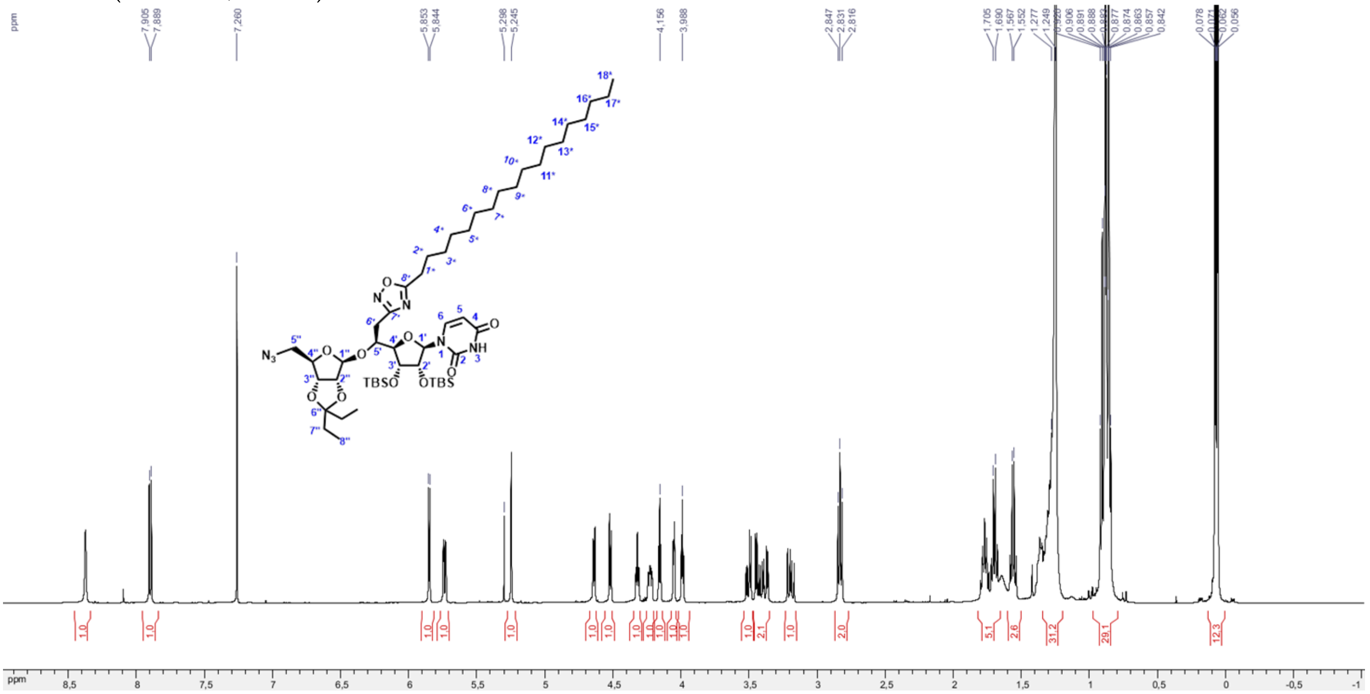

^{13}C NMR (125 MHz, $CDCl_3$)

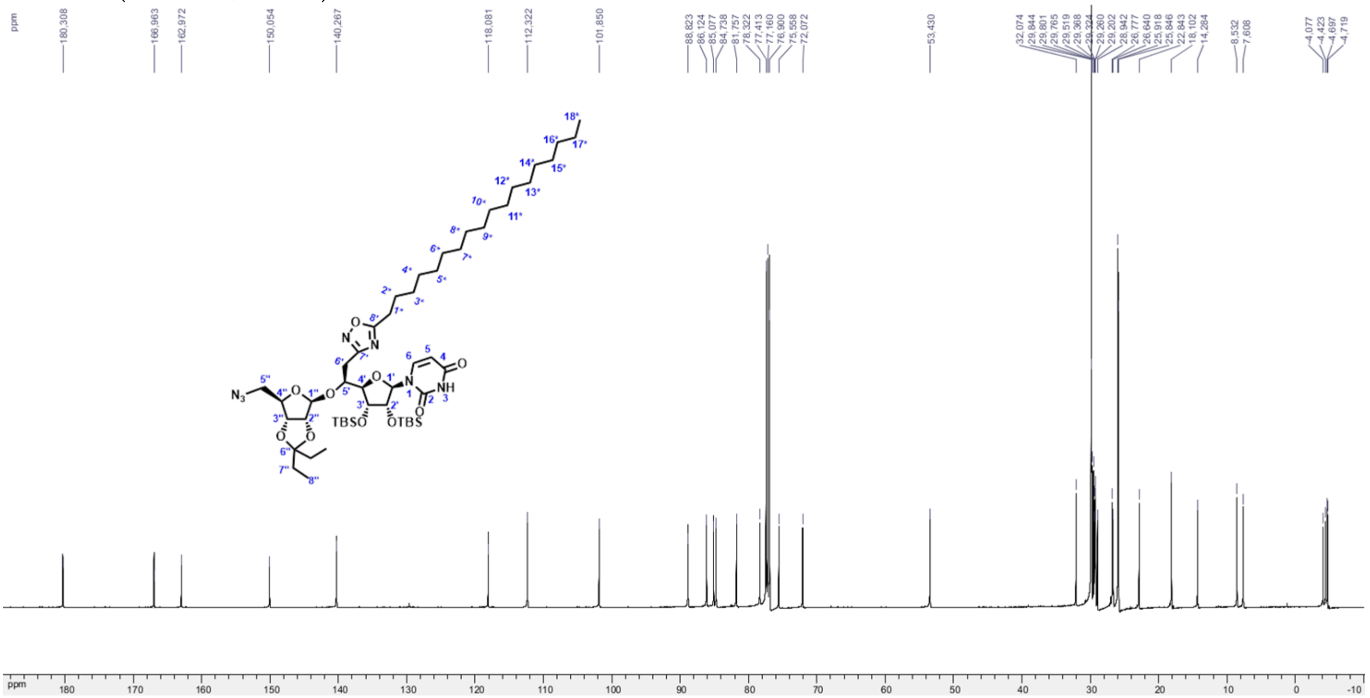

Protected Oxadiazole 11d

^{1}H NMR (500 MHz, CDCl$_3$)

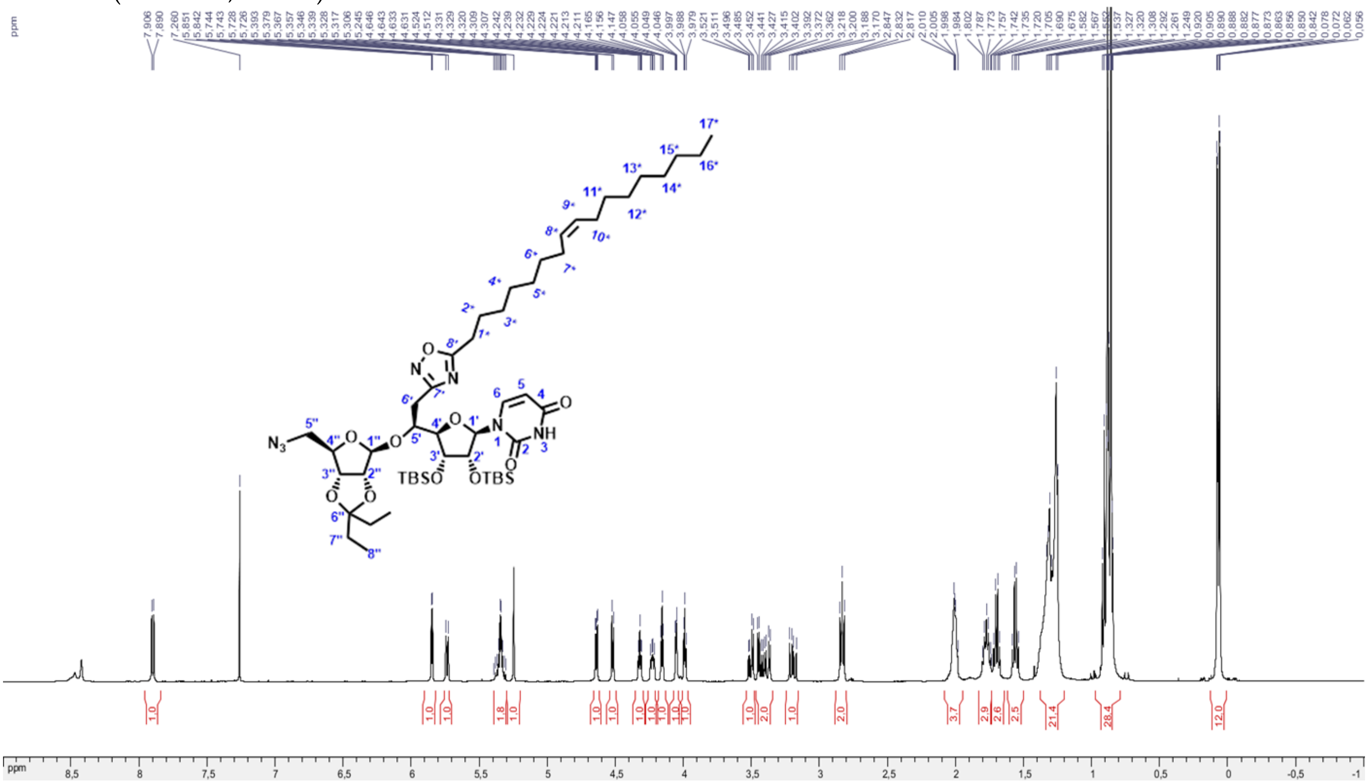

^{13}C NMR (125 MHz, CDCl$_3$)

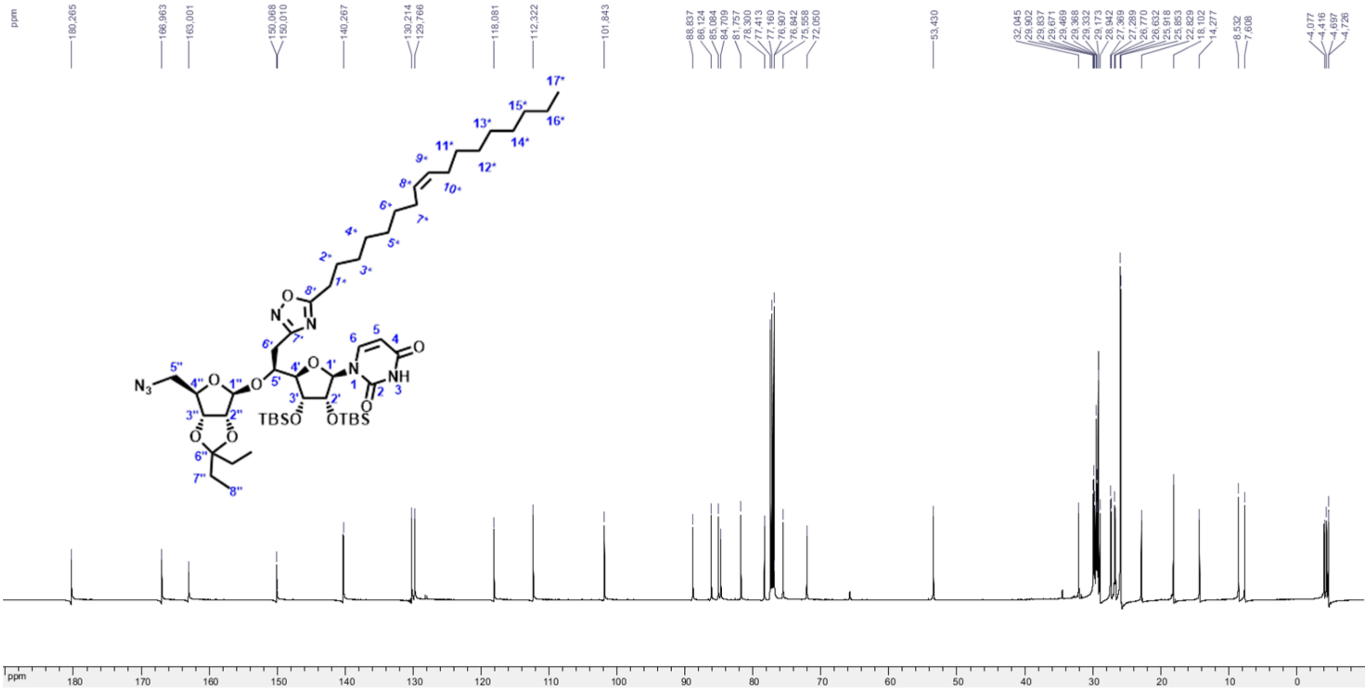

Protected Oxadiazole 11e

^{1}H NMR (500 MHz, $CDCl_3$)

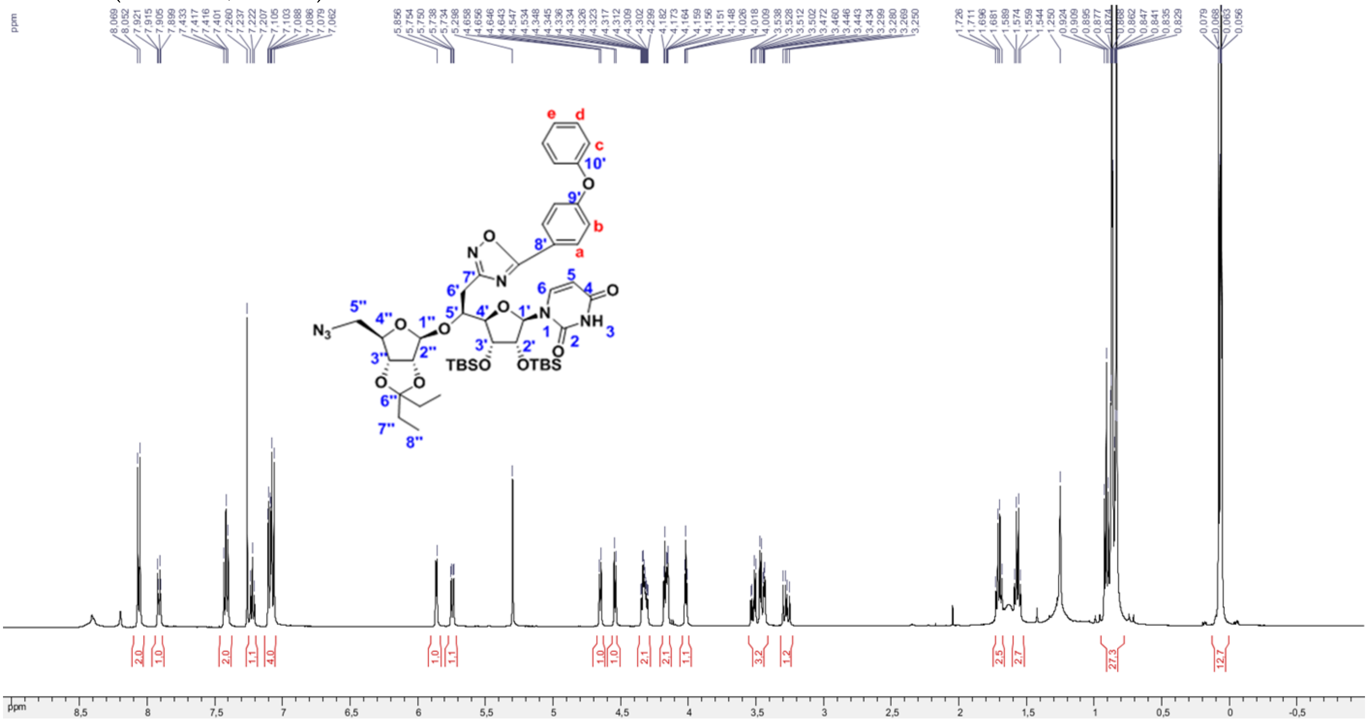

^{13}C NMR (125 MHz, $CDCl_3$)

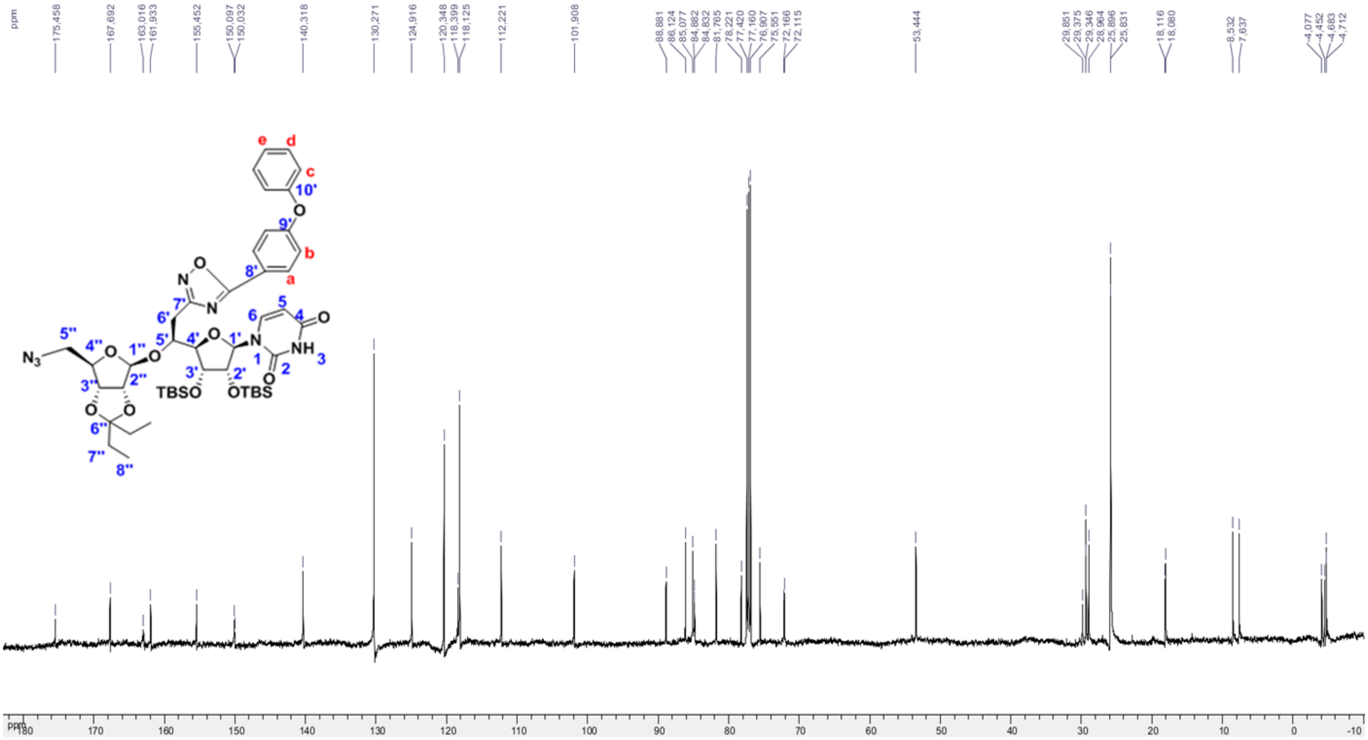

Protected Oxadiazole 11f

^1H NMR (500 MHz, $CDCl_3$)

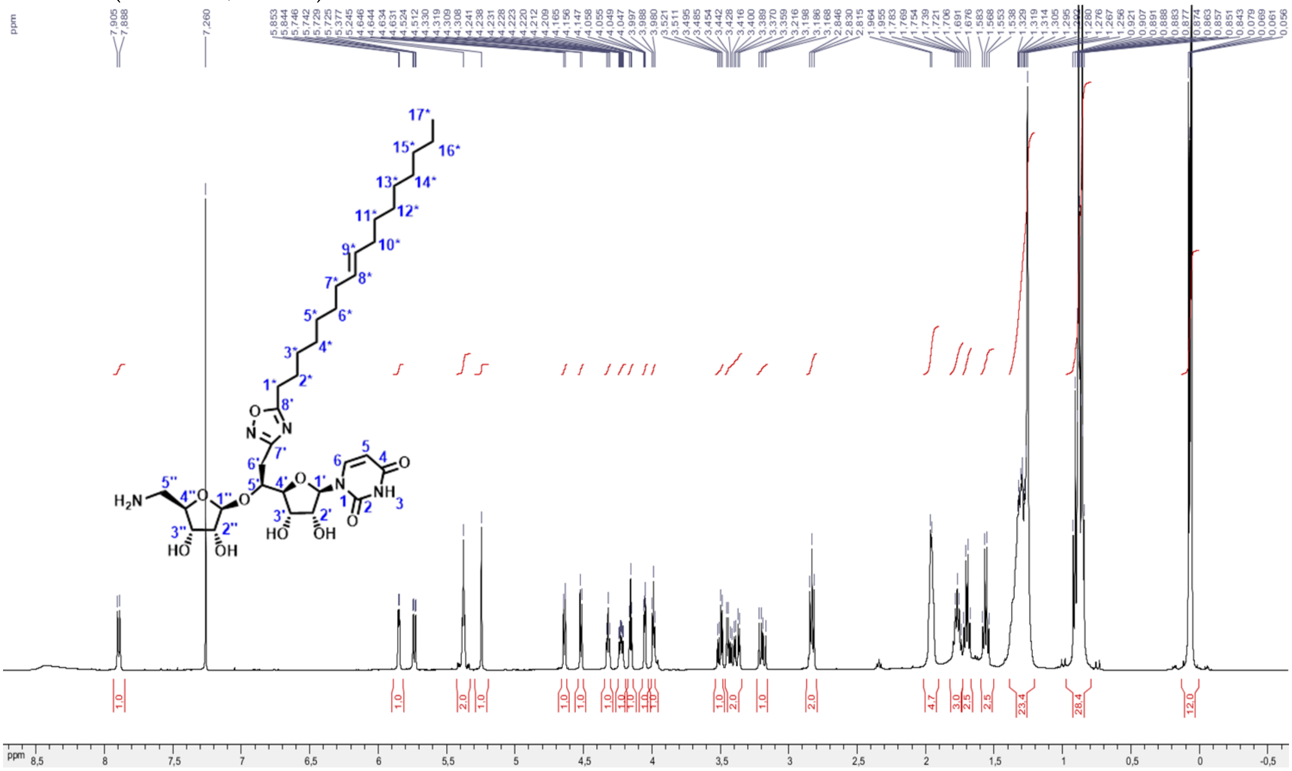

^{13}C NMR (125 MHz, $CDCl_3$)

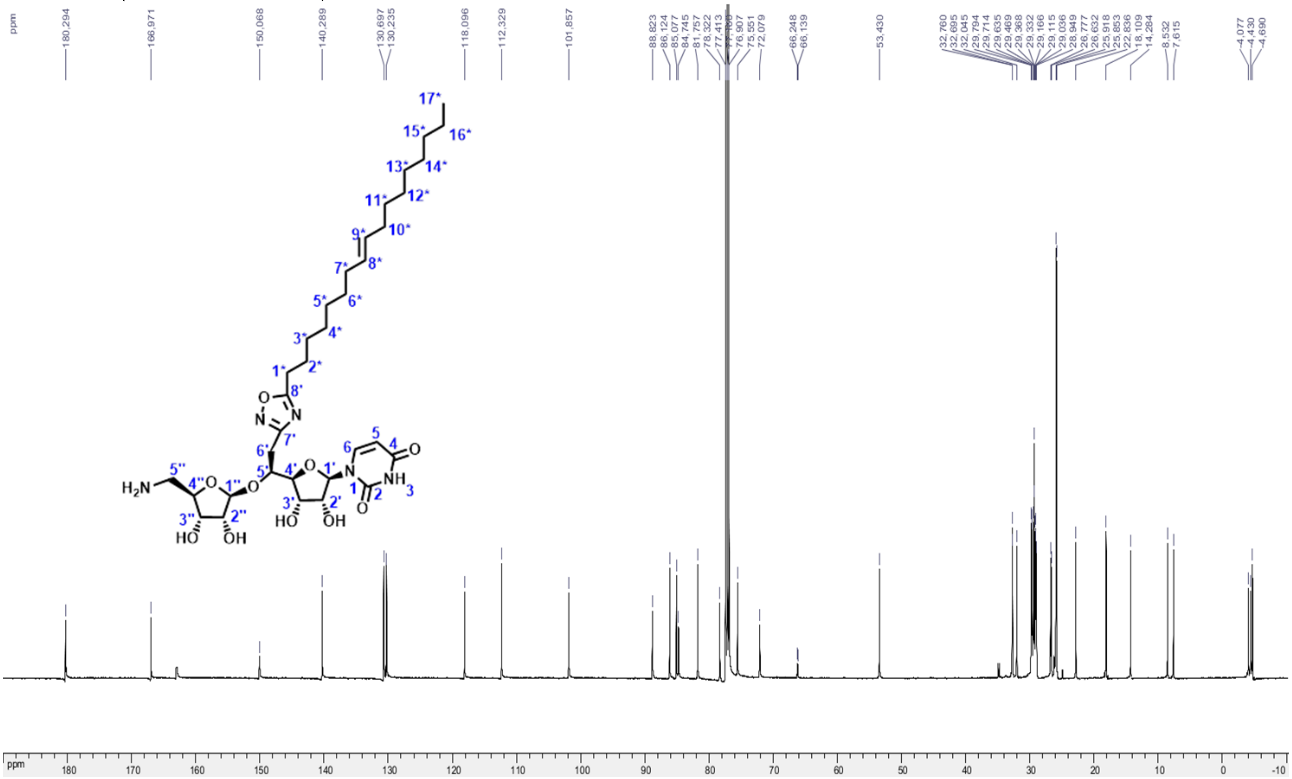

Protected Oxadiazole 11g

^{1}H NMR (500 MHz, $CDCl_3$)

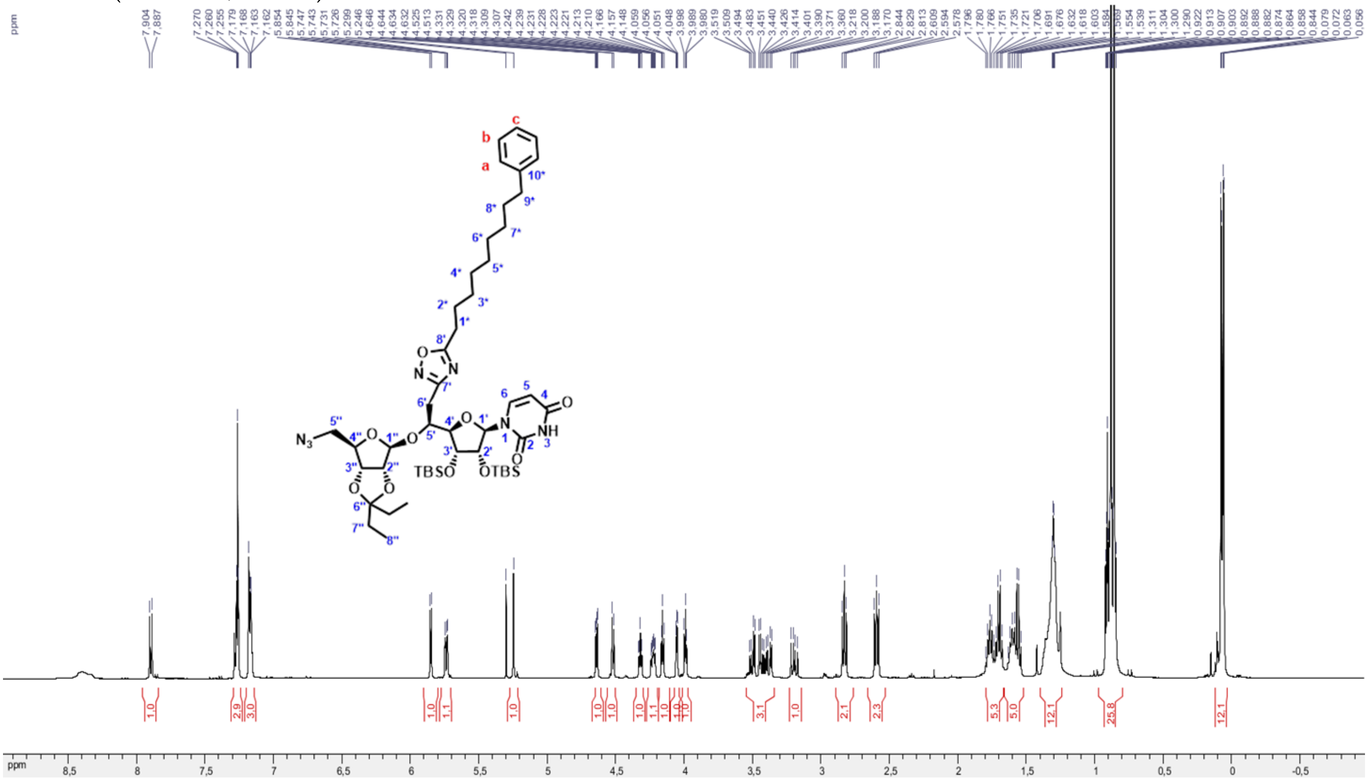

^{13}C NMR (125 MHz, $CDCl_3$)

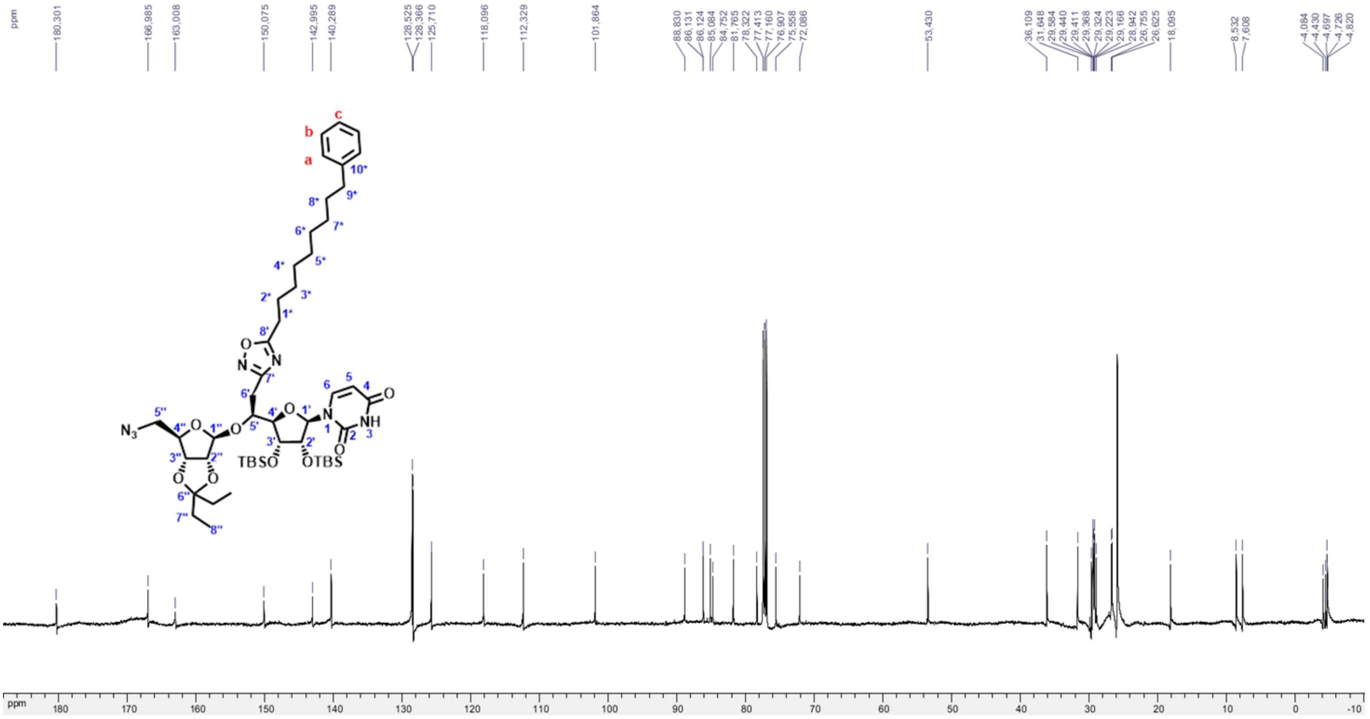

Protected Oxadiazole 11h

^{1}H NMR (500 MHz, $CDCl_3$)

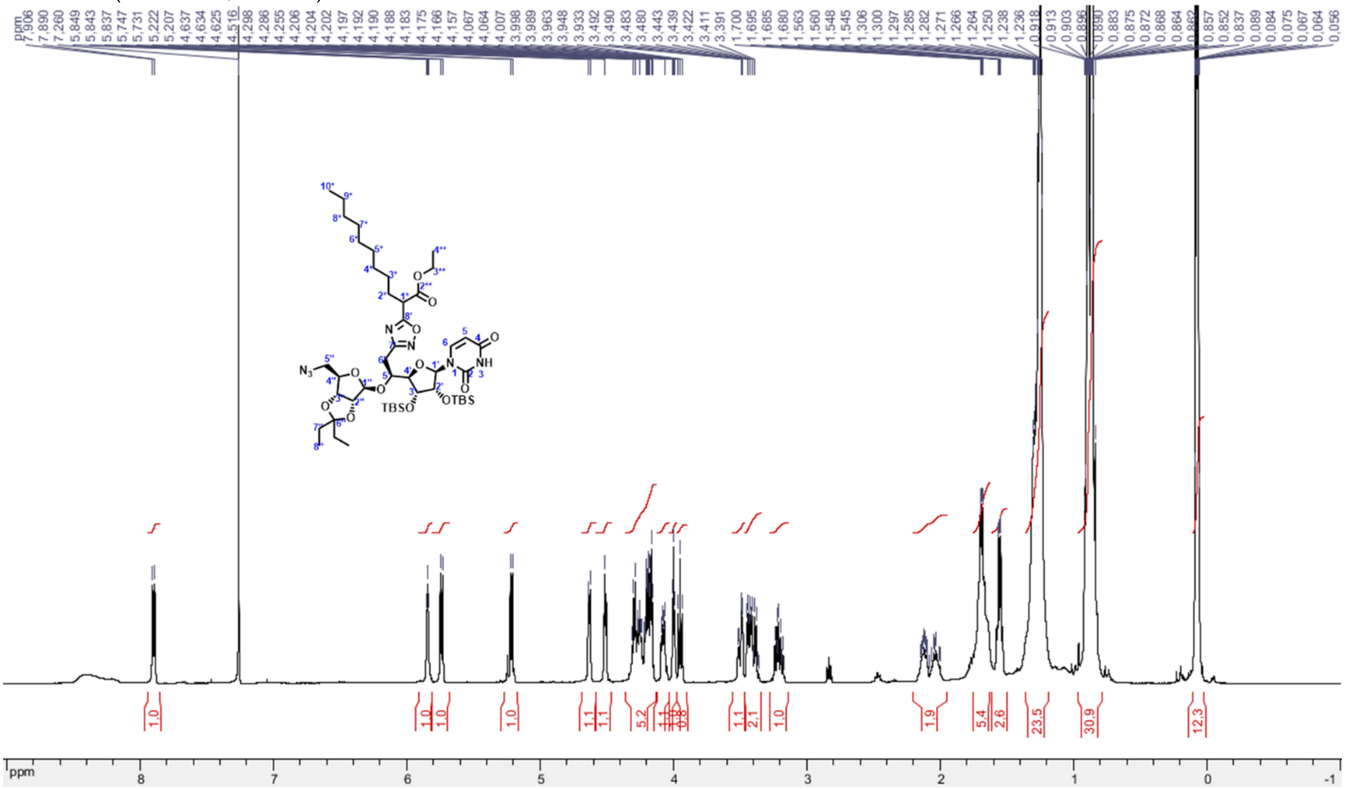

^{13}C NMR (125 MHz, $CDCl_3$)

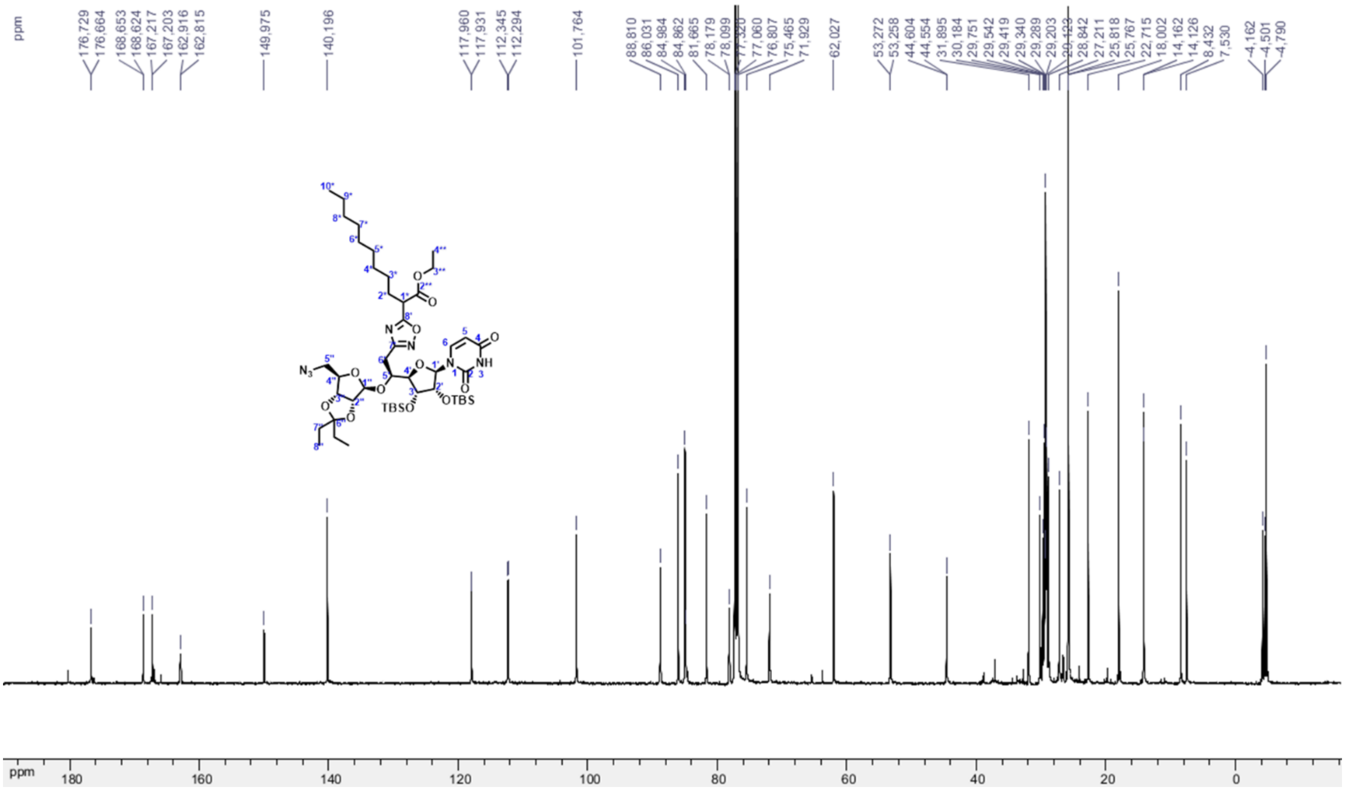

Protected Oxadiazole 11i

^{1}H NMR (500 MHz, $CDCl_3$)

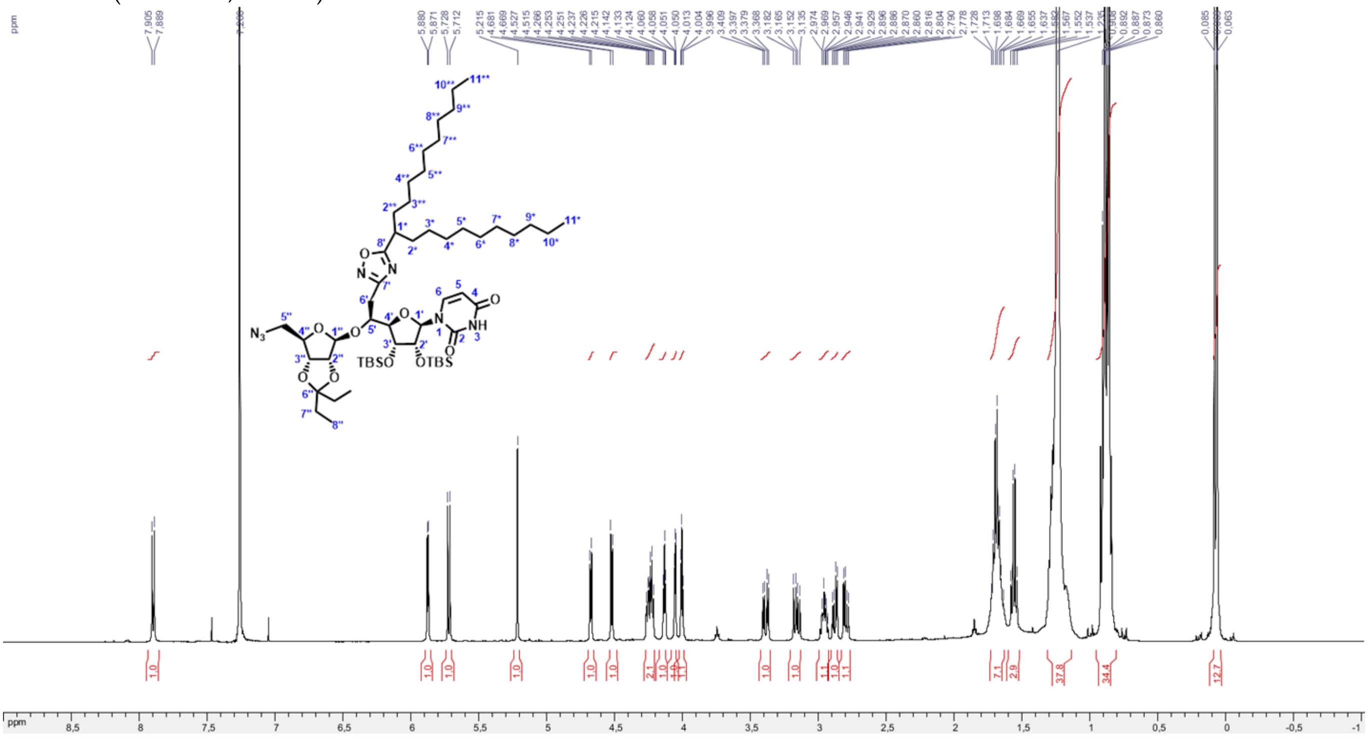

^{13}C NMR (125 MHz, $CDCl_3$)

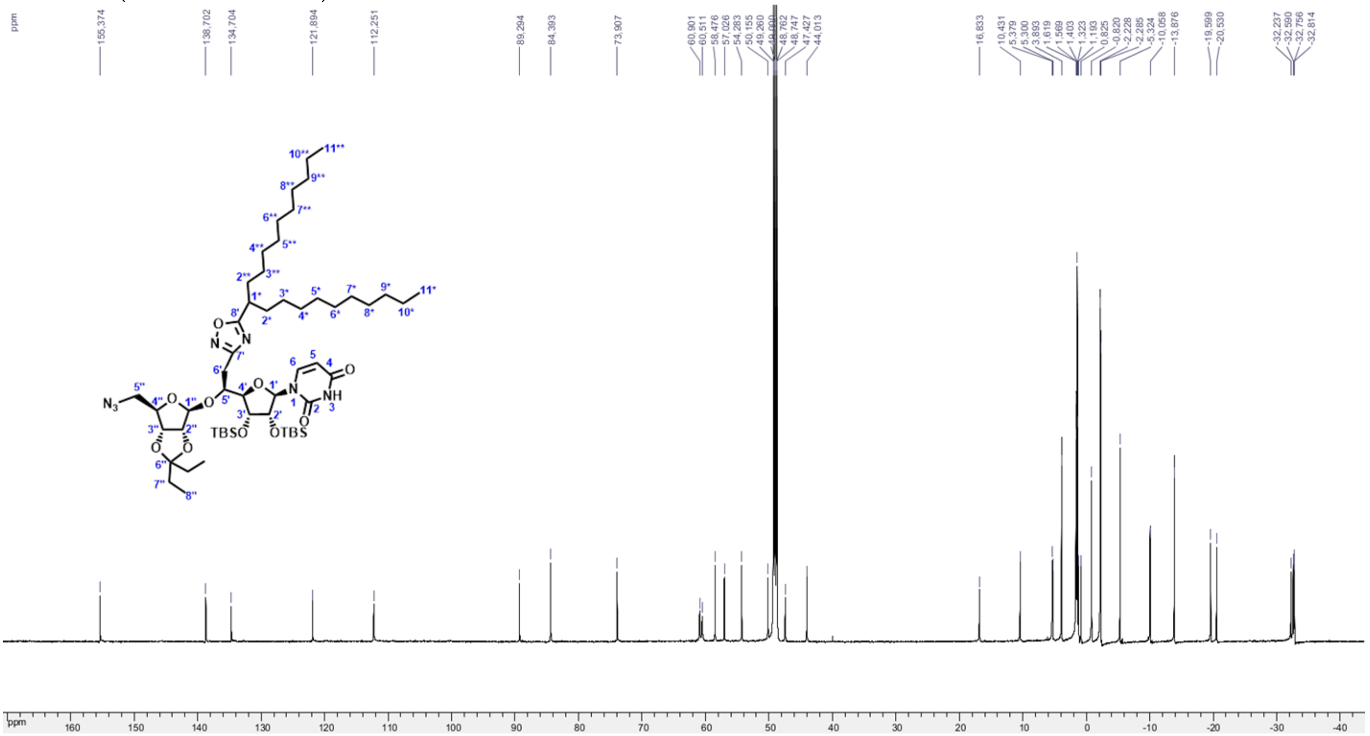

Oxadiazole 12a

^{1}H NMR (500 MHz, CD_3OD)

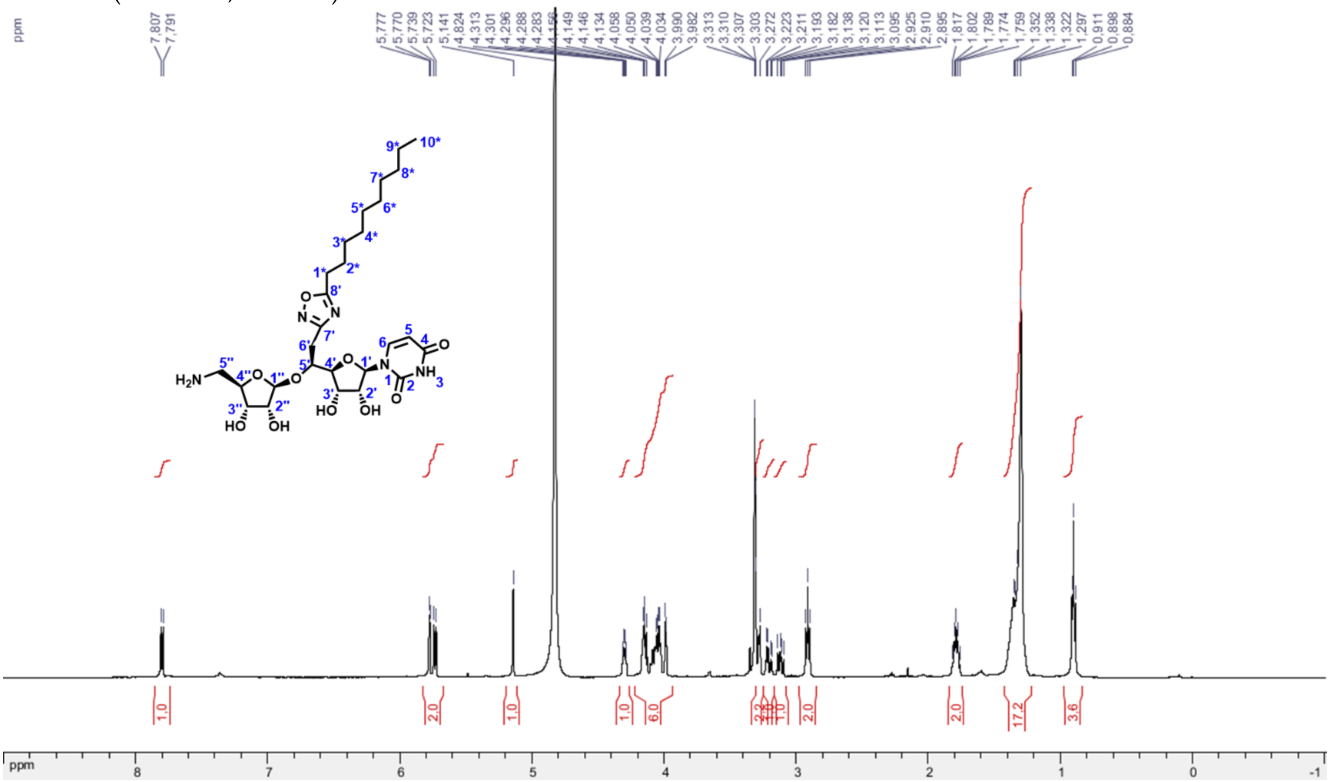

^{13}C NMR (125 MHz, $CDCl_3$)

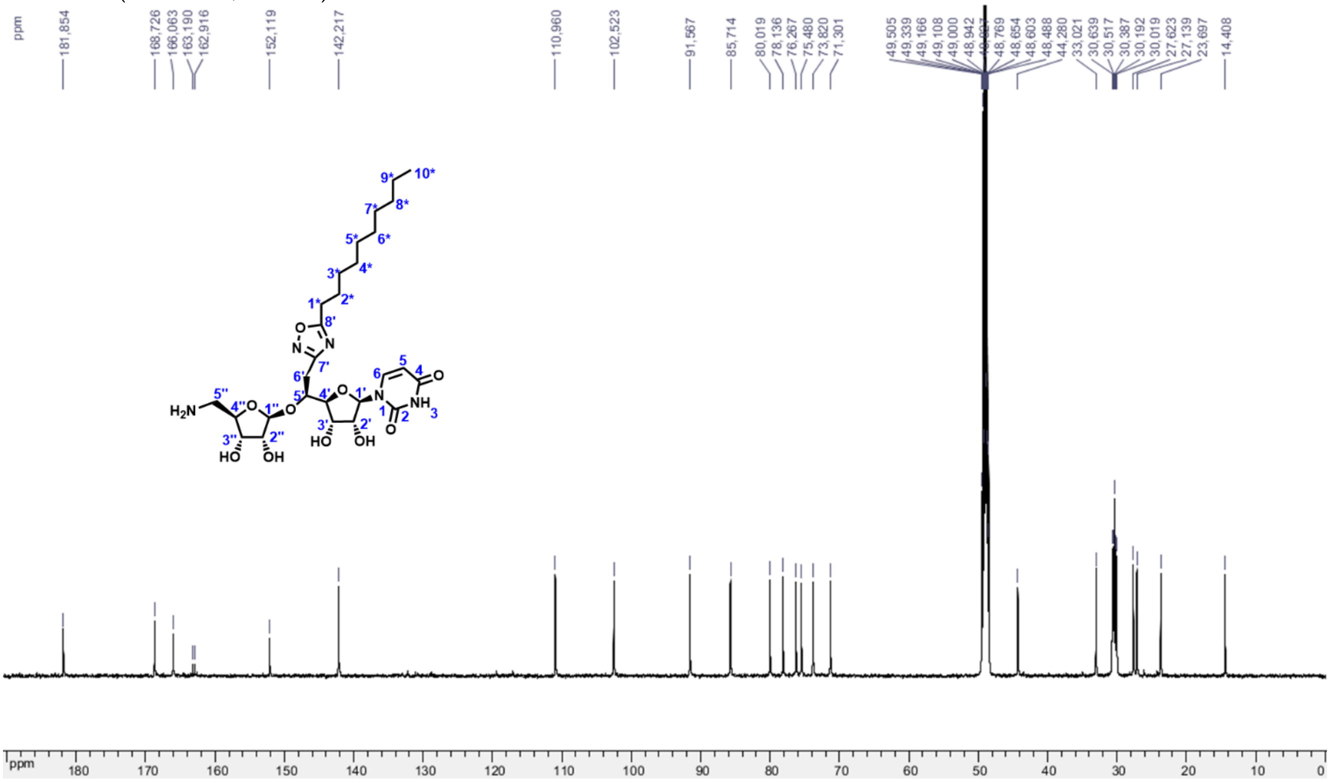

Oxadiazole 12b

^{1}H NMR (500 MHz, CD_3OD)

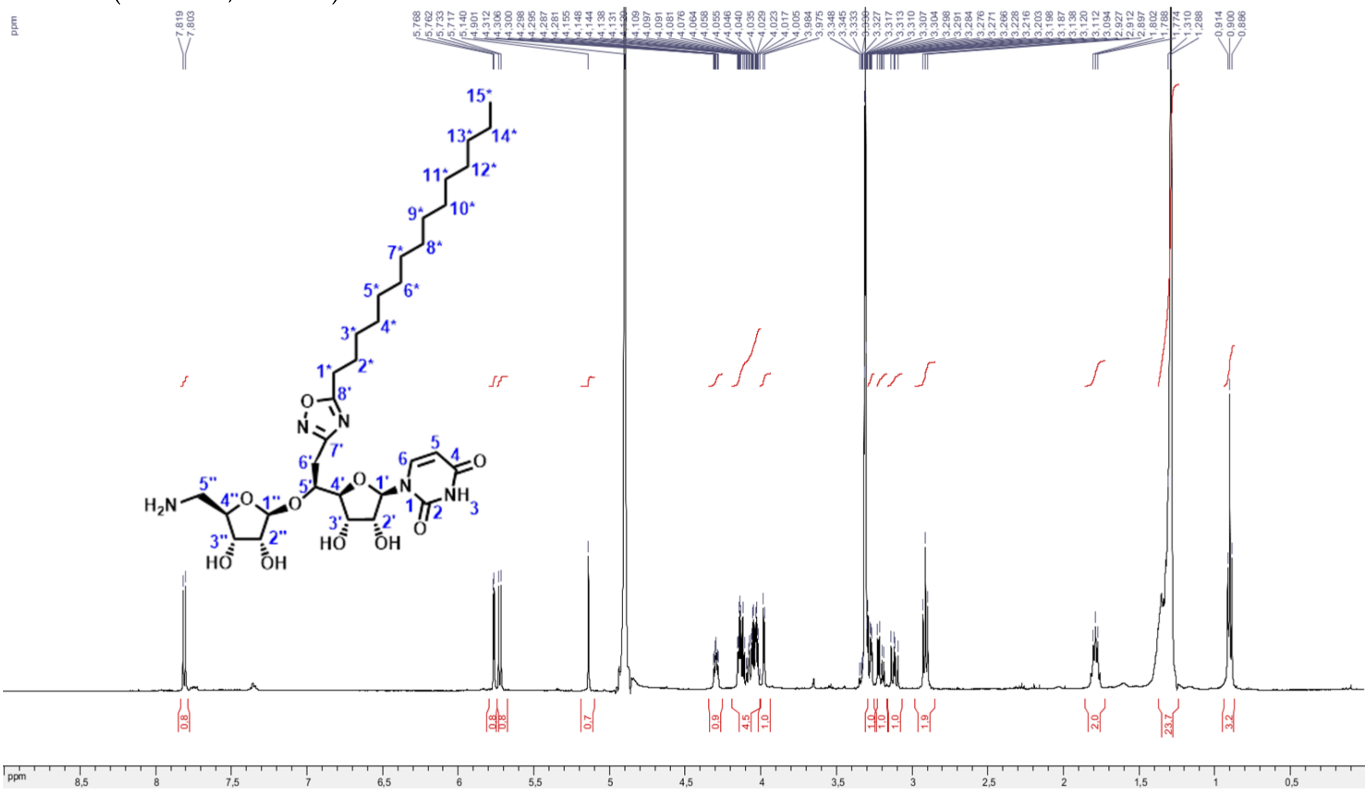

^{13}C NMR (125 MHz, CD_3OD)

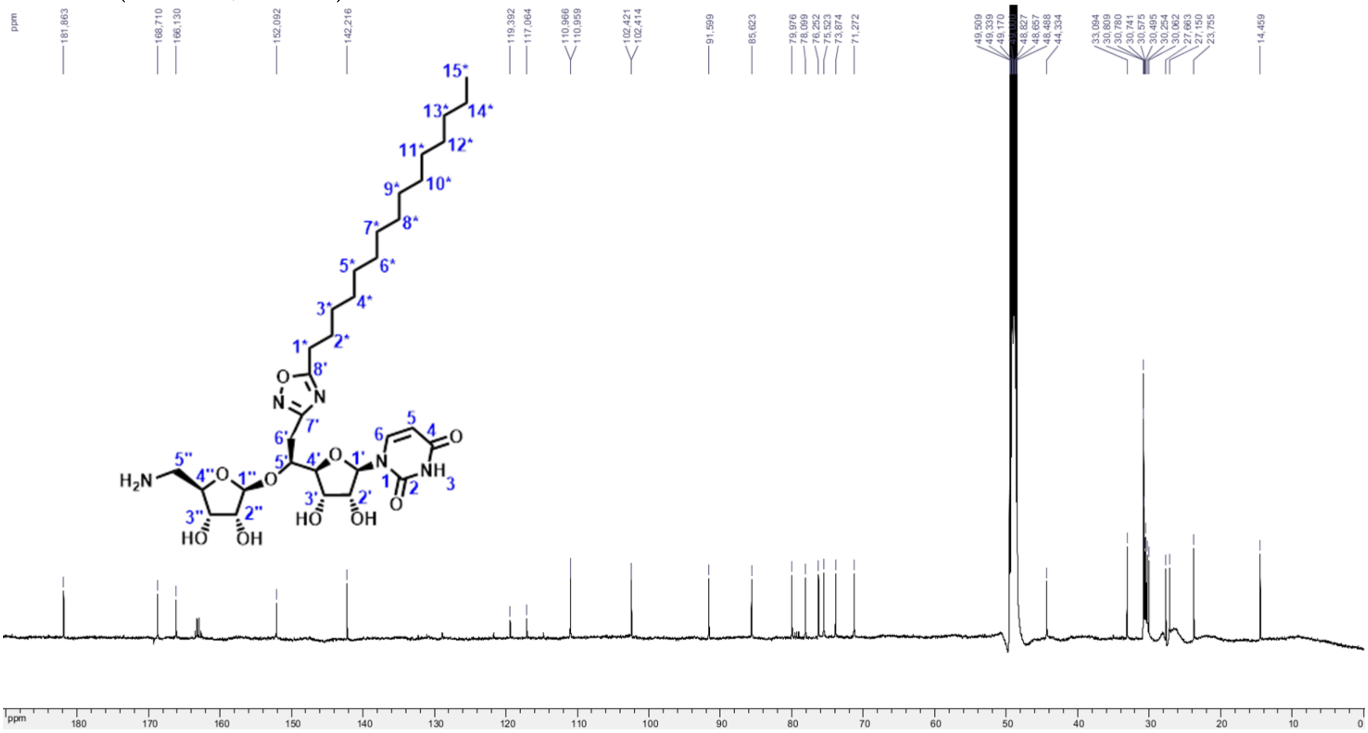

Oxadiazole 12c

^{1}H NMR (500 MHz, CD_3OD)

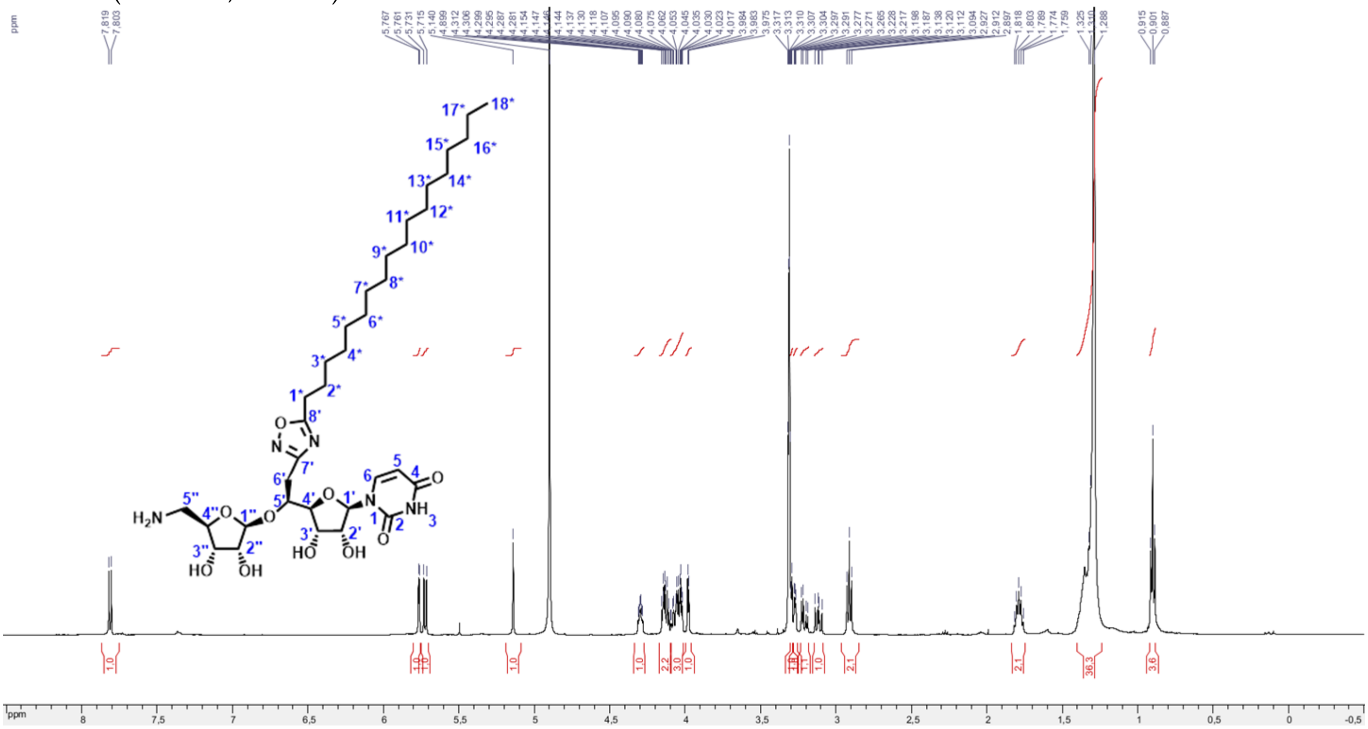

^{13}C NMR (125 MHz, CD_3OD)

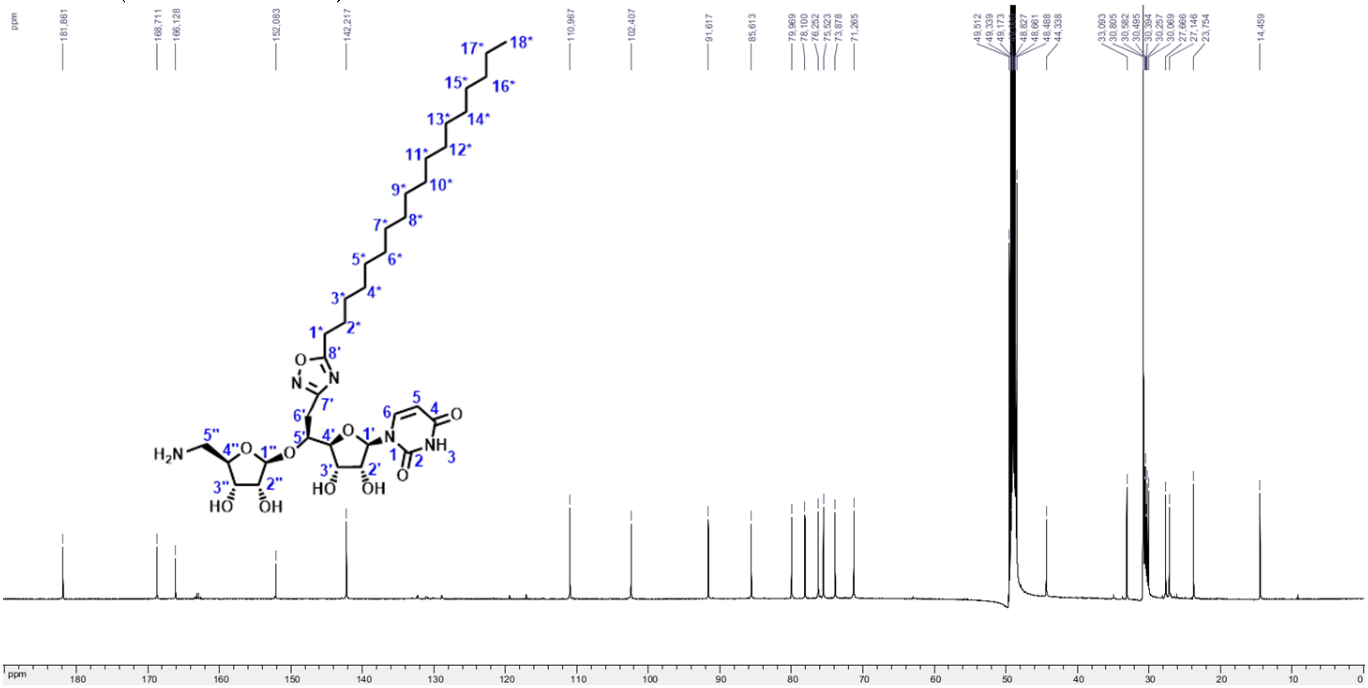

Oxadiazole 12d

^{1}H NMR (500 MHz, CD_3OD)

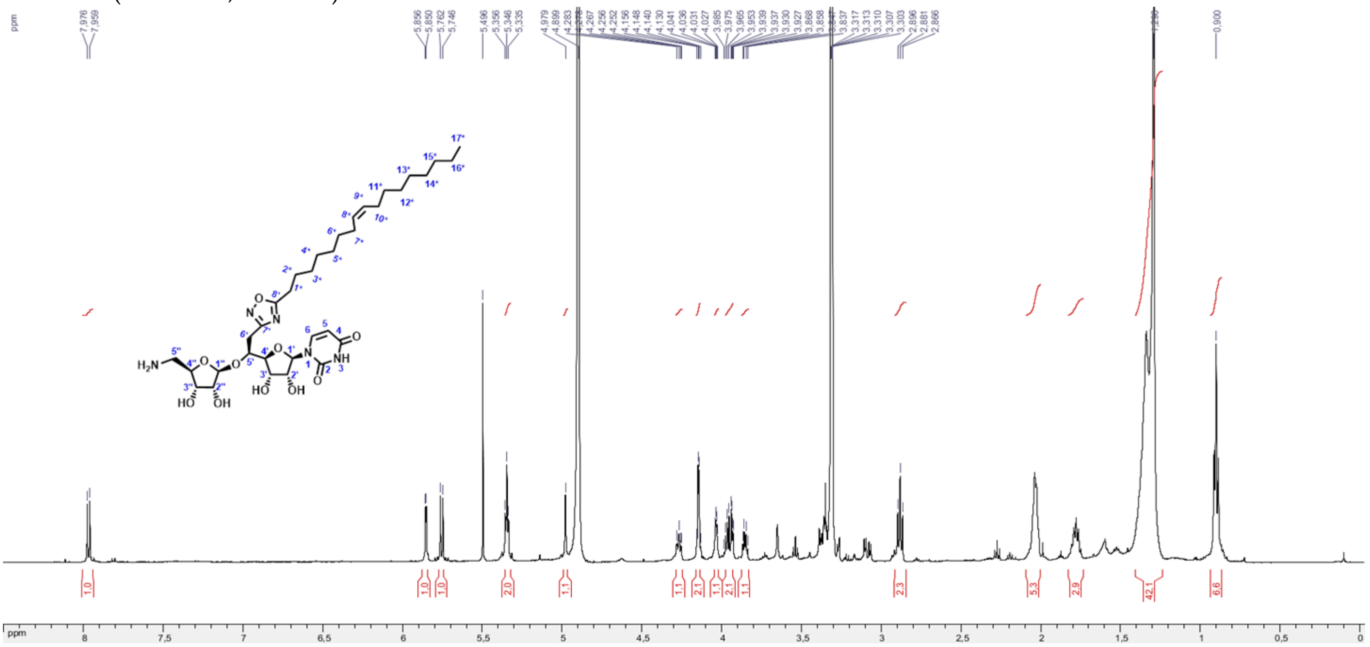

^{13}C NMR (125 MHz, CD_3OD)

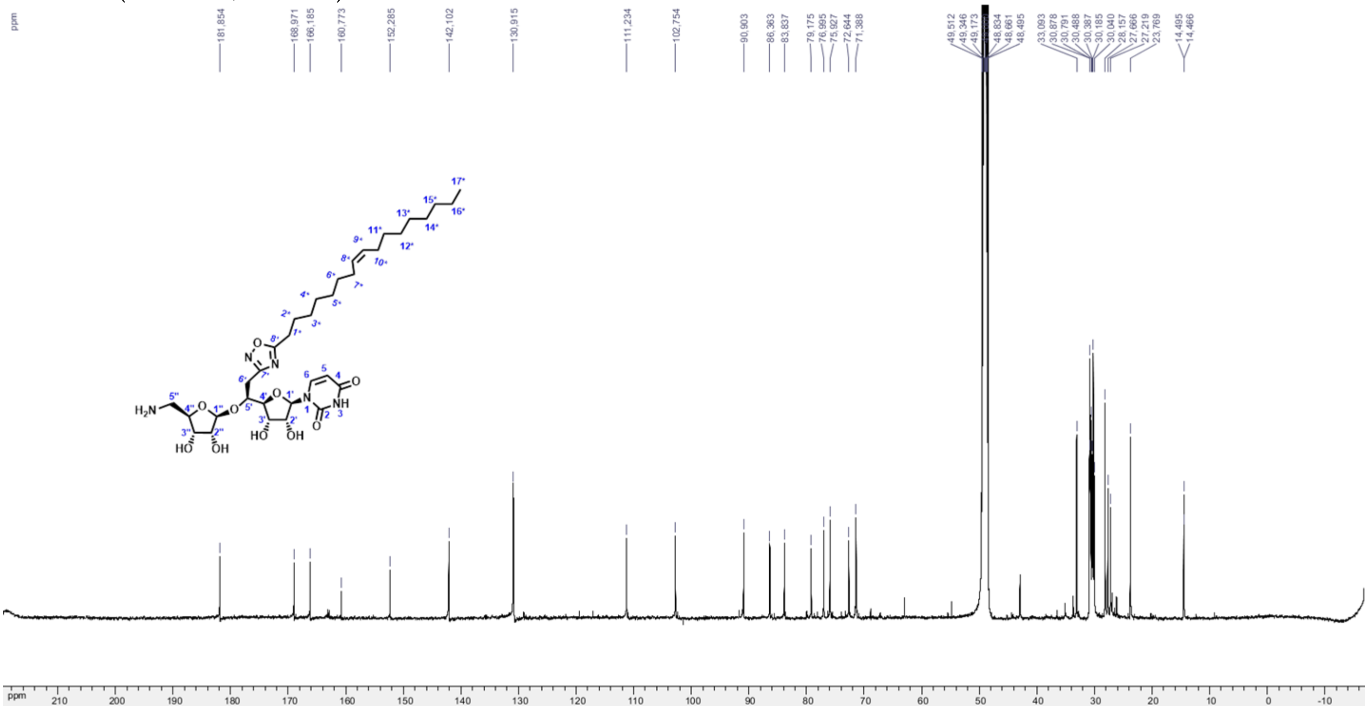

Oxadiazole 12e

^{1}H NMR (500 MHz, CD_3OD)

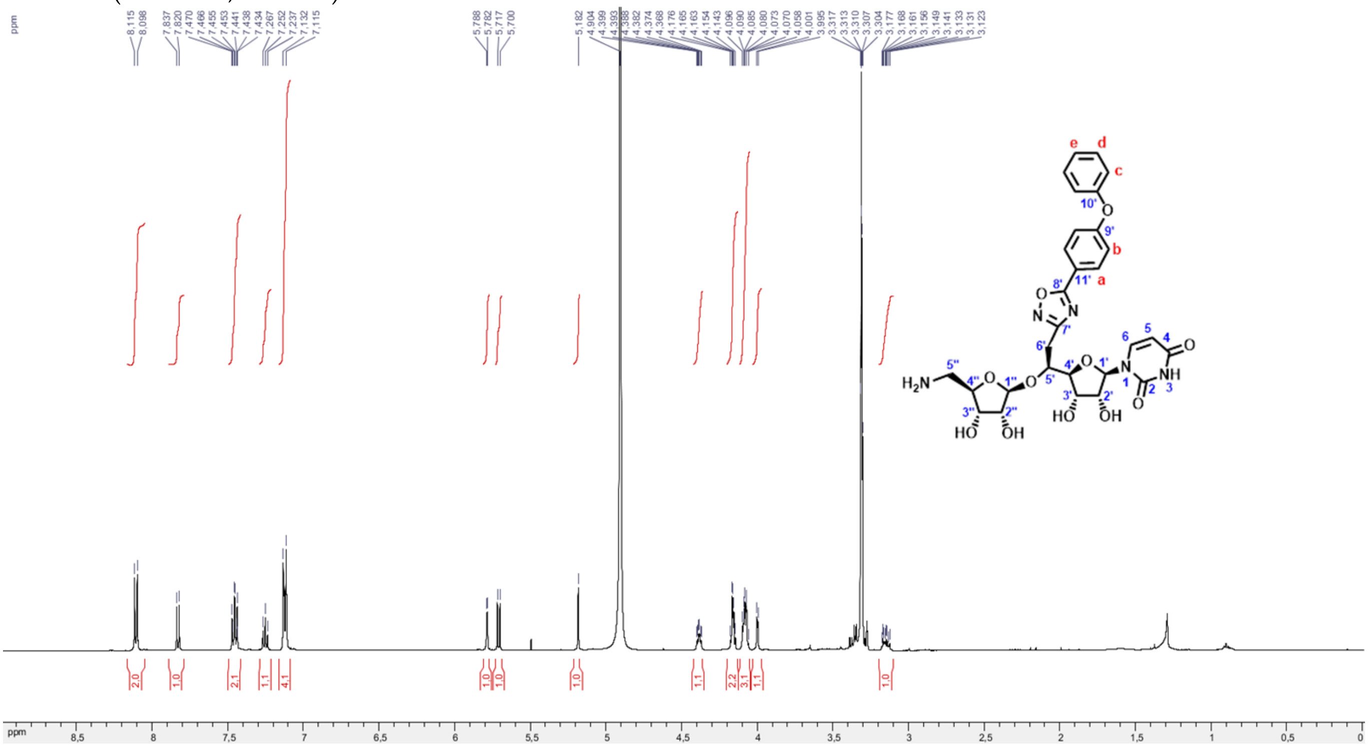

^{13}C NMR (125 MHz, CD_3OD)

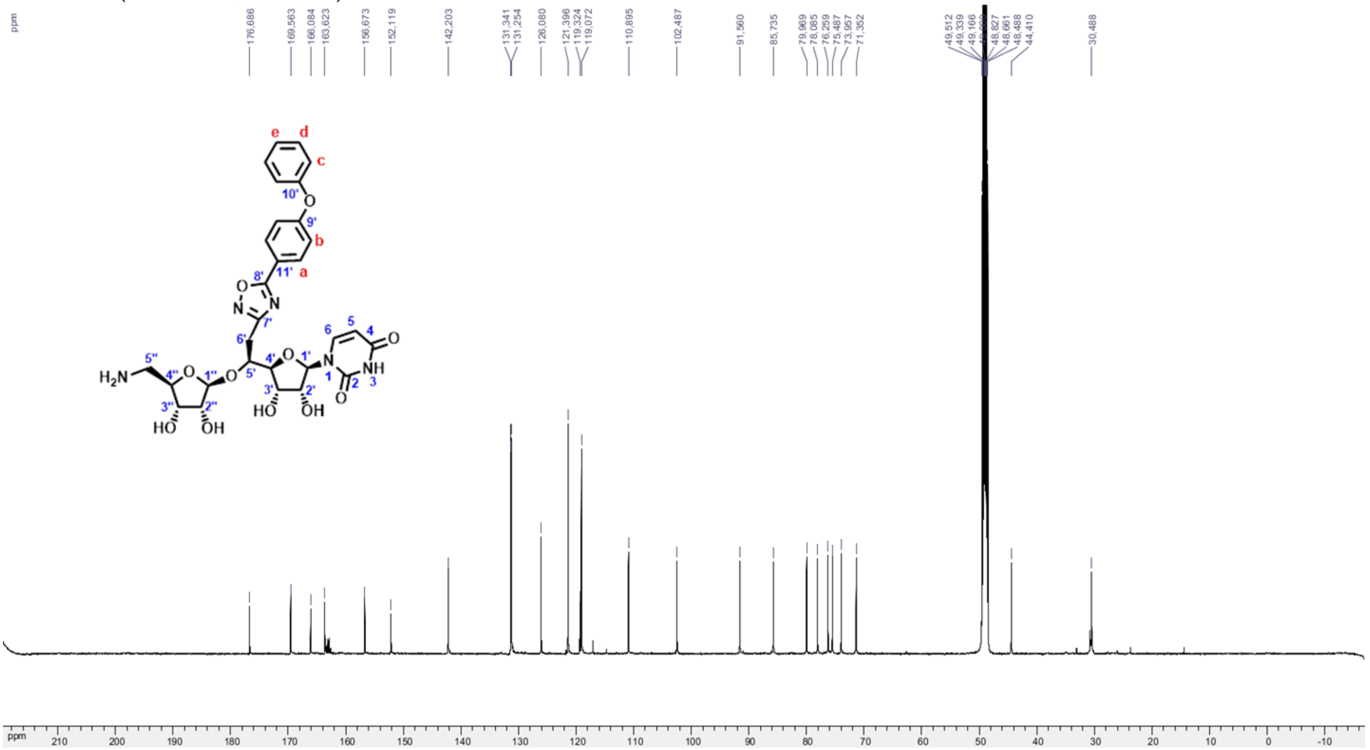

Oxadiazole 12f

^{1}H NMR (500 MHz, CD_3OD)

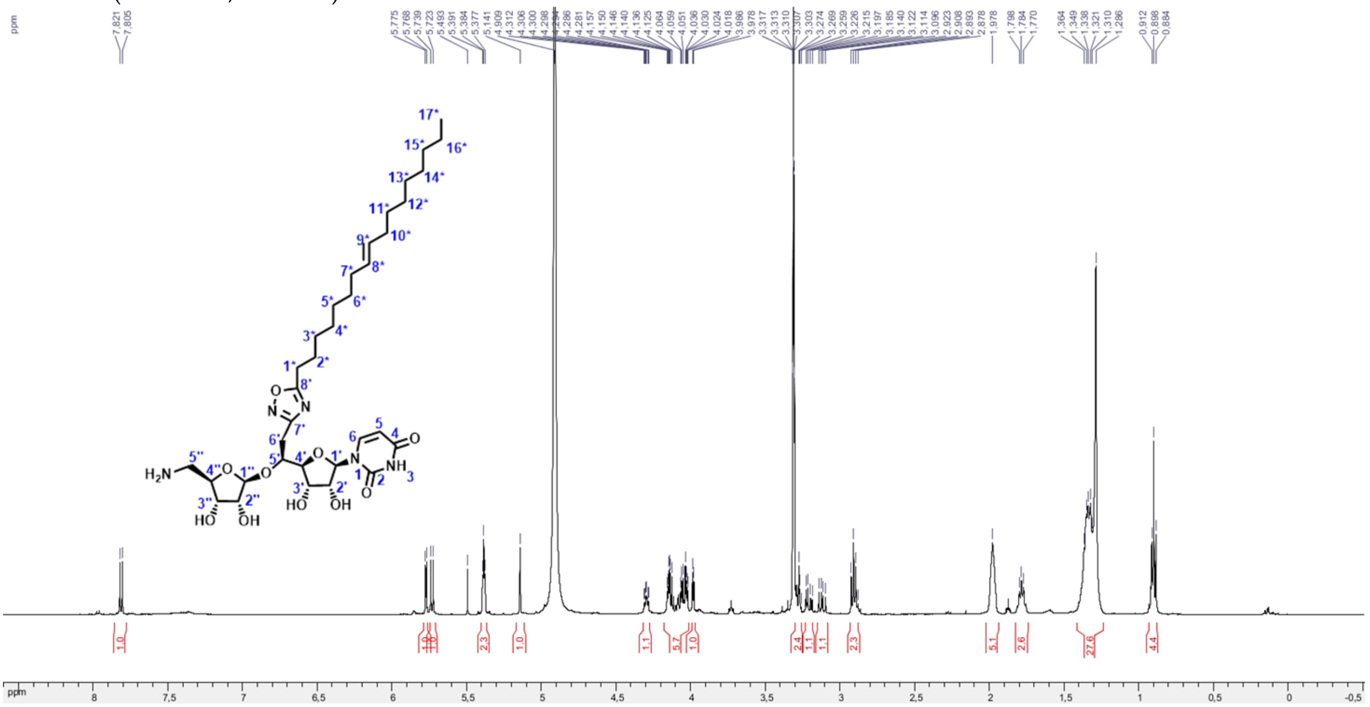

^{13}C NMR (125 MHz, CD_3OD)

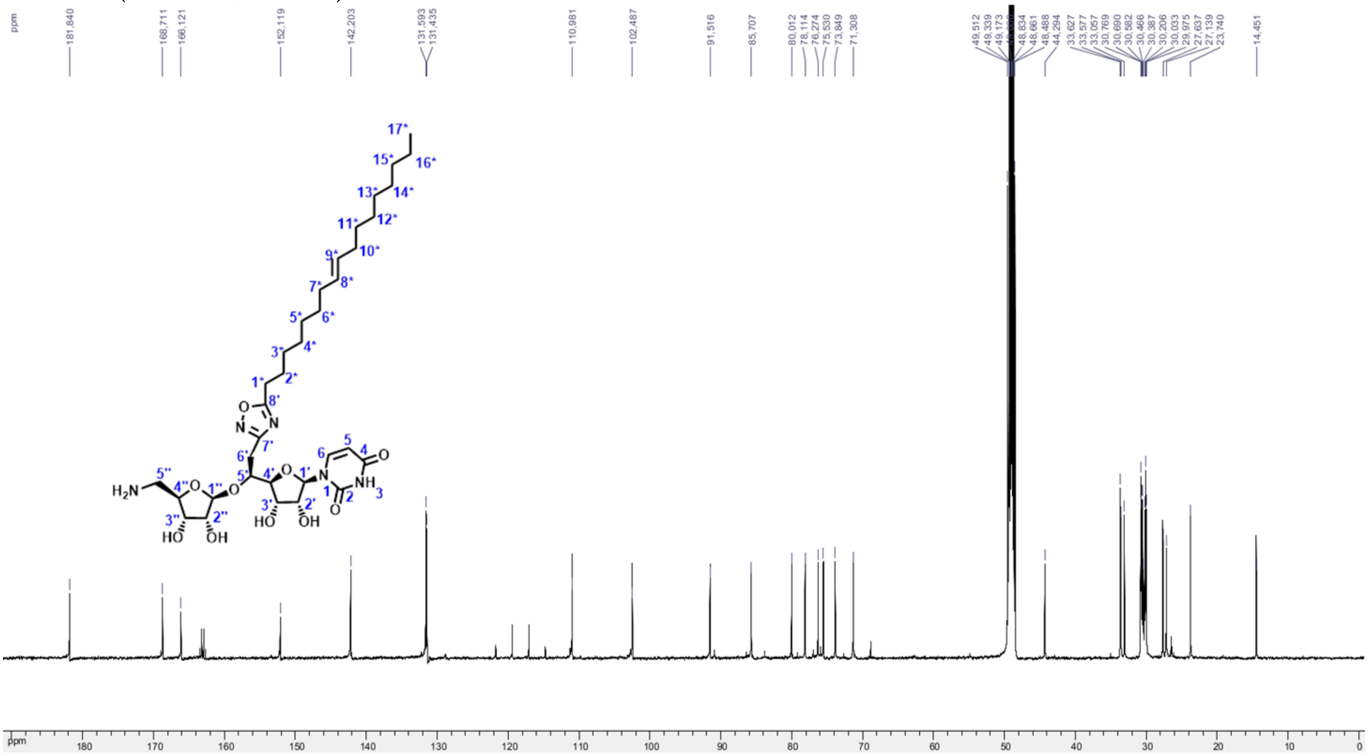

Oxadiazole 12g

^{1}H NMR (500 MHz, CD_3OD)

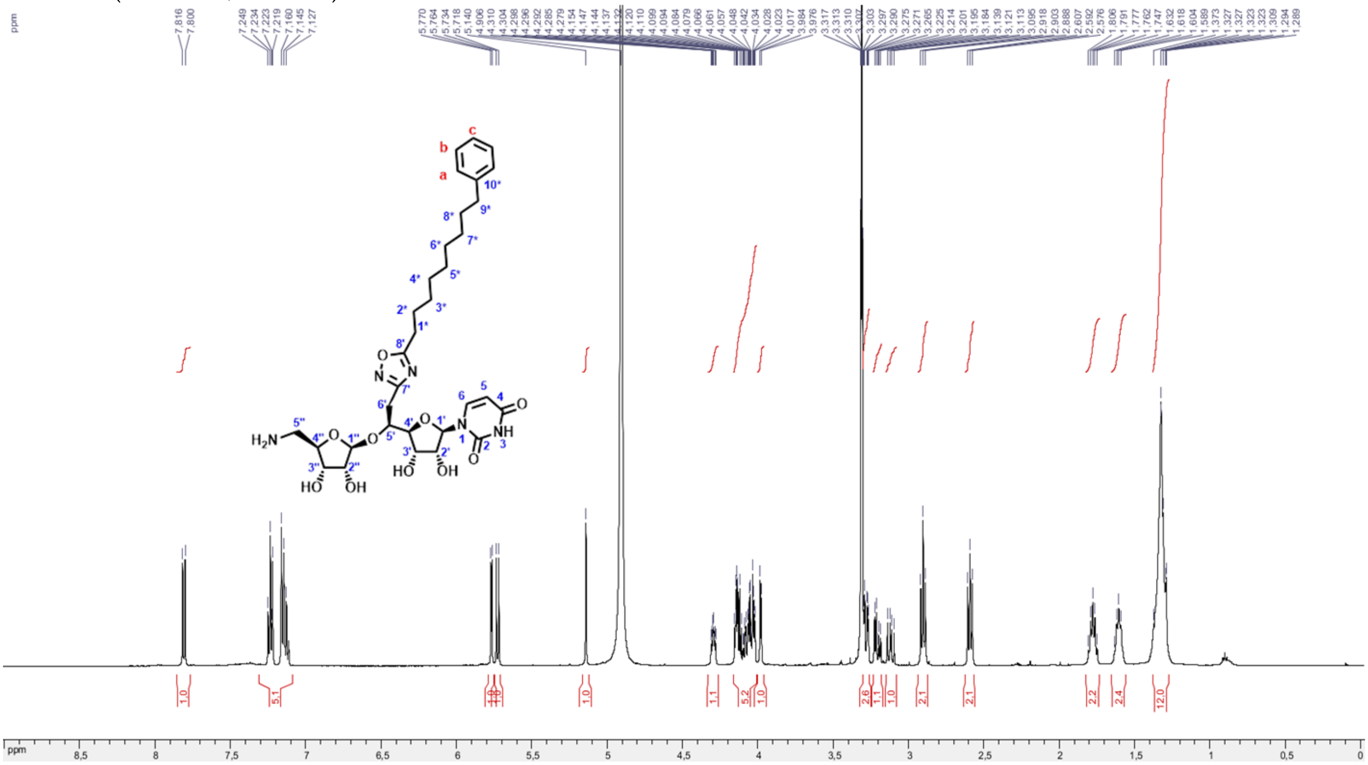

^{13}C NMR (125 MHz, CD_3OD)

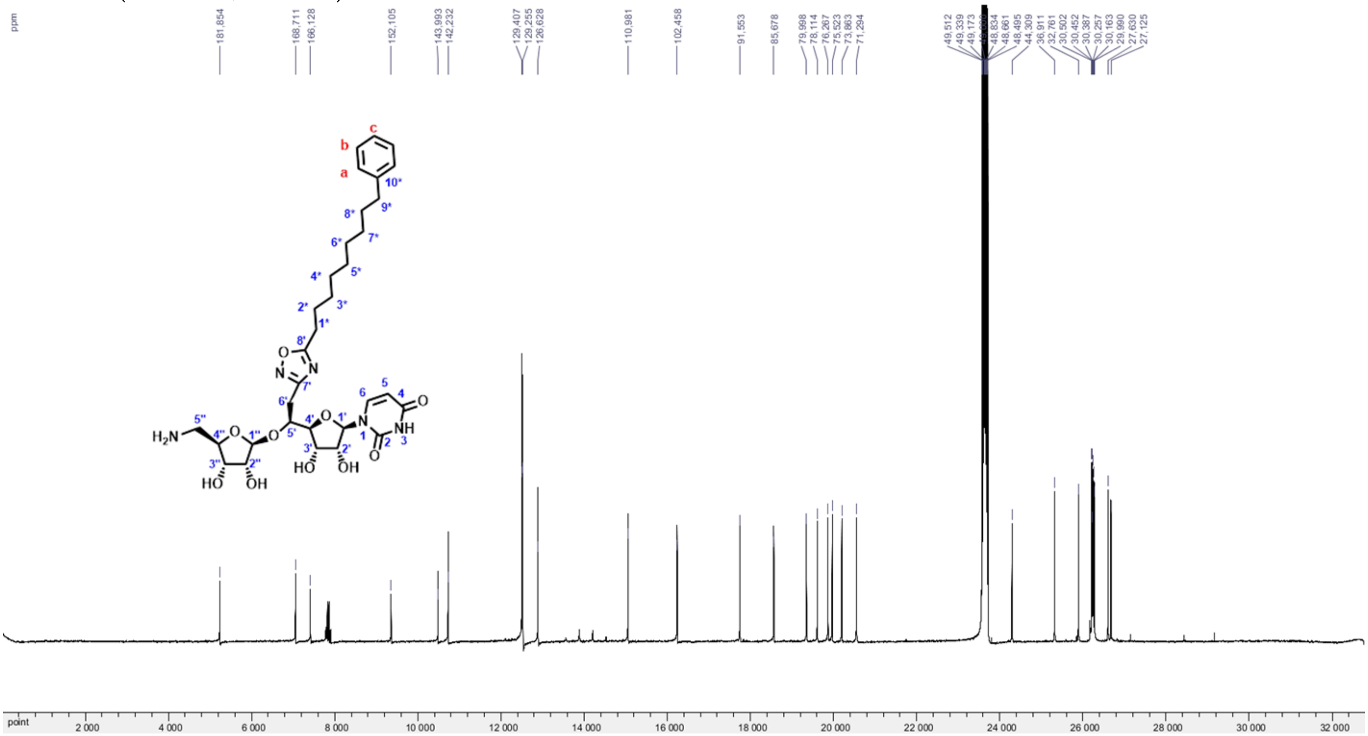

Oxadiazole 12h

^{1}H NMR (500 MHz, CD_3OD)

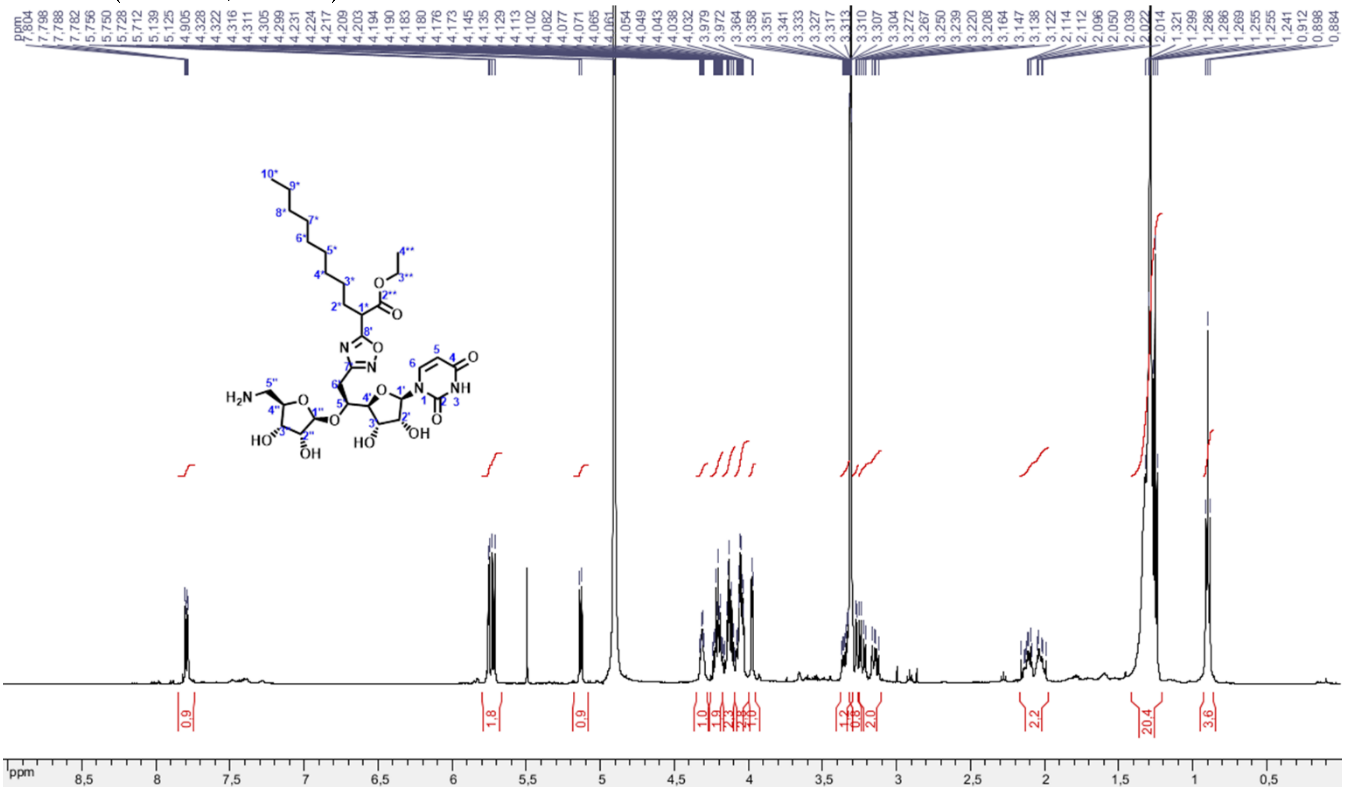

^{13}C NMR (125 MHz, CD_3OD)

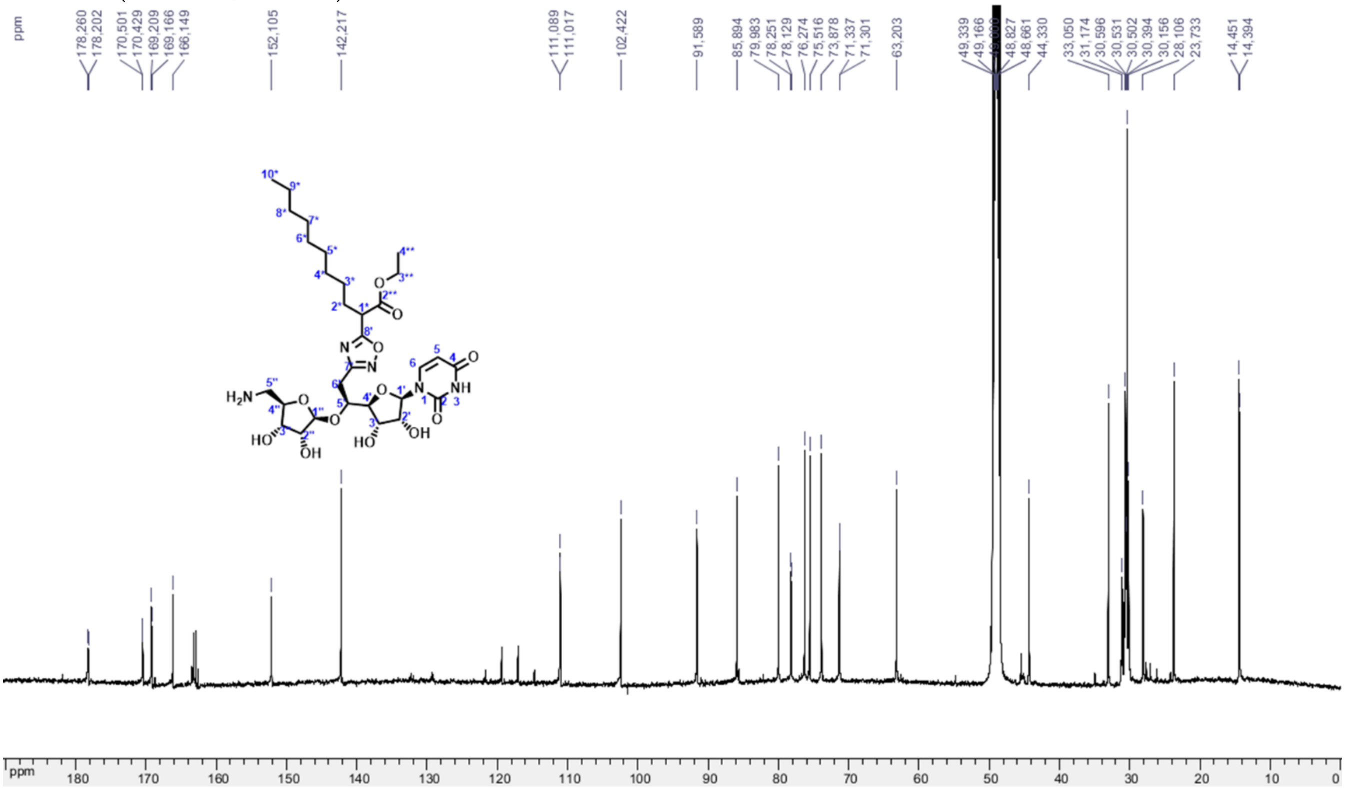

Oxadiazole 12i

^{1}H NMR (500 MHz, CD_3OD)

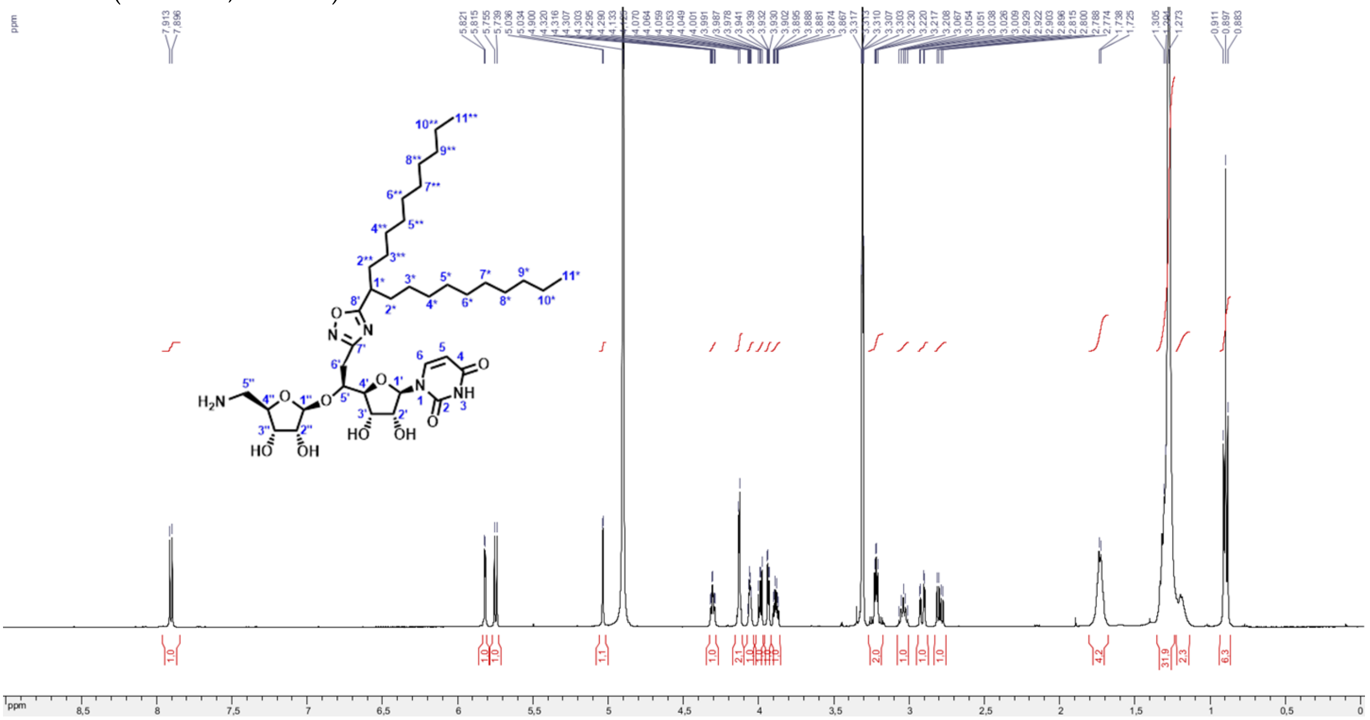

^{13}C NMR (125 MHz, CD_3OD)

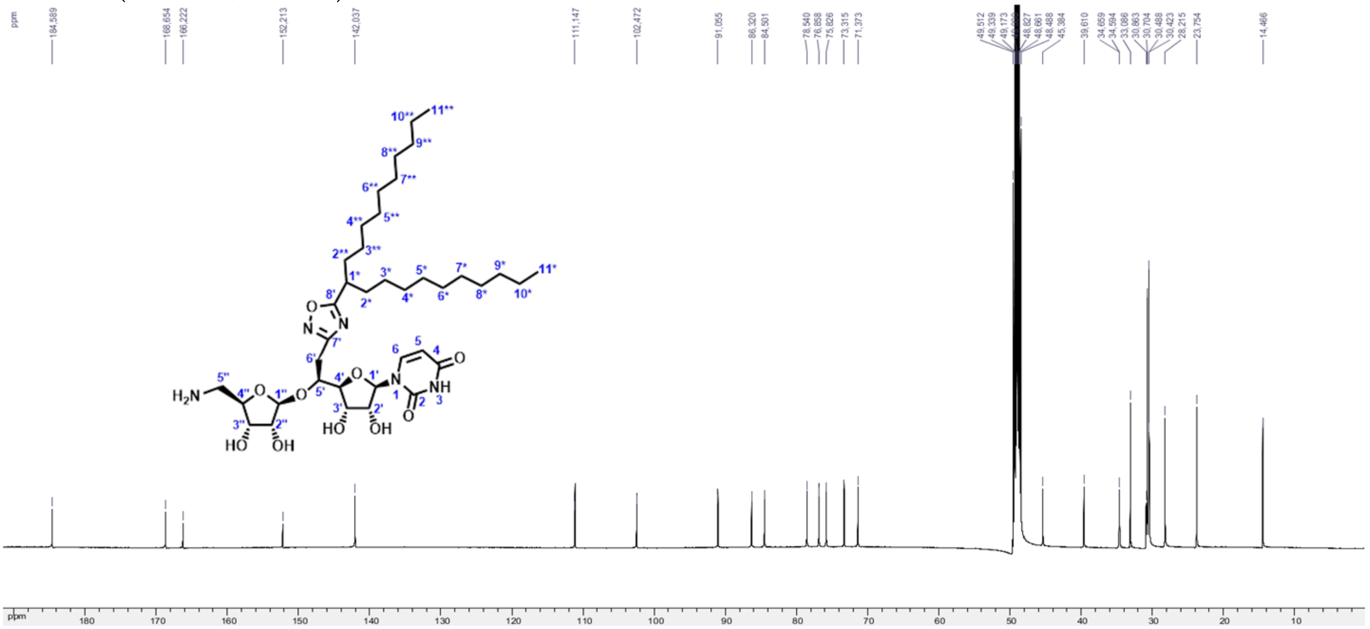

Oxadiazole 12j

^{1}H NMR (500 MHz, CD_3OD)

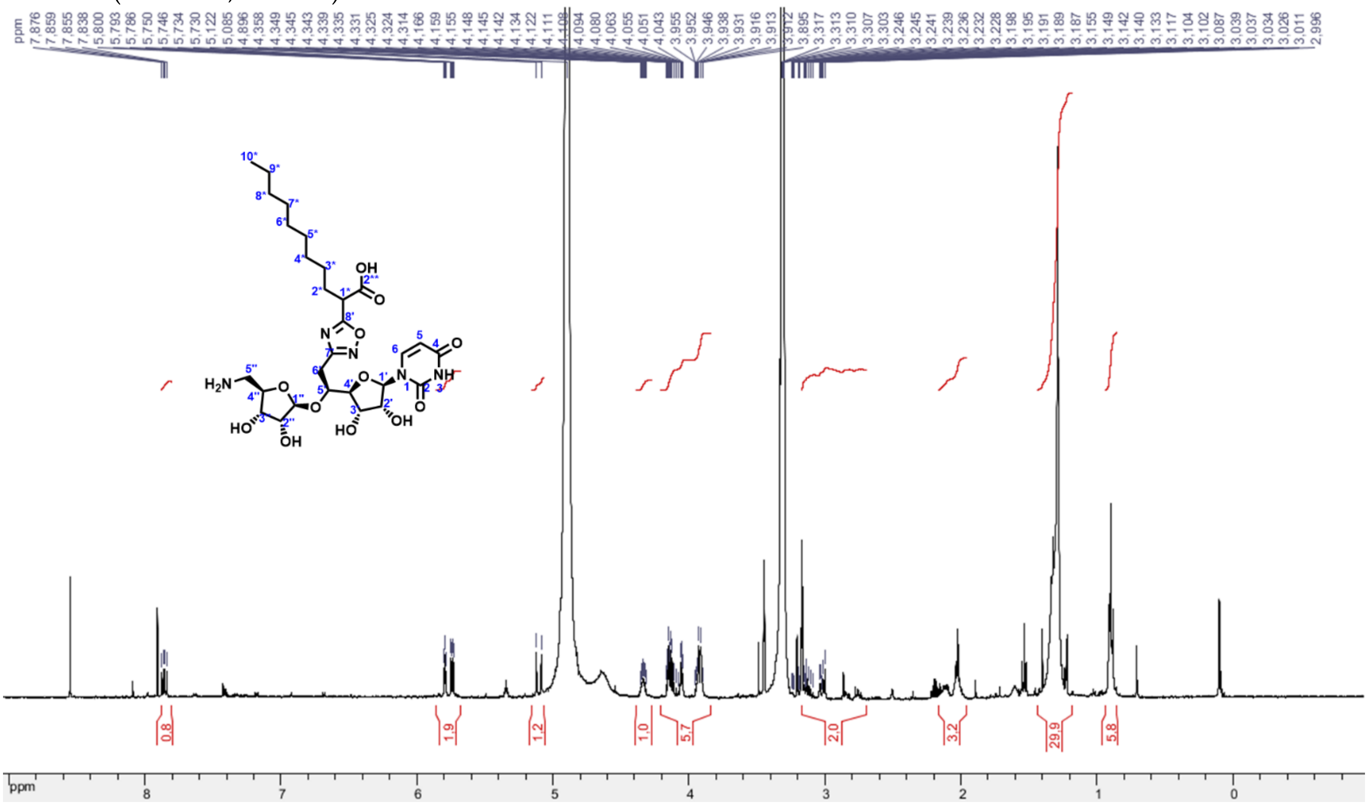

^{13}C NMR (125 MHz, CD_3OD)

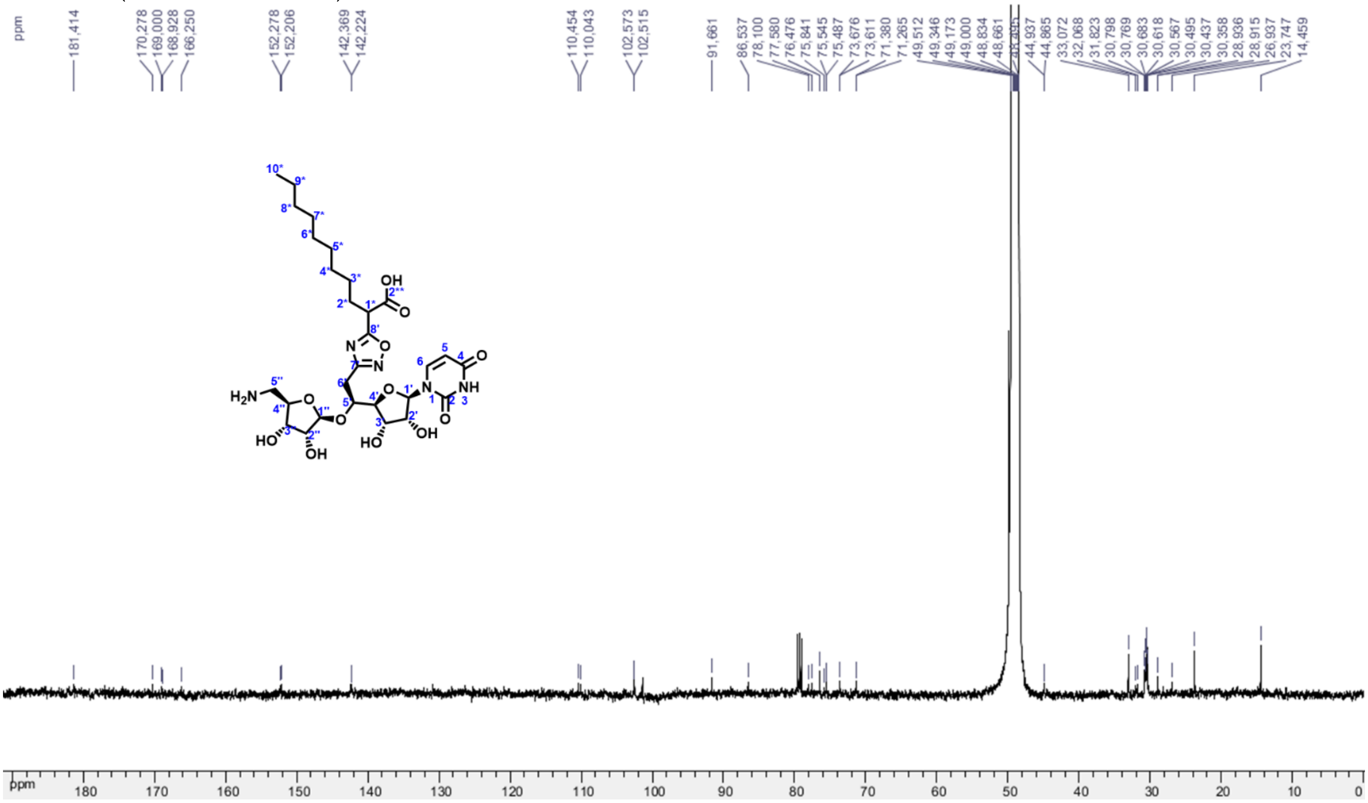

Table S1. Antibacterial activity of compounds **12a-12i**, and reference compounds.

**Compounds**	**CMI (µg/mL)**					
	Gram -			Gram +		
	Escherichia coli ATCC 8730	*Citrobacter freundii* ATCC 8090	*Pseudomonas aeruginosa* ATCC 27853	*Staphylococcus aureus* ATCC 25923	*Staphylococcus aureus* MRSA ATCC 43300	*Enterococcus faecium* ATCC 19434
**12a**	>50	>50	>50	>50	>50	>50
**12b**	>50	>50	>50	>50	> 50	>50
**12c***	>50	>50	>50	50	50	50
**12d***	>50	>50	>50	>50	>50	>50
**12e**	>50	>50	>50	>50	>50	>50
**12f***	>50	>50	>50	50	50	50
**12g**	>50	>50	>50	>50	>50	>50
**12h**	>50	>50	>50	> 50	> 50	> 50
**12i***	>50	>50	>50	> 50	> 50	> 50
**Piperacillin**	4	4	8	4	>128	4
**Vancomycin**	-	-	-	1	1	0.5

* Indicates that the molecule is insoluble in 5%DMSO-water (culture media) at the highest final concentration tested
